# Supplementary material for: Omada: robust clustering of transcriptomes through multiple testing
Source: Gigascience. 2024 Jul 11;13:giae039. doi: 10.1093/gigascience/giae039 (PMC11238428; doi:10.1093/gigascience/giae039)
Supplement: giae039_GIGA-D-23-00220_Revision_2 [file giae039_giga-d-23-00220_revision_2.pdf]

GigaScience

Omada: Robust clustering of transcriptomes through multiple testing

--Manuscript Draft--

**Manuscript Number:**	GIGA-D-23-00220R2
**Full Title:**	Omada: Robust clustering of transcriptomes through multiple testing
**Article Type:**	Research
**Funding Information:**	Academy of Medical Sciences (APR7_1002) — Prof Dennis Wang<br>Engineering and Physical Sciences Research Council (EP/V029045/1) — Prof Dennis Wang
**Abstract:**	Cohort studies increasingly collect biosamples for molecular profiling and are observing molecular heterogeneity. High throughput RNA sequencing is providing large datasets capable of reflecting disease mechanisms. Clustering approaches have produced a number of tools to help dissect complex heterogeneous datasets, however, selecting the appropriate method and parameters to perform exploratory clustering analysis of transcriptomic data requires deep understanding of machine learning and extensive computational experimentation. Tools that assist with such decisions without prior field knowledge are nonexistent. To address this we have developed Omada, a suite of tools aiming to automate these processes and make robust unsupervised clustering of transcriptomic data more accessible through automated machine learning based functions. The efficiency of each tool was tested with seven datasets characterised by different expression signal strengths to capture a wide spectrum of RNA expression datasets. Our toolkit's decisions reflected the real number of stable partitions in datasets where the subgroups are discernible. Within datasets with less clear biological distinctions, our tools either formed stable subgroups with different expression profiles and robust clinical associations or revealed signs of problematic data such as biased measurements. Implementation of Omada is available at https://bioconductor.org/packages/omada.
**Corresponding Author:**	Dennis Wang, PhD<br>Imperial College London<br>London, UNITED KINGDOM
**Corresponding Author Secondary Information:**	
**Corresponding Author's Institution:**	Imperial College London
**Corresponding Author's Secondary Institution:**	
**First Author:**	Sokratis Kariotis
**First Author Secondary Information:**	
**Order of Authors:**	Sokratis Kariotis
	Pei fang Tan
	Haiping Lu
	Christopher J Rhodes
	Martin R Wilkins
	Allan Lawrie
	Dennis Wang, PhD
**Order of Authors Secondary Information:**	
**Response to Reviewers:**	Dear Editor,<br><br>Thank you for the opportunity to respond to the single reviewer's comments. We have

addressed all their comments and made extensive edits to the text to satisfy the reviewer’s preferences for writing style. These include:
- Substantial re-writing of the Introduction and Discussion to present the paper more as an article describing a software package.
- More details in all sections about the purpose, rationale behind parameter choices, benchmarking across multiple different applications, and discussion about context.
- Additional functions for consensus clustering and metric for measuring agreement between multiple clustering outputs
- Additional analysis of cancer data showing agreement between clustering algorithms and parameters.

A clear point-by-point response with highlighting of the changes made can be viewed in the Personal Cover letter at the end of this submission. We hope these extensive amendments will satisfy your criteria for publication in Gigascience.

Best regards,

Dennis Wang
(on behalf of all co-authors)

Reviewer Comment: I found the manuscript difficult to read. It reads somewhat like a how-to guide and somewhat like a software package. I recommend approaching this as a software package, which would require adding evidence to support the choices made. Describe the purpose for the package, evidence for the choices made, benchmarking (compute and performance), describe application to one or more case studies, and discuss how the work fits into the context.

Response: Thank you for the honest feedback on the readability of our paper. We have now made substantial revisions to the Introduction and Discussion sections to describe our aims and conclusions for Omada as a software package.
We want to stress that Omada's objective is not to provide superior clustering algorithms compared to other methods. Instead, we aim to highlight that Omada is a toolkit equipped with various functions, metrics, and algorithms to assess datasets suitability for partitioning to the formation of clusters. It is not just about offering metrics and algorithms for selection; rather, our approach is designed to help both experienced and inexperienced users achieve justifiable clustering outputs. We also provide a number of test datasets (some previously unpublished) for users to explore different parameters and how they perform in datasets from different health conditions and tissues.

Introduction
The rapid development of next-generation sequencing boosted the quantitative analysis of gene expression in a variety of human tissues and organs1,2 generating valuable resources3 for downstream investigative analysis. In recent years, such analyses aim to elucidate disease mechanisms4 and construct genomic profiles5 to explain diagnosis6, prognosis and treatment patterns. However, transcriptomic profiles can be heterogeneous due to several causes pertaining to technical biases that produce batch effects7, cellular diversity8, disease heterogeneity9 as well as differences between individuals and populations10,11. In turn, this heterogeneity hinders research efforts aiming to identify subpopulations within complex diseases by applying unsupervised machine learning techniques12. More specifically, clustering algorithms have been applied to high-dimensional data from transcriptomic profiling13–15, and revealed novel molecular classes associated with different symptoms of disease16–18. Despite the intrinsic capabilities of these algorithms, a recurrent challenge is the insufficiency of default configurations to tailor these models precisely to unique datasets, thereby impeding the extraction of critical insights into the heterogeneity of the samples. This limitation underscores the complexity and heterogeneity inherent in biological data, which often eludes a one-size-fits-all approach to clustering. Empirical evidence from numerous comparisons and tests reveals that the algorithms exhibit variable efficacy across different datasets and disease contexts, accentuating the necessity for model tuning or optimization specific to the dataset in question, particularly with advanced methodologies19,20,21,22. The challenge is further compounded by the possibility that the samples could be from a spectrum of states and there are no optimal metrics to group them.

Powered by Editorial Manager® and ProduXion Manager® from Aries Systems Corporation

Most clustering packages offer implementations of one or more algorithms without considering the previous and subsequent steps of the clustering process, such as detecting useful features or estimating the number of subgroups in the data. Since clustering consists of multiple steps, it is important to carefully approach each step so that intermediate decisions are not arbitrary but based on appropriate clustering theory. Due to this diversity in methodologies, there is a need for a toolkit that guides non-expert users through the different parameters of common clustering algorithms for their unsupervised analysis. One of the most important aspects of sample partitioning is the stability of the generated groups as unstable clusters, usually imply the lack of signal which should be present and drive the clustering of samples. Signals from genes across the transcriptome can vary across different tissues and diseases. Cluster instability can be caused inherently by the data points or by the type and application quality of a clustering technique. There lacks a robust approach that incorporates comprehensive assessment of multiple clustering methods and parameters for different sources of transcriptomic data.
Here we introduce Omada, a toolkit with multiple functions based on cluster stability and quality metrics that supports both experienced and inexperienced users from dataset exploration to the formation of the sample clusters. In contrast to other methodologies, we provided a flexible toolkit offering multiple metrics and algorithms for testing, as well as an end-to-end pipeline. Synthetic and real-world datasets from bulk tumour, single cell sequencing and whole blood have been used to test the general usability of Omada. While we cannot guarantee finding the optimal parameters, especially for healthy test samples where true clusters are unknown, we aim to provide users with guidelines based on quality metrics and empirical best practices, in order to ensure that their clustering results are robust and well justified.

Discussion
Omada is designed to enable the assessment of multiple clustering solutions arising from transcriptomic studies of human samples. With the plurality of unsupervised methods available and their specialised nature, the selection of the most appropriate approach is a multi-factor problem and suggesting a general system of recommendation is impossible without considering each dataset in advance21. Many decisions on technical parameters are required from the user of clustering approaches in order to get a meaningful set of subgroups 71. To assist with this problem, our toolkit initially assesses the potential of a target dataset and provides recommendation for the most appropriate clustering algorithm, gene set and number of possible subgroups. Omada can also be run as a pipeline, therefore, does not require deep prior knowledge of the algorithms, parameters and metrics by the user. All outputs of the pipeline, intermediate and final, are observable and each step is justified by multiple measures and indices representing widely used clustering techniques. We showed that a user of Omada can conduct analysis to assess the feasibility of applying different clustering algorithms and parameters to different RNAseq datasets from healthy and diseased tissue samples.

Conducting unsupervised learning of expression datasets is often not a straightforward task as the true heterogeneity of the health condition or tissue of origin is unknown. No methods or metrics can give a definitive answer without validation from external biological or clinical insights, therefore, the output of each algorithm has to be used with caution. Determining the dataset clustering potential is an indication rather than a clear sign that partitioning the dataset will yield informational subgroups71. Additionally, clustering can often contain non-deterministic steps (i.e. k-means initial cluster centers selection40) allowing for each function to behave slightly differently between similar runs. In order to evaluate the uncertainty and provide a robust set of tools, Omada has been applied to gene expression datasets where we have prior confidence in the number of clusters (simulated and cancer type data) and where we have little prior knowledge (GUSTO whole blood, PAH). Even in data from healthy tissue, such as from GUSTO whole blood, small technical biases can result in prominent clusters detected by Omada. It is also possible for Omada to hint towards the existence of a single homogeneous cluster by consistently revealing low partition agreement and stability scores across multiple functions, as demonstrated in our single-class datasets. Furthermore, Omada can help in selecting a small group of genes with potential partitioning capabilities as the feature selection step is expected to greatly reduce the number of genes which in most cases count to thousands.

Powered by Editorial Manager® and ProduXion Manager® from Aries Systems Corporation

This toolkit currently encompasses common clustering techniques, metrics and test datasets, but its modular nature allows its extension to more specific clustering problems, such as for single cell and spatial data. Furthermore, the structure of this toolkit allows for additional algorithms and metrics to be added to the pool of clustering methods to be evaluated during an analysis run. Systematic empirical testing of algorithms using Omada's approach may result in more robust and better justified clustering outputs for a variety of biomedical studies.
We would also like to highlight benchmarking of compute time and scalability performance which was added in the last revision. In the "Test datasets" section, we added the data processing steps of PBMC single-cell use case.
In the methods section, we added a new section "Evaluation of run time and memory usage". A new section "Scalability Performances" in the result section was also added to report how Omada scales in terms of runtime and memory for simulated datasets of different sizes for both bulk RNA-seq and single-cell data use cases.
In addition to scalability performance, we also performed the benchmarking of Omada against other clustering methods and added the section "Benchmarking Omada against other clustering approaches". Furthermore, benchmarking was also performed on a high-dimensional single-cell use case where a random subset of 4 major celltype including monocytes, B cells and CD4 and CD8 T cells consisting of 1000 cells and 10000 features/genes were analysed.

To compare Omada's results to previous clustering results from cancer studies, we utilise an additional TCGA LUAD dataset from PMID 35664309. With this, we added additional guidelines on the data processing step. We added the results section under "Discriminating cancer types from pan cancer tissue expression data".

Reviewer comment: The evaluation includes two simulation studies and then application to a few real datasets; however, for all real datasets the problem is either very easy or the answer is unknown. The largest challenges I have with the manuscript are the large number of arbitrarily selected parameters the limited evidence available to support those as strong choices.

Response: We understand that real datasets can vary in complexity, ranging from being straightforward to having unknown answers. However, when the cluster memberships are known, such as in the case of two simulated datasets (single-class or multi-class with 5 classes), the Omada pipeline can be automated efficiently, and validation is trivial.
Conversely, in most datasets from human tissue, as seen in our data from pulmonary hypertension and maternal blood, descriptive statistics and parameters from the Omada pipeline need to be outputted and evaluated by the user in order to estimate what might be the true clusters. The user would need prior knowledge about the health conditions and other phenotypic information from the donors.
To provide more evidence on the choices made in our example analyses, we have made the following amendments.
Under "Sample and gene expression preprocessing", we adjusted the following text:
Various normalisations can be utilised, each catering for specific data types (i.e. arcsine transformation fit RNA-seq data, as described in section "Test datasets"). It's worth noting that Omada applies the arcsine transformation on the input data during the first step and the transformed data are carried over to subsequent steps.
In addition, we added data processing guidelines for the PBMC single-cell dataset use case:
Additionally, high-dimensional data such as the single-cell transcriptomic data should be properly quality-controlled where dying (or non-viable) cells and cells with empty droplets or multiplets should be removed. Together with normalisation, scaling, clustering and cell type specific quality control, such data pre-processing steps ensured high quality data to be considered for further analysis.

For stability based analysis, we added the following:

As for the number of genes, it is advised for larger genesets (>1000 genes) to filter down to the most variable ones (few hundreds) before the application of any function as genes that do not vary across samples do not contribute towards identifying heterogeneity. In this step the filtering should not be very strict but rather discard genes with zero or near zero variance (i.e. housekeeping genes). In Omada, the main filtering

is achieved in a later stage by identifying the set of genes that outputs the highest stability of generated clusters, as described in section "Stability-based assessment of feature sets"

On "Determining clustering potential", we added the following text:

At the start of each study, we assess the suitability of the input dataset for clustering, using the Omada package pre-pipeline analysis, to ensure general dataset attributes do not influence the process (Figure 1). The number of samples and features (e.g. genes), as well as the balance of the two dimensions, directly affects the capabilities of clustering methods to handle the dataset. An inadequate number of samples does not provide enough training power44, while an overabundance of samples might clutter the provided information and confuse most methodologies45. Similarly, too few features can lead to weak clustering criteria and too many features might lead a methodology away from the features that can really differentiate between clusters of samples. Therefore, to estimate the feasibility of a clustering procedure on a specifically sized dataset we rely on measurable metrics of cluster quality, such as stability. Clusters of high stability denote both a partitionable dataset as well as a dataset-suitable methodology46. The feasibility score of any dataset is a function of both dimensions as well as the number of classes requested. As such, if too many or a single class is requested of a relatively small dataset the calculation will reflect low feasibility due to insufficient samples and/or features to form the desired classes. Omada's functions feasibilityAnalysis() and feasibility_analysis_based_on_data.R, can simulate datasets with dimensions similar to any dataset and calculate their stability across different numbers of clusters to determine whether such a dataset can be stable enough to be considered for clustering.

Under "Intra-method Clustering Agreement" section, we included

We implemented a tool (Figure 1) to calculate an average agreement score per clustering approach by comparing a number of runs within each of the three clustering approaches (hierarchical47, k-means48, spectral clustering49) using multiple parameters (kernels, measures, algorithms) specifically based on the data set provided which is already arcsine transformed from the first pipeline step. The aforementioned clustering methods were selected to represent fundamentally different approaches of partitioning data so as to cover several types of datasets. Hierarchical clustering is well suited for hierarchical data or data that can be represented as dendrograms, such as taxonomies (Rodriguez et al. 2019). K-means allows a different approach of partitioning samples, namely Centroid-based clustering, which is based on non-hierarchical clusters, vector quantization and representing clusters with their centroids (Rodriguez et al. 2019). Spectral clustering adds a spatial component to k-means as it utilises a similarity matrix to create a graph where clusters with irregular shapes are investigated (Rodriguez et al. 2019). Although the above approaches are not exhaustive, the modular nature of Omada allows for additional approaches to be added in subsequent versions.

We also adjusted the following text to address comparison between runs.

The number of comparisons (c), between runs of the same approach, is an additional overarching parameter of this tool and contributes to the agreement score. Each comparison requires two variations of the same algorithm. These variations are expressed as clustering parameters such as distance measures or in the case of hierarchical clustering linkage type (Table 2). A difference in such parameters produces clustering models that despite using the same algorithm have different formulas to calculate clusters. If these different formulas agree on how to partition the data then the algorithm using them demonstrates that it can capture an underlying signal in the data rather than randomly distributing data points. For each comparison, the parameters of the two runs are drawn randomly from a predefined set (Table 2) selected randomly with replacement while not allowing the same parameters to be used within one comparison. As we have to decide on a dataset without prior training on similar data, the random choice of multiple parameters is more reliable than selecting any single parameter. Additionally, in the interest of performance and computational time we suggest three comparisons to be used instead of exhaustively using every parameter.

Under "spectral clustering algorithm", we added details to the stepwise method.

Given a set of points S = {s1 ... ,sn} in Rl that we want to cluster into k subsets:

Form the affinity matrix A∊ Rn✖n defined by Aij = exp(-||si - sj||2 /2σ2) if i ≠ j , and Aii = 0

Define D to be the diagonal matrix (where A(i,j) = 0) whose (i, i)-element is the sum of A's i-th row, and construct the matrix L = D-l/ 2AD-l/ 2
Find x1 , x2 , ... , xk , the k largest eigenvectors of L (chosen to be orthogonal to each other in the case of repeated eigenvalues), and form the matrix X = [x1 x2 … xk] ∊ n✖k by stacking the eigenvectors in columns
Form the matrix Y from X by renormalizing each of X's rows to have unit length (i.e. Yij = Xij / (Σj X2ij)1/2)
Treating each row of Y as a point in Rk , cluster them into k clusters via K-means or any other algorithm (that attempts to minimize distortion)
Finally, assign the original point si to cluster j if and only if row i of the matrix Y was assigned to cluster j

Under "hierarchical clustering algorithm" we added the following:

Hierarchical clustering algorithm (average linkage)
Given a set of points S = {s1 ... ,sn} that we want to cluster into k subsets:
Initialize with n clusters, each containing one data point (si)
Compute the between-cluster distance D(r, s) as the between-object distance of the two data points in clusters r and s respectively, r, s =1, 2, ..., n. Let the square matrix D = (D(r, s)). Various distances can be used (euclidean, manhattan, canberra, minkowski, maximum).
Find the most similar pair of clusters r and s, such that D(r, s) is minimum among all pairwise distances
Merge r and s to a new cluster t and compute the between-cluster distance D(t, k) for any existing cluster k ≠ r, s. Once the distances are obtained, delete the rows and columns corresponding to the old cluster r and s in the D matrix, as r and s do not exist anymore. Then add a new row and column in D corresponding to cluster t.
Repeat Step 3 a total of n − 1 times until there is only one cluster left (effectively minimising the number of redundant clusters).
Decide on a point to cut the cluster tree created above so as to obtain the desirable number of clusters (k)

In the "Stability-based assessment of feature sets" section, we added
To assess the suitability of each resampled feature set for our clustering, we measure the average stability of the clusters they generate per run when a clustering method is applied over a range of k's (Figure 2B). First, the clustering range, where the stability of each dataset will be calculated, is selected. The lower end of this range is 2, the minimum possible number of clusters and the upper case is selected by the user. For each dataset we generate the bootstrap stability for every k within range (clusterboot function in R package fpc v2.2-3 using nonparametric bootstrap, spectral clustering and 25 resampling runs).

In the "Choosing k number of clusters", we added the following text.

Most clustering methods require the number of k clusters to be defined as a parameter before the application of the algorithm on the data. The lack of a concrete way to determine the real number of clusters in a dataset led many studies to base their estimation on field/prior knowledge or various estimation methods such as the Silhouette score52. However, each method favours different aspects of the generated clusters (i.e. how compact clusters are and how far apart cluster centres are) and therefore suits specific datasets and may introduce bias towards the selection of k. To encompass these different angles in one methodology, avoid the risk of selecting an ineffective index and present a more general solution, this tool uses an ensemble learning approach (Figure 1) where multiple internal cluster indexes contribute to the decision making53. This approach prevents any bias from specific metrics and frees the user from making decisions on any specific metric and assumptions on the optimal number of clusters.

Initially, the value of the 15 indexes is calculated for each k within a cluster range of [2, x], where x is a logical upper limit of the number of clusters realistic for our dataset (i.e. the present conditions within a dataset). The means over k are calculated per index and the optimal k is estimated by majority voting of the 14 means that evaluate the compactness and/or the distance between different subgroups. The selection of

indexes can be found in Table 3. It is important to note that the most important aspect of determining k is minimum loss of information which directs us to overestimate and not underestimate k51 while interpreting the voting results. Furthermore, cases that present only a single k as the optimal number of clusters should be treated with caution in case they are a result of a biased dataset. Omada scores each k within the set range [2, x] enabling the user to observe the most probable number of clusters according to the above criteria (function clusterVoting()). This prediction is agnostic to any other type of biological information except gene expression. However, after the clustering groups are formed it is advised for the user to investigate whether they represent relevant to the specific case biological subgroups."

Reviewer Comment: Conceptually, an alternative strategy is to consider real clusters to be those that are robust over many clustering methods. In this case, the best clusters are those that are maximally detectable with a single method. While there exists software for the former strategy, this package implements the latter strategy. It is not intuitively clear to me that this framework is superior to the other for biological discovery. It seems like general clusters (i.e., those that persist across multiple parameterizations) may be the most fruitful to pursue. It would be helpful to provide evidence that the selected strategy has superior utility in at least some settings and a description of how those settings might be identified. It is possible this is not necessary, but I simply note it as I continue to have these challenges with the revised manuscript.

Response: We agree that examining multiple clustering outputs and finding their agreement could be an effective strategy towards finding true cluster memberships. Since there is no standard way of aggregating multiple clustering memberships and reaching a consensus that guarantees the most robust clusters, we left it to the users to subjectively decide. Users of Omada are able to stop at each step of the pipeline and compare parameters, but we have now added functions for them to get the partition agreement between different clustering outputs and parameters tested.

We have also added the option for users to conduct consensus clustering using M3C to find the clusters that persist across multiple parameterizations, as you have suggested. We caution them to still use human judgement by showing an example of clustering cancer types where there may not be a single clustering run that generates the most likely number of clusters.

Methods

Exploring the number of clusters (k) when using single algorithms versus consensus clustering

In this section we test whether, in RNA-seq clustering tasks, a single clustering algorithm can be a superior choice than other algorithms which represent different approaches. Towards this goal we extract gene expression datasets from the Cancer Genomics cBioPortal (https://www.cbioportal.org/) and test the extent to which different algorithms can recognise different cancer types. The first dataset consists of three cancer types: Esophageal Adenocarcinoma (esca, n = 181) , Thymoma (thym, n = 119) and Testicular Germ Cell Tumors (tgct, n = 149). The 20,531 genes were first filtered to exclude duplicates as well as those without expression values for the majority of samples. They were subsequently ranked, in a descending manner, for variance across samples and then 500 most variant genes were included in the clustering models. The dataset was normalised using the inverse hyperbolic sine (function asinh in base R package). The second dataset was simulated based on exact dimensions of the aforementioned cancer dataset using Omada's simulation function feasibilityAnalysisDataBased() with 3 distinct classes. Spectral, kmeans and hierarchical clustering algorithms were tested alongside a consensus clustering method M3C from package https://www.bioconductor.org/packages/release/bioc/html/M3C.html. Default parameters were used for each algorithm. The adjusted Rand index was calculated between the set of generated memberships of the top k estimate and the true cancer type labels of the samples. Additionally, we registered the k estimation of each methodology against the real number of different cancer types for each dataset. To

Powered by Editorial Manager® and ProduXion Manager® from Aries Systems Corporation

estimate k for hierarchical, spectral and k-means the Omada ensemble method was used, while for M3C the provided Relative Cluster Stability Index (RCSI).

Results

Not all algorithms can detect the true number of clusters

We tested the ability of four algorithms to cluster samples that belong to three cancer types. As seen on Table X1, hierarchical clustering was not able to identify the three cancer types (ARI = 0.55) as it selected two clusters as most probable. On the contrary, spectral clustering separated the samples most accurately (ARI = 0.83) with a clear preference for three clusters (8/14 votes) closely followed by k-means (ARI = 0.80 and 5/14 votes). Consensus clustering followed with an ARI of 0.78 but a widest spread of estimated k as it selected 5 and 6 clusters as second and third choices respectively. On the simulated dataset (Table X2), k-means estimated the correct number of clusters and managed to assign the memberships perfectly (ARI = 1). All other algorithms selected a different k and therefore presented a much lower ARI (as comparing a set of two clusters with a set of three clusters cannot result in a high ARI). Both Omada and consensus clustering M3C suggest k = 3 as the second estimate. However, Omada already supplies the memberships for k = 3 as part of the k estimation achieving a perfect ARI of 1 if that partitioning is selected for all three algorithms.

Table 4: The ARI of the top k estimate of various datasets with different numbers of cancer types

Algorithm	Real clusters		
	3	5	7
Hierarchical	0.55 (k=2)	0.13 (k=2)	0.19 (k=2)
k-means	0.80 (k=3)	0.54 (k=4)	0.45 (k=)
Spectral	0.83 (k=3)	0.84 (k=5)	0.58 (k=)
M3C	0.78 (k=3)	0.85 (k=5)	0.63 (k=9)

Table 5: The ARI of the top k estimate of various datasets with different numbers of simulated classes

Algorithm	Real clusters		
	3	5	7
Hierarchical	0.57 (k=2)	0.9 (k=7)	0.9 (k=9)

Powered by Editorial Manager® and ProduXion Manager® from Aries Systems Corporation

	k-means<br>1.00 (k=3)<br>1.00 (k=5)<br>0.79 (k=8)<br>Spectral<br>0.57 (k=2)<br>1.00 (k=5)<br>1.00 (k=7)<br>M3C<br>0.57 (k=2)<br>0.78 (k=5)<br>0.54 (k=4)
**Additional Information:**	
**Question**	**Response**
Are you submitting this manuscript to a special series or article collection?	No
**Experimental design and statistics**<br><br>Full details of the experimental design and statistical methods used should be given in the Methods section, as detailed in our Minimum Standards Reporting Checklist. Information essential to interpreting the data presented should be made available in the figure legends.<br><br>Have you included all the information requested in your manuscript?	Yes
**Resources**<br><br>A description of all resources used, including antibodies, cell lines, animals and software tools, with enough information to allow them to be uniquely identified, should be included in the Methods section. Authors are strongly encouraged to cite Research Resource Identifiers (RRIDs) for antibodies, model organisms and tools, where possible.<br><br>Have you included the information requested as detailed in our Minimum Standards Reporting Checklist?	Yes
**Availability of data and materials**	Yes

All datasets and code on which the conclusions of the paper rely must be either included in your submission or deposited in publicly available repositories (where available and ethically appropriate), referencing such data using a unique identifier in the references and in the "Availability of Data and Materials" section of your manuscript.<br><br>Have you have met the above requirement as detailed in our Minimum Standards Reporting Checklist?	

Manuscript

Click here to access/download;Manuscript;Clean_Omada Manuscript V3.pdf

Omada: Robust clustering of transcriptomes through multiple testing

Sokratis Kariotis[1,2,4], Tan Pei Fang[1,2], Haiping Lu[3] , Christopher J Rhodes[4], Martin R Wilkins[4], Allan Lawrie[4], Dennis Wang[1,2,3,4]#

[1] Singapore Institute for Clinical Sciences, Agency for Science, Technology and Research (A*STAR), Singapore, Republic of Singapore.

[2] Bioinformatics Institute, Agency for Science, Technology and Research (A*STAR), Singapore, Republic of Singapore

[3] Department of Computer Science, University of Sheffield, Sheffield UK.

[4] National Heart and Lung Institute, Imperial College London, London, UK

Corresponding authors: Sokratis Kariotis, Dennis Wang (dennis.wang@imperial.ac.uk)

Abstract

Cohort studies increasingly collect biosamples for molecular profiling and are observing molecular heterogeneity. High throughput RNA sequencing is providing large datasets capable of reflecting disease mechanisms. Clustering approaches have produced a number of tools to help dissect complex heterogeneous datasets, however, selecting the appropriate method and parameters to perform exploratory clustering analysis of transcriptomic data requires deep understanding of machine learning and extensive computational experimentation. Tools that assist with such decisions without prior field knowledge are nonexistent. To address this we have developed Omada, a suite of tools aiming to automate these processes and make robust unsupervised clustering of transcriptomic data more accessible through automated machine learning based functions. The efficiency of each tool was tested with seven datasets characterised by different expression signal strengths to capture a wide spectrum of RNA expression datasets. Our toolkit's decisions reflected the real number of stable partitions in datasets where the subgroups are discernible. Within datasets with less clear biological distinctions, our tools either formed stable subgroups with different expression profiles and robust clinical associations or revealed signs of problematic data such as biased measurements. Implementation of Omada is available at https://bioconductor.org/packages/omada.

Introduction

The rapid development of next-generation sequencing boosted the quantitative analysis of gene expression in a variety of human tissues and organs[1,2] generating valuable resources[3] for downstream investigative analysis. In recent years, such analyses aim to elucidate disease mechanisms[4] and construct genomic profiles[5] to explain diagnosis[6], prognosis and treatment patterns. However, transcriptomic profiles can be heterogeneous due to several causes pertaining to technical biases that produce batch effects[7], cellular diversity[8], disease heterogeneity[9] as well as differences between individuals and populations[10,11]. In turn, this heterogeneity hinders research efforts aiming to identify subpopulations within complex diseases by applying unsupervised machine learning techniques[12]. More specifically, clustering algorithms have been applied to high-dimensional data from transcriptomic profiling[13–15], and revealed novel molecular classes associated with different symptoms of disease[16–18]. Despite the intrinsic capabilities of these algorithms, a recurrent challenge is the insufficiency of default configurations to tailor these models precisely to unique datasets, thereby impeding the extraction of critical insights into the heterogeneity of the samples. This limitation underscores the complexity and heterogeneity inherent in biological data, which often eludes a one-size-fits-all approach to clustering. Empirical evidence from numerous comparisons and tests reveals that the algorithms exhibit variable efficacy across different datasets and disease contexts, accentuating the necessity for model tuning or optimization specific to the dataset in question, particularly with advanced methodologies[19,20,21,22]. The challenge is further compounded by the possibility that the samples could be from a spectrum of states and there are no optimal metrics to group them.

Most clustering packages offer implementations of one or more algorithms without considering the previous and subsequent steps of the clustering process, such as detecting useful features or estimating the number of subgroups in the data. Since clustering consists of multiple steps, it is important to carefully approach each step so that intermediate decisions are not arbitrary but based on appropriate clustering theory. Due to this diversity in methodologies, there is a need for a toolkit that guides non-expert users through the different parameters of common clustering algorithms for their unsupervised analysis. One of the most important aspects of sample partitioning is the stability of the generated groups as unstable clusters, usually imply the lack of signal which should be present and drive the clustering of samples. Signals from genes across the transcriptome can vary across different tissues and diseases. Cluster instability can be caused inherently by the data points or by the type and application quality of a clustering technique. There lacks a robust approach that

incorporates comprehensive assessment of multiple clustering methods and parameters for different sources of transcriptomic data.

Here we introduce Omada, a toolkit with multiple functions based on cluster stability and quality metrics that supports both experienced and inexperienced users from dataset exploration to the formation of the sample clusters. In contrast to other methodologies, we provided a flexible toolkit offering multiple metrics and algorithms for testing, as well as an end-to-end pipeline. Synthetic and real-world datasets from bulk tumour, single cell sequencing and whole blood have been used to test the general usability of Omada. While we cannot guarantee finding the optimal parameters, especially for healthy test samples where true clusters are unknown, we aim to provide users with guidelines based on quality metrics and empirical best practices, in order to ensure that their clustering results are robust and well justified.

Methods

A number of clustering methodologies are shown in Table 1 along with the steps of the clustering process they address.

Table 1: Comparison of clustering methodologies towards identifying heterogeneous patient groups. Five steps of the clustering process are reflected in the columns below. Feasibility analysis assesses the potential of the dataset to be utilised efficiently by clustering methods based on its dimensions. The clustering algorithm denotes the amount of algorithms available in the methodology. Feature selection allows the selection of the features that best discriminate samples during clustering.. Cluster quality describes the methods/metrics used to validate the quality of the clusters produced. Number of clusters describes the approach a methodology follows to estimate the most probable number of different groups in the dataset.

	**Feasibility**	**Clustering algorithm**	**Feature selection**	**Cluster quality**	**Number of clusters**
**omada**	Stability based	Multiple	Stability based	internal indexes	multiple index voting
**pvclust** [23]	-	Single	-	bootstrap probability	bootstrap probability
**genieclust** [24]	-	Single	-	internal indexes	-
**ClusterR**[25]	-	Multiple	-	single external index	single index
**pdfCluster** [26]	-	Multiple	-	internal indexes	-
**clusterSim** [27]	-	Single	-	internal indexes	single index
**clustvarsel** [28]	-	-	Gaussian based	-	-
**VarSelLCM** [29]	-	Multiple	BIC/MICL/AIC	-	BIC/MICL/AIC

**FCPS** [30]	-	Multiple	-	internal indexes	single index

This toolkit consists of a pipeline that takes in a gene expression matrix to identify transcriptomic subgroups of samples (Figure 1 and Supplementary Figure 1). Starting from a matrix of gene expression values (e.g. transcripts per million from RNAsequencing), the most suitable clustering method is chosen, followed by selecting the transcript features for clustering and determining the number of clusters and memberships.

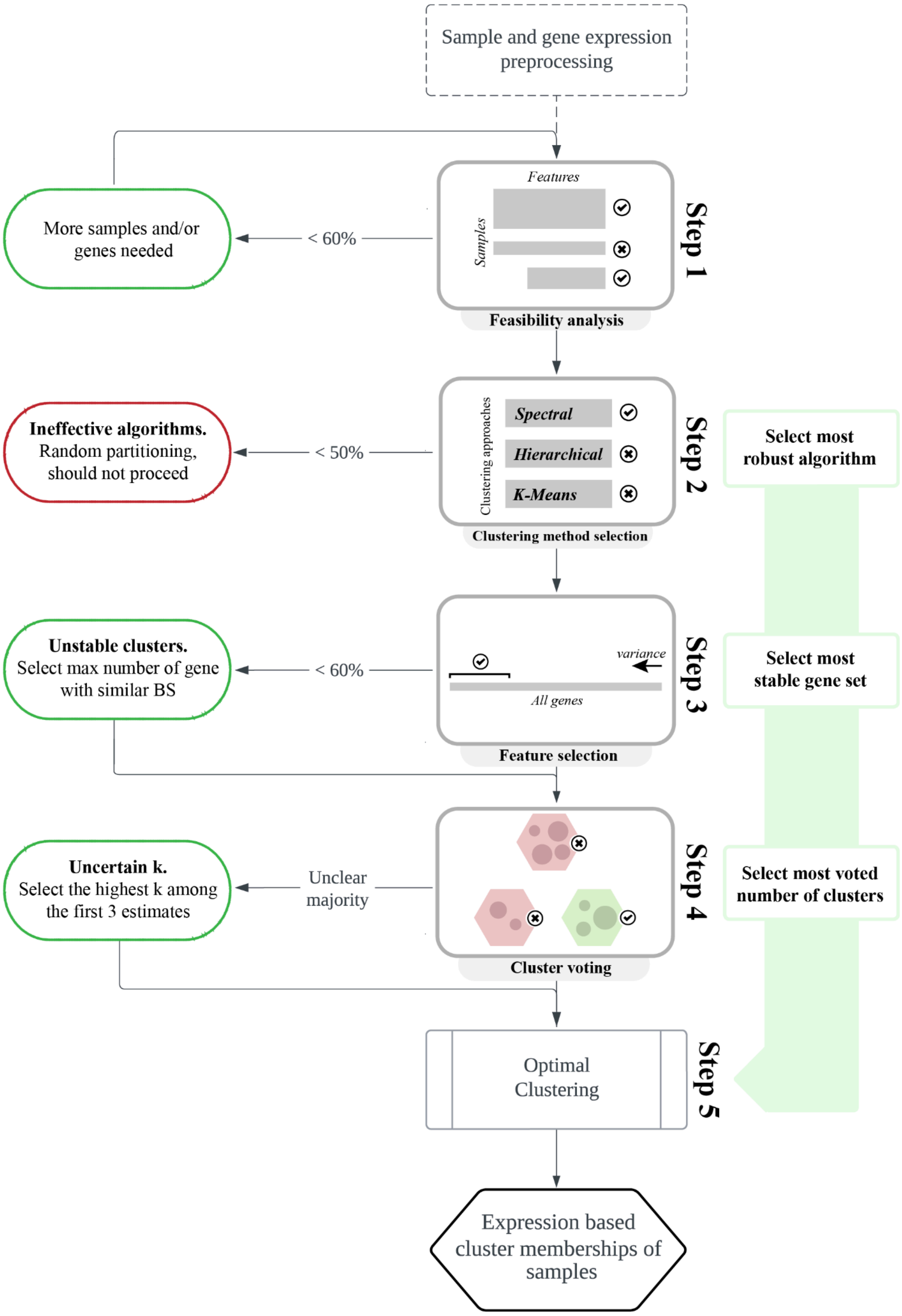

Figure 1: An overview of steps for discovering gene expression subgroups using Omada's clustering tools. Processing and quality control should precede the application of Omada to ensure more accurate inputs. Step 1 of Omada allows a feasibility analysis to ensure that the input data is suitable for clustering. Step 2 selects the clustering methodology that provides the most consistent partitions for downstream analysis. In step 3, the genes that best discriminate samples and yield the most stable clusters are determined. Step 4 estimates the number of clusters (k) through majority voting by internal machine learning indexes. The final output of step 5 provides a cluster assignment to each sample driven solely by its expression profile. Percentage thresholds are recommended values that can be modified.

Sample and gene expression preprocessing

A preprocessing step is recommended by the user before the application of these tools on any dataset to heighten the chances of any underlying important signal to be discovered. Data biases and format can often drive clustering attempts to focus on discriminating data points based solely, or mostly, on known information producing no new insights irrespectively of the method used[21,31]. To address this, it is recommended to attempt to remove/normalise any data points that might be introducing strong biases to allow the novel signal to be detected. Furthermore, numerical data may need to be normalised in order to account for potential misdirecting quantities (i.e. outliers) or specifically transformed to satisfy an algorithm's input criteria. Various normalisations can be utilised, each catering for specific data types (i.e. arcsine transformation fit RNA-seq data, as described in section “Test datasets”). It's worth noting that Omada applies the arcsine transformation on the input data during the first step and the transformed data are carried over to subsequent steps. Data points or samples have to be filtered based on field knowledge to allow the data to answer specific scientific questions. Expression data should go through proper quality control depending on the manner of collection to identify outliers and remove unreliable data points. For microarrays it is important to assess sample, hybridization and overall signal qualities along with signal comparability and potential biases[32]. Array correlations through PCA and correlation plots should also be considered[32]. RNA sequencing experiments also produce data that need to be controlled for potential trimming of adapter sequences, low quality reads, uncalled based and contaminants by using a plethora of available tools[33,34]. qPCR generated data should be checked for abnormal amplification, positive and negative control samples, control on PCR replicate variation and determine reference gene expression stability and deviating sample normalisation factors[35]. Additionally, high-dimensional data such as the single-cell transcriptomic data should be properly quality-controlled where dying (or non-viable) cells and cells with empty droplets or multiplets should be removed. Together with normalisation, scaling, clustering and cell type specific quality control, such data pre-processing steps ensured high quality data to be considered for further analysis. "As for the number of genes, it is advised for larger genesets (>1000 genes) to filter down to the most variable ones (few hundreds) before the application of any function as genes that do not vary across samples do not contribute towards identifying heterogeneity. In this step the filtering should not be very strict but rather discard genes with zero or near zero variance (i.e. housekeeping genes). In Omada, the main filtering is achieved in a later stage by identifying the set of genes that outputs the highest stability of generated clusters, as described in section “Stability-based assessment of feature sets” Moreover, large genesets require increased computational power and extended runtime without adding any real value due to the large number

of non useful genes. Lastly, it is important to note that technical artefacts, such as sampling location or machine specifications, may drive clustering causing the formation of very distinct clusters which can solely be attributed to relevant biases. It is very important for those cases to be identified and extracted insights should be disregarded as they do not reflect real signals or data trends.

Determining clustering potential

At the start of each study, we assess the suitability of the input dataset for clustering, using the Omada package pre-pipeline analysis, to ensure general dataset attributes do not influence the process (Figure 1). The number of samples and features (e.g. genes), as well as the balance of the two dimensions, directly affects the capabilities of clustering methods to handle the dataset. An inadequate number of samples does not provide enough training power[36], while an overabundance of samples might clutter the provided information and confuse most methodologies[37]. Similarly, too few features can lead to weak clustering criteria and too many features might lead a methodology away from the features that can really differentiate between clusters of samples. Therefore, to estimate the feasibility of a clustering procedure on a specifically sized dataset we rely on measurable metrics of cluster quality, such as stability. Clusters of high stability denote both a partitionable dataset as well as a dataset-suitable methodology[38]. The feasibility score of any dataset is a function of both dimensions as well as the number of classes requested. As such, if too many or a single class is requested of a relatively small dataset the calculation will reflect low feasibility due to insufficient samples and/or features to form the desired classes. Omada's functions feasibilityAnalysis() and feasibility_analysis_based_on_data.R, can simulate datasets with dimensions similar to any dataset and calculate their stability across different numbers of clusters to determine whether such a dataset can be stable enough to be considered for clustering.

Simulating datasets

To assess the quality of the dataset to be used, our toolkit includes two functions for simulating datasets of different dimensionalities for stability assessment. We use those to understand the relation between the number of samples, genes and cluster sizes. The first function simulates datasets allowing for selecting the number of samples (n), genes (m) and clusters (c). Each cluster contains $\frac{n}{c}$ samples drawn from a normal distribution with a different mean and standard deviation. Each mean is drawn from a sequence of c evenly spaced integers that belong to the range $[5\ ,\ c\ *\ 10]$. Each standard deviation is similarly drawn from a range of $[1\ ,\ c\ *\ 2]$. To estimate the difference between distributions, we calculate the two sided Kolmogorov's D statistic between each

pair of distributions representing the generated classes and plot the empirical cumulative distribution function (EDCF).

Subsequently, we calculate the stability of each k (number of clusters for a particular clustering run) using the clusterboot function in R package fpc v2.2-3. The number of clusters k to be considered belong to $k \in [number\ of\ classes\ -\ 2,\ number\ of\ classes\ +\ 2]$, with a minimum of $k = 2$. The maximum and average stabilities over all k are reported, providing a stability-based quality score that provides an insight in deciding whether a prospect dataset is suitable for a clustering study.

To assess the clustering feasibility of an existing dataset this tool kit also provides a similar function which generates a simulated dataset based on an input dataset and the user's estimation of the number of classes. The number of samples and genes equal those of the input dataset and its mean (m_{input}) and standard deviation (sd_{input}) affect those of each generated class within the dataset. Specifically, if $n \in (1, 2, 3, \ldots)$ is the number of classes, each class mean (m_{class}) equals $m_{input} * 10 * n$ and each class standard deviation (sd_{class}) equals $sd_{input} * 2 * n$.

Intra-method Clustering Agreement

Unsupervised learning offers a multitude of methods to be applied on specific types of data due to their nature (e.g. numeric, binary) or underlying signal to be detected. Most studies employ widely-used methods (e.g. hierarchical clustering) without exercising any kind of selection method that would point towards the most effective methodology. Selecting an appropriate approach requires extensive machine learning and data analysis knowledge coupled with tuning and testing of multiple different algorithms. To enable users without ML expertise to utilise the vast capabilities of this field and avoid limited efficiency of default methodologies, we present a clustering selection tool that offers an intelligent selection method with unbiased results through parameter randomization. The nature of this selection method allows any number of well established unsupervised methods to be considered.

To address the lack of class labels and thus a performance measure in unsupervised models, we compare how consistently different approaches partition our data when one or more parameters change. As high consistency we define the high agreement score calculated between different variations of a clustering algorithm. When two different clustering runs agree on the partitioning of

the samples they also show robustness since they do not randomly assign samples to subgroups but rather are driven by the underlying structure of the data.

We implemented a tool (Figure 1) to calculate an average agreement score per clustering approach by comparing a number of runs within each of the three clustering approaches (hierarchical[39], k-means[40], spectral clustering[41]) using multiple parameters (kernels, measures, algorithms) specifically based on the data set provided which is already arcsine transformed from the first pipeline step. The aforementioned clustering methods were selected to represent fundamentally different approaches of partitioning data so as to cover several types of datasets. Hierarchical clustering is well suited for hierarchical data or data that can be represented as dendrograms, such as taxonomies[42]. K-means allows a different approach of partitioning samples, namely Centroid-based clustering, which is based on non-hierarchical clusters, vector quantization and representing clusters with their centroids[42]. Spectral clustering adds a spatial component to k-means as it utilises a similarity matrix to create a graph where clusters with irregular shapes are investigated[42]. Although the above approaches are not exhaustive, the modular nature of Omada allows for additional approaches to be added in subsequent versions.

The number of comparisons (*c*), between runs of the same approach, is an additional overarching parameter of this tool and contributes to the agreement score. Each comparison requires two variations of the same algorithm. These variations are expressed as clustering parameters such as distance measures or in the case of hierarchical clustering linkage type (Table 2). A difference in such parameters produces clustering models that despite using the same algorithm have different formulas to calculate clusters. If these different formulas agree on how to partition the data then the algorithm using them demonstrates that it can capture an underlying signal in the data rather than randomly distributing data points. For each comparison, the parameters of the two runs are drawn from a predefined set (Table 2) selected randomly with replacement while not allowing the same parameters to be used within one comparison. As we have to decide on a dataset without prior training on similar data, the choice of multiple parameters is more reliable than selecting any single parameter. Additionally, in the interest of performance and computational time we suggest three comparisons to be used instead of exhaustively using every parameter. Depending on *c*, we generate variations of the base clustering algorithms (package kernlab v0.9-29), along with the various distance measures and clustering categories they belong to. Within each pair of clustering runs the agreement is calculated using the adjusted Rand Index (package fossil v0.3.7), the corrected-for-chance version of the original Rand index[43], which is based on the number of times any pair of points is partitioned in the same subgroup throughout different clusterings runs. To

calculate the agreement within each clustering algorithm (spectral, k-means, hierarchical) we are considering pairs of runs using the same algorithm but different parameters. For those pairs the agreement is averaged across clustering runs and k number of clusters tested. The algorithm that presents the highest intra-method agreement over a logical range of clusters ($k \in [2, x]$) is noted as the most appropriate clustering of the samples based on a detected signal. A logical range of k is considered a set of successive k's (where $k \geqslant 2$) that is most probable to exist within our data, often determined by prior knowledge of the data, previous studies or domain expertise. This selection procedure is mainly affected by the type and size of the data leading similar datasets to opt for the same method due to the specific mathematical formulas within each algorithm.

Spectral clustering algorithm[41]

Given a set of points S = $\{s_1 \ldots ,s_n\}$ in R^l that we want to cluster into **k** subsets:

1. *Form the affinity matrix* $A \in Rn ✖ n$ *defined by* $Aij = exp(-||si - sj||2 /2\sigma 2)$ *if* $i \neq j$ *, and* $Aii = 0$
2. *Define D to be the diagonal matrix (where A(i,j) = 0) whose (i, i)-element is the sum of A's i-th row, and construct the matrix* $L = D^{-1/2}AD^{-1/2}$
3. *Find* $x_1 , x_2 , \ldots , x_k$ *, the k largest eigenvectors of L (chosen to be orthogonal to each other in the case of repeated eigenvalues), and form the matrix* $X = [x_1\ x_2\ \ldots\ x_k] \in {}^{n✖k}$ *by stacking the eigenvectors in columns*
4. *Form the matrix Y from X by renormalizing each of X's rows to have unit length (i.e.* $Y_{ij} = X_{ij} / (\Sigma_j X^2_{ij})^{1/2}$*)*
5. *Treating each row of Y as a point in* R^k *, cluster them into k clusters via K-means or any other algorithm (that attempts to minimize distortion)*
6. *Finally, assign the original point* s_i *to cluster j if and only if row i of the matrix Y was assigned to cluster j*

Hierarchical clustering algorithm (average linkage)

Given a set of points S = $\{s_1 \ldots ,s_n\}$ that we want to cluster into **k** subsets:

1. *Initialize with n clusters, each containing one data point* (s_i)

---

2. *Compute the between-cluster distance D(r, s) as the between-object distance of the two data points in clusters r and s respectively, r, s =1, 2, ..., n. Let the square matrix D = (D(r, s)). Various distances can be used (euclidean, manhattan, canberra, minkowski, maximum).*
3. *Find the most similar pair of clusters r and s, such that D(r, s) is minimum among all pairwise distances*
4. *Merge r and s to a new cluster t and compute the between-cluster distance D(t, k) for any existing cluster k ≠ r, s. Once the distances are obtained, delete the rows and columns corresponding to the old cluster r and s in the D matrix, as r and s do not exist anymore. Then add a new row and column in D corresponding to cluster t.*
5. *Repeat Step 3 a total of n − 1 times until there is only one cluster left (effectively minimising the number of redundant clusters).*
6. *Decide on a point to cut the cluster tree created above so as to obtain the desirable number of clusters (k)*

K-means[40]

K: kernel matrix, k: number of clusters, w: weights for each point, tmax: optional maximum number of iterations, $\{\pi_c^{(0)}\}^k_{c=1}$: optional initial clusters

1. *If no initial clustering is given, initialize the k clusters* $\pi_1^{(0)}, \ldots, \pi_k^{(0)}$ *(i.e. randomly). Set t = 0*
2. *For each* a_i *and every cluster c, compute*

$$d(a_i, m_c) = K_{ii} - \frac{2\Sigma_{\alpha_j \in \pi_c^{(t)}} w_j K_{ij}}{\Sigma_{\alpha_j \in \pi_c^{(t)}} w_j} + \frac{\Sigma_{\alpha_j, \alpha_l \in \pi_c^{(t)}} w_j w_l K_{jl}}{(\Sigma_{\alpha_j \in \pi_c^{(t)}} w_j)^2}$$

3. *Find* $c^*(a_i) = \mathrm{argmin}_c d(a_i, m_c)$*, resolving ties arbitrarily. Compute the updated clusters as*

$$\pi_c^{(t+1)} = \{a: c^*(a_i) = c\}$$

4. *If not converged or* $t_{max} > t$*, set t = t + 1 and go to Step 2; Otherwise, stop and output final clusters*

$$\{\pi_c^{(t+1)}\}^k_{c=1}$$

Table 2 | The clustering algorithms, their approach category and the various distance measures tested

Clustering algorithms	Category	Distance measures/kernels	Additional parameters
K-means	Partitioning	Hartigan-Wong, Lloyd, Forgy, MacQueen	-
Hierarchical	Hierarchical	Euclidean, Manhattan, Minkowski, Canberra	Average, complete, median (linkage)
Spectral	Graph Theory	Rbfdot, Polydot, Tanhdot, Laplacedot, Vanilladot, Anovadot, Splinedot	-

Feature set subsampling

While gene expression data provide measures on the thousands of transcripts in the transcriptome, not all of them may provide discriminative information on the samples and may not be useful for clustering. Moreover, most clustering algorithms are heavily affected by a large number of features both computationally due to input size and in performance due to misdirecting data noise[42]. A common strategy to select interesting and potentially useful RNA features is to measure their variance across samples and select the ones with the highest scores instead of those that are either housekeeping or do not differentiate in our context. In this tool, we exclude RNA features that remain stable across samples and are therefore unable to offer any discriminatory power to our unsupervised machine learning models. Furthermore, the exhaustive feature selection procedure incrementally considers all the genes in the feature set and takes into account the stability of all generated test clusters and number of cluster ranges. This step does not require any deep knowledge or filtering decisions by the user.

Based on this observation our sample selection step, which is a part of the tool for bootstrap resampling of features presented in Figures 1 and 2A, first ranks features in a descending order of variance (var function from the Stats R package) across samples, generating a list of the most variable features. Subsequently, multiple datasets of all samples and subsets of features are generated. All subsets draw a different number of features from the top of the variance list with replacement. The first dataset uses a relatively small number of features (n), depending on the total number of features (N) and the granularity of the result desired. The following datasets re-draw from the initial list increasing the number of features by n, ending up with $\frac{N}{n}$ datasets.

Stability-based assessment of feature sets

To assess the suitability of each resampled feature set for our clustering, we measure the average stability of the clusters they generate per run when a clustering method is applied over a range of *k*'s (Figure 2B). First, the clustering range, where the stability of each dataset will be calculated, is selected. The lower end of this range is 2, the minimum possible number of clusters and the upper case is selected by the user. For each dataset we generate the bootstrap stability for every *k* within range (clusterboot function in R package fpc v2.2-3 using nonparametric bootstrap, spectral clustering and 25 resampling runs). To calculate each bootstrap stability score the data is randomly sampled with replacement and clustered internally using a spectral approach. We then compute the Jaccard similarities between the original clusters and the most similar clusters in the resampled data. The above procedure results in a stability score for each *k* and each dataset. We then calculate the final stability of each dataset by averaging the stability over *k*. The genes that comprise the single dataset with the highest stability are the ones that compose the most appropriate set for the downstream analysis.

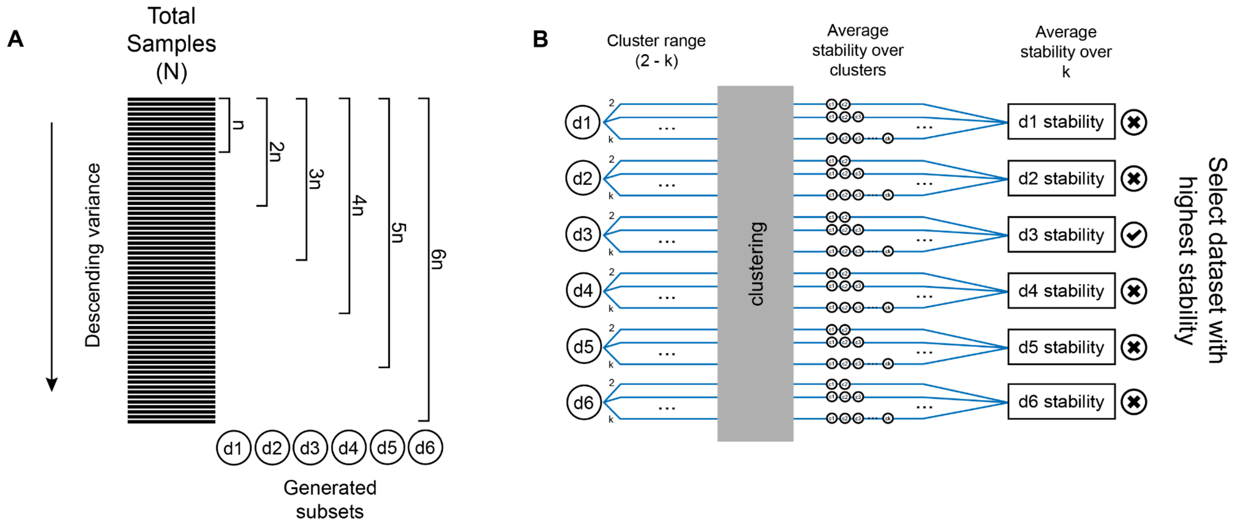

Figure 2: Sample selection overview. (A) Ranking of samples based on their variance across features and the subsequent generation of datasets of increasing size. (B) Calculation of the stability score of each generated dataset. Initially, we select a cluster range to run our clustering method for each dataset. After the clustering procedure, we calculate and average the stability over the generated clusters. Finally, we

average the stabilities over k per dataset and determine a final stability score for each dataset. The features of the dataset with the highest stability are the ones that compose the most appropriate set for the downstream pipeline

Choosing k number of clusters

Most clustering methods require the number of k clusters to be defined as a parameter before the application of the algorithm on the data. The lack of a concrete way to determine the real number of clusters in a dataset led many studies to base their estimation on field/prior knowledge or various estimation methods such as the Silhouette score[44]. However, each method favours different aspects of the generated clusters (i.e. how compact clusters are and how far apart cluster centres are) and therefore suits specific datasets and may introduce bias towards the selection of k. To encompass these different angles in one methodology, avoid the risk of selecting an ineffective index and present a more general solution, this tool uses an ensemble learning approach (Figure 1) where multiple internal cluster indexes contribute to the decision making[45]. This approach prevents any bias from specific metrics and frees the user from making decisions on any specific metric and assumptions on the optimal number of clusters.

Initially, the value of the 15 indexes is calculated for each k within a cluster range of $[2, x]$, where x is a logical upper limit of the number of clusters realistic for our dataset (i.e. the present conditions within a dataset). The means over k are calculated per index and the optimal k is estimated by majority voting of the 14 means that evaluate the compactness and/or the distance between different subgroups. The selection of indexes can be found in Table 3. It is important to note that the most important aspect of determining k is minimum loss of information which directs us to overestimate and not underestimate k[42] while interpreting the voting results. Furthermore, cases that present only a single k as the optimal number of clusters should be treated with caution in case they are a result of a biased dataset. Omada scores each k within the set range [2, x] enabling the user to observe the most probable number of clusters according to the above criteria (function clusterVoting()). This prediction is agnostic to any other type of biological information except gene expression. However, after the clustering groups are formed it is advised for the user to investigate whether they represent relevant to the specific case biological subgroups.

Table 3: The list of 15 internal indexes used to estimate the optimal number of clusters (k). All indexes are using different formulas to score a partitioning, measuring one or both of the following concepts: a) how

compact each cluster is and b) how well the clusters separate. For each index we present which value is preferred (min or max) and its source. For the formulas: k = number of clusters, n = number of data points, E^T = sum of the distances of all the points to the barycenter G of the entire dataset, E^W = sum of the distances of the points of each cluster to their barycenter, N_B = pairs constituted of points which do not belong to the same cluster, N_B = pairs constituted of points which belong to the same cluster, $N_T = N_W + N_B$, S_W = sum of the N_W distances between all the pairs of points inside each cluster, S_{MIN} = sum of the N_W smallest distances between all the pairs of points in the entire data set, S_{MAX} = sum of the N_W largest distances between all the pairs of points in the entire data set, S_B = sum of the between-cluster distances, α = weight equal to the value of average scattering of clusters obtained for the partition with the greatest number of clusters.

**Internal index**	**Ideal**	**Formula**	**Source**
Calinski-Harabasz	max	$\frac{(\frac{between\ cluster\ variation}{k-1})}{(\frac{within\ cluster\ variation}{n-k})}$	46
Dunn	max	$\frac{min(intercluster\ distance)}{max(\ distance\ between\ all\ pairs)}$	47
Pbm	max	$\frac{1}{k} * \frac{E^T}{E^W} * D_B$	48
Tau	max	$\frac{concordant\ pairs - discordant\ pairs}{\sqrt{N_B * N_W \frac{N_T(N_T-1)}{2}}}$	49
Gamma	max	$\frac{concordant\ pairs - discordant\ pairs}{concordant\ pairs + discordant\ pairs}$	50
C index	min	$\frac{S_W - S_{MIN}}{S_{MAX} - S_{MIN}}$	51
Davies–Bouldin	min	$\frac{1}{n} * \sum_{i=1}^{n} max(\frac{clusters\ scatter\ difference}{cluster\ seperation})$	52
Mcclain-Rao	min	$\frac{N_B}{N_W} * \frac{S_W}{S_B}$	53
sd_dis	min	$a * (avg\ scattering\ for\ clusters) +$ $total\ separation\ between\ clusters$	54
Ray–Turi	min	$\frac{1}{n} * \frac{within-cluster\ dispersion}{min\ of\ the\ sq.\ distances\ between\ all\ the\ cluster\ barycenters}$	55
g_plus	min	$\frac{2 * s^{-}}{N_T(N_T - 1)}$	56

Silhouette	max	$average\ distance\ between\ clusters$	44
s_dbw	min	$mean\ dispersion\ of\ clusters\ +\ between\ cluster\ density$	57
Compactness	max	$Intra - Cluster\ distance$	58
Connectivity	max	$The\ extent\ by\ which\ the\ items\ are\ placed\ in\ the$ $same\ cluster\ as\ their\ nearest\ neighbours\ in\ the\ data\ space$	-

Optimal parameter tuning

Previous steps have selected the optimal method, number of features and clusters. To perform the optimal clustering we automate the selection of parameters for each method so that manual tuning is not required. Towards that goal we utilise cluster stabilities to decide on the parameters (which depend on the specific algorithm i.e. kernels in k-means and spectral clustering, linkage method in hierarchical clustering) selected by this toolkit. All available parameters (Table 2) participate in the selection procedure where we measure the average bootstrap stability of the clusters (clusterboot function in R package fpc v2.2-3) using the previously determined optimal k and feature set for each parameter. The parameter that produces the highest stability is used for the optimal clustering run.

Test datasets

Seven datasets were used to validate different capabilities of the Omada package. First, two datasets were simulated by Omada's functions. Function feasibilityAnalysisDataBased() was used to generate a multi-class dataset with 359 samples and 300 genes based on the contents and dimensions of the original RNAseq data[18] and composed of five groups of samples drawn from five different distributions with means (5,16,27,38,50) and sd (1,3,5,7,10), representing the five classes. Function feasibilityAnalysis() simulated a single-class dataset of 100 samples and 100 genes drawn from a single distribution. For the multissue Pan-cancer dataset we downloaded RNAseq expression data for 2244 samples and 253 genes representing three types of cancers: breast (n=1084), lung (n=566) and colon/rectal (n=594) downloaded through cbioportal[59] from TCGA PanCancer Atlas[60]

(PANCAN). The mRNA expression was in the form of z-scores relative to normal samples where we applied an extra step of arcsine normalisation. This type of transformation calculates a (proportional) pseudocount per gene instead of using one constant overall pseudocount, which creates a compression effect that also accommodates genes with low expression values. After filtering for tissue-specific genes[61] for the three cancer types we retained 243 genes. An additional TCGA LUAD dataset was acquired from the GDC data portal[62]. Only patients with Adenocarcinoma, NOS, whose primary tumor site was bronchus and lung with available TPM data were included. No missing information on gender, ethnicity, race and vital status were allowed. To reduce complexity, we additionally remove patients with more than one TPM file. Altogether 240 patients with unique TPM data were merged and genes with average TPM less than 2 or greater than 500 were removed, resulting in 16,088 genes remaining after quality control to be used for further downstream analysis. Next, we utilised a pulmonary arterial hypertension (PAH) dataset (25,955 genes) generated from 359 patient samples with idiopathic (IPAH) or heritable (HPAH) nature. The transcriptomic data can be found in the EGA (the European Genome-phenome Archive) database under accession code EGAS0000100553265[63] (restricted access) and all pre-processing details and parameters used can be found in [18]. We also used an RNA dataset from the whole blood of 238 mothers during midgestation (26-28 weeks of pregnancy), where whole transcriptome library was constructed based on Illumina's standard protocol and quantified using real-time PCR. Sequencing was based on Illumina HiSeq 4000 system. Read counts were extracted from GEO (accession number GSE182409[64]) and were then read into R and converted into TPM using the *convertCounts* function available in the *DGEobj.utils package*. For the purpose of clustering, we mapped the TPM dataset to the list of 24,070 genes used in the PAH dataset described in a previous section. Finally, we downloaded single-cell peripheral blood mononuclear cell (PBMC, 8k and 4k) data from a single healthy donor from the 10X genomics website in cellRanger output. UMI reads were merged and pre-processed using standard Seurat 4.3.0[65] pipeline. Briefly, cells with percent mitochondria exceeding 5 percent, feature counts below 200 or exceeding 2500, as well as empty droplets or doublets, were excluded from further analysis. This procedure aimed to identify and select the top 2000 most variable genes for subsequent downstream analysis, which included data scaling and PCA analysis. The goal of these additional steps was to choose the top 10 principal components (PCs) capable of explaining at least 75 percent of the variation for clustering analysis. For cluster identification, we initially constructed a K-nearest neighbour (KNN) graph and a shared nearest neighbour (SNN) graph. Subsequently, the Louvain method was applied using default settings.

Cell types identification involved a consensus approach, combining insights from four different methods: the candidate marker method based on the Garnette database[66], RCAv2 (utilizing GlobalPanel), scType[67,68] (leveraging the Immune system database), and DISCO[69] (focusing on blood tissue), Subsequently, cell type-specific quality control (QC) measures were implemented to eliminate any outlier cells within each identified cell type. In total, 9303 samples with 19171 features were retained, covering seven major cell types. For the clustering analysis, we deliberately selected only four clusters, emphasising those where the majority of cells predominated, for further in-depth analysis.

Evaluation of run time and memory usage

Scalability performance was assessed by incrementally increasing the number of samples and features while maintaining the upper limit of clusters (kmax). Each combination of samples and features underwent 10 iterations, and the average run time and memory usage were computed. Run time measurements were conducted using the sys.time() function, capturing the time difference between the initiation and completion of Omada's pipeline. Concurrently, memory usage was evaluated using the memory_used() function from the "pryr" package in R. This comprehensive analysis provided insights into how adjustments in the parameters such as the number of samples, number of features can *influence* both the computational time and memory requirements of Omada's processing. When benchmarking Omada on simulated datasets the upper number of clusters was set to 3. Analyses were run on a linux-based operating system, Intel 8268 48 cores platform at 2.9 GHz Nvidia V-100.

Benchmarking Omada against other clustering approaches

To benchmark Omada against other clustering approaches we performed clustering analyses using the algorithms (hierarchical, k-means, SOM, AP) utilised in [13] on the TCGA dataset (3 cancer types, LUAD, BRCA, COAD) as described in section Test datasets. We extracted the resulting clusters for k = 3 and calculated the adjusted Rand Index to assess the similarity between the clustering results of Omada and each of the additional algorithms.

Results

Omada was applied to seven diverse gene expression datasets to demonstrate its utility in guiding cluster analysis and identifying plausible subgroups of samples. Two datasets were simulated by our tools. The simulated dataset with multiple distinct classes was used to determine Omada's ability to accurately estimate *k* with reasonable stability when we know the existence of sample classes. In contrast, samples in the single-class simulated dataset were drawn from a single class and used to demonstrate the toolkit's ability to point towards the lack of sample subgroups by indicating inconclusive low scores throughout the analysis. A multi-tissue PANCAN dataset was introduced to assess Omada's capability to generate signal-based clusters that closely follow the tissue-specific patient sample distributions. A second TCGA LUAD dataset was also included to determine whether Omada can accurately recapitulate similar outcomes in terms of overall survival based on the cluster membership identified. To determine whether Omada can identify distinct heterogeneous subgroups from data without any prior classification information but potential present heterogeneity, we used a whole blood RNAseq dataset from patients with pulmonary arterial hypertension (PAH)[18]. In addition, implementation of the toolkit on a whole blood expression dataset collected from women during pregnancy (GUSTO) was included to demonstrate a case with potential technical biases and no known subgroups since it is composed of healthy participants. Finally, single-cell PBMC data from a healthy donor was incorporated to assess Omada's capability in handling high-dimensional datasets. This addition served as a means to assess scalability performance in terms of both run time and memory usage.

For the above, we measured its consistency on algorithm, feature and number of clusters (*k*) selection and the stability of the generated clusters for a particular *k* ($stability_k$) and the average across *k's* ($stability_{avg}$). It's important to note that the value of this validation is derived from the fact that unstable clusters should not be interpreted as this instability comes from problematic data or an incorrect approach. However, cluster stability only provides a mechanistic way to assess the underlying data structure and further information is required to fully biologically validate the clusters[38].

Identifying known clusters

Single-class dataset: Homogeneously simulated dataset

To demonstrate Omada's ability to identify datasets without any present clusters where all patients belong to one class, we used the single-class simulated dataset (see *Test datasets* in *Methods*). All potential *k* of two or higher achieved low scores with average and maximum stabilities of 45% and 55%, respectively (Supplementary Table 1). It is recommended to avoid clustering analysis on such low score datasets and instead opt for scores of at least 60%. Ideally, stabilities of 80-90% are considered very strong[70], however the potential of several signals in transcriptomic data and the exploration across multiple *k* generally decreases the output stability to an acceptable threshold of 60-70%. Next, Figure 3A shows the overall low partitional consistencies (averaged over all tested *k*) for all algorithms with spectral average partition agreement of 52%, kmeans average partition agreement of 3% and hierarchical average partition agreement of 26%. With the best performing algorithm showing an agreement of around 50% we can assume that the tested algorithms are randomly assigning memberships, therefore we cannot achieve a robust model with the current data. When using spectral clustering to select the most appropriate set of genes, the cluster stability rapidly dropped below 50% when using more than 20 genes (Figure 3B) indicating that the algorithm got worse in assigning memberships as we considered more simulated genes. Finally, the ensemble voting step showed the majority of the votes supporting five clusters (Figure 3C), a significant variation from the single simulated class of this dataset. In such unexpected outputs, one should examine the generated metric scores (Table 3; Supplementary Table 3).

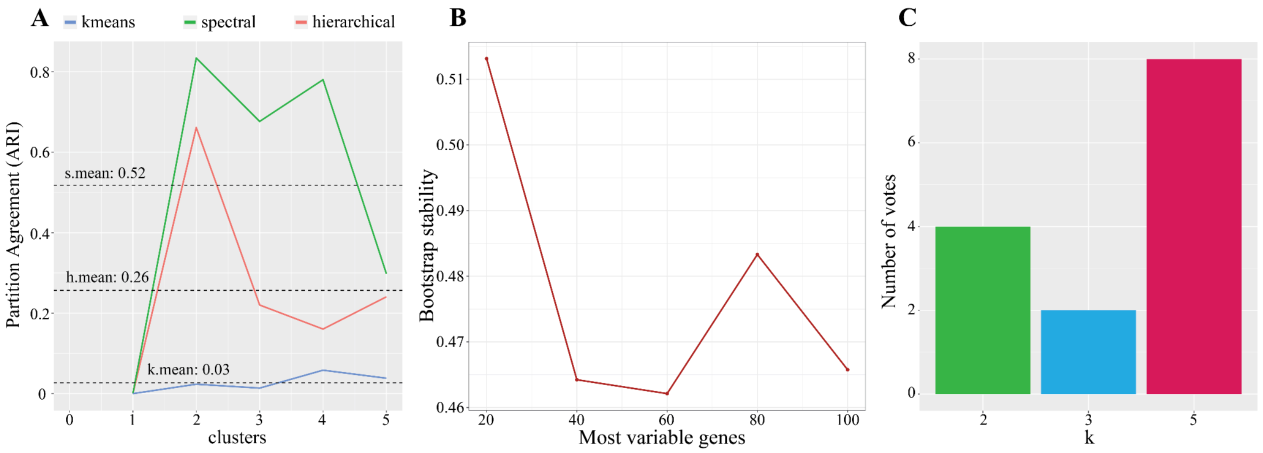

Figure 3: Performance criteria for single-class simulated dataset. The results demonstrate low scores for the majority of steps. A) shows the average partition agreement of all three algorithms below the 52% mark indicating very unstable clustering runs overall. Utilising spectral clustering (highest partition agreement) for all downstream steps, in B) we observe that the stability of every possible subset of genes does not surpass 51.3% underlying overall unstable clusters. C) shows five clusters as the first estimate (voted by 8 metrics), significantly different from the one class this dataset contains (in this case 14 scores were used as certain metrics are (infrequently) unable to produce a score and are therefore omitted from voting).

Multi-class dataset: Five distinct simulated expression classes representing heterogeneity

Omada's basic function is to help identify samples that come from different sources and group together samples that come from the same source. Towards that end, we simulated a dataset with five sets of expression profiles with 50 samples each and 120 genes sourced from five unique distributions of expression data that represent heterogeneity within our samples. The means and standard deviations of each class are presented in Figure 4A, depicting the expression differences. Additionally, the empirical Cumulative Distribution Functions (ECDFs) of the five simulated classes (Figure 4B) as well as the high average Kolmogorov-Smirnov distances (D_{avg}=88.3%, Supplementary Table 2) show distinct differences between the distributions in respect to the expression in the simulated RNAseq dataset. To demonstrate the effect of different sample and gene numbers, multiple datasets were simulated with an increasing number of samples and genes (Figure 4C). The calculated cluster stabilities, where each value represents the stability over a range of *k* and a specific number of samples and features, show five or less samples per class provide highly unstable and unreliable clusters. The minimum acceptable stability threshold of 60% was achieved with at least 20 samples and a reliable stability of 75% was achieved using 1000 samples. The majority of metric scores are worse in testing single-class instead of multi-class simulations (Supplementary Table 3), especially in lower compactness and shorter distance between clusters.

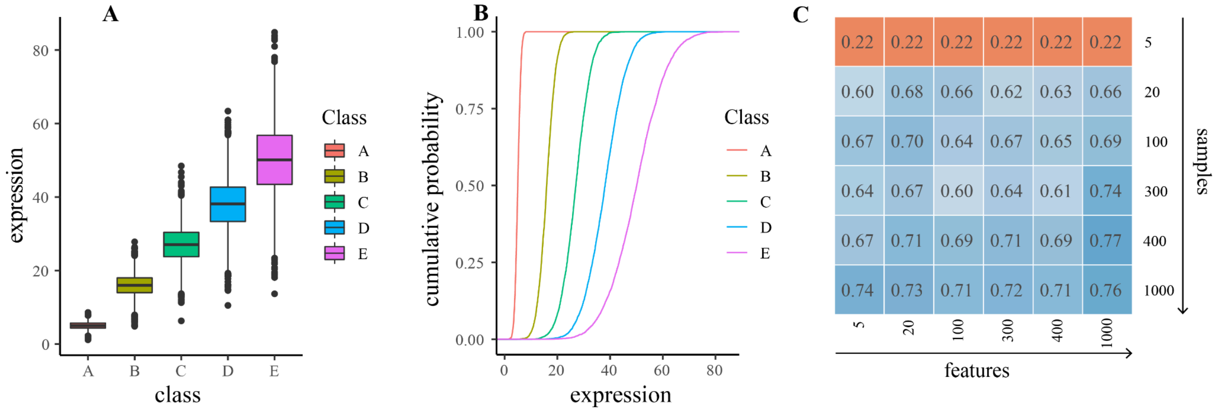

Figure 4: A) Expression boxplots for the five clusters showing the means and standard deviations B) The cumulative probability (as calculated from the empirical cumulative distribution function) for the five clusters calculated by a two sided Kolmogorov-Smirnov Test C) Average over-k stabilities for simulated datasets of increasing sample and gene numbers. A small number of samples consistently provides extremely unstable clusters (orange) while increasing both numbers consistently produces datasets that pass the stability threshold of 60% (blue).

To test the ability of the clustering tools to produce stable clusters in various contexts we first apply them in sequence on strategically simulated data. The data are composed of distinct classes (based on class mean m_{class} and standard deviation sd_{class}) and due to that strong signal our tools are expected to determine an accurate k with reasonable stability, scoring above 60%. To allow for a more direct comparison, we used a multi-class simulated dataset from random sampling of real RNAseq data[18]. When considering ranges of k we are using [2, 6] clusters to observe a broader range of results for comparison reasons. The clustering feasibility tool showed that the highest stability was 78% (Supplementary Table 4), providing a strong indication of stability across our clusters. Since we selected a limited range of $k \in [2, 6]$ where the stability should remain high, the averaged-over-every tested k-stability (stability$_{avg}$) of 72% indicates a dataset of adequate size and class definition to proceed to clustering analysis. It should be noted that when large ranges of k are selected the average stability will naturally decrease as the calculations will take into account k's much larger or smaller than the actual number of classes in the data. In such cases the user can review the individual k stabilities generated as part of this tool to conclude whether those values are satisfying i.e a minimum of 60%. Next, we calculated the partitioning agreement of three clustering algorithms and spectral clustering showed the highest average score of 56% (Figure 5A and Supplementary Table 4). Partitioning agreement scores should be interpreted across algorithms applied on the same dataset rather than as absolute values keeping in mind that a score below 50% represents a random partitioning and subsequently a non-robust clustering. In the subsequent feature selection step, the highest average stability was registered when using all 300 features (stability$_{avg}$ = 78%, Supplementary Table 4), not discarding any feature as they all demonstrated very similar variance due to the nature of the simulated data. Finally, 8 out of 15 internal metrics voted five clusters as the optimal number during the k estimation step (Figure 5B) providing a confident estimation above 50%.

Discriminating cancer types from pan cancer tissue expression data

An integral capability of Omada is to accurately stratify patients according to any biologically relevant signal present in expression data and detect differences stemming from genes, pathways, tissues etc. Real multi-tissue samples are often the focus of exploratory studies as they present cell-type differences but still unknown factors that may discriminate them. Using expression data from multiple cancer types (Pan-cancer dataset as described in *Test datasets* in *Methods*), we expect our tools to identify clusters that are consistent with the samples' tissues of origin. Due to the different types of tumours we explored the potential cluster range of [2, 5] for each pipeline step.

The clustering feasibility of the dataset (2244 samples, 243 genes) presented an average stability of 88% and maximum stability of 100% (Supplementary Table 5) providing confidence for the downstream analysis. Spectral clustering showed the highest consistency (partition $agreement_{avg}$ = 63% closely resembling the simulated multiclass dataset, Figure 5A) and was therefore deemed as the most robust. In this example hierarchical clustering showed high instability, as shown in Figure 5A, demonstrating the importance of selecting the appropriate algorithm to create a robust model. According to our selection tool, all 243 genes produced the most stable set of clusters with a stability of 96% (Supplementary Table 5) which coupled with the high algorithm robustness indicated a model that most likely detects a signal in the data. Additionally, a very important observation is that all genes were deemed important to produce nearly perfectly stable clusters agreeing with the filtering of genes based on the cancer type annotations we performed prior to this clustering analysis. The ensemble voting tool estimated our dataset to contain three clusters of samples with the support of 57% of the metrics (Figure 5B). When comparing these results with the simulated five-class dataset, both achieved higher certainty on the five clusters (>50%, Figure 5B) reflecting the rigid differences between the clusters when dealing with cancer tissues and simulated classes. In the case of the PANCAN partitioning, the breast, lung and colon/rectal samples almost perfectly grouped in their respective clusters (Figure 5C).

Omada also has the ability to supply additional information for assessing whether the existing dataset lacks signal for potential clustering.In this context, Omada was utilised to analyse a separate TCGA LUAD whole transcriptome data obtained from PMID35664309[62]. The objective was to compare the clustering and overall survival outcomes between Omada and the unsupervised clustering method employed by Pan S et al. We restricted the dataset to 240 patients diagnosed with Adenocarcinoma, NOS, and included 16,088 genes post quality control measures. We anticipate that Omada will categorise patients according to their survival or disease states. The feasibility of clustering demonstrated an average stability of 68%, with a maximum stability of 100%, instilling confidence for subsequent analysis. Spectral clustering exhibited the highest average partition agreement at 48%, with the peak agreement occurring when k = 2. Through cluster voting, we identified 45 patients in cluster 1 and 195 patients in cluster 2.To assess survival differences between the two identified clusters, we employed survival analysis using the Kaplan-Meier method. Remarkably, the identified clusters did not exhibit an overall disparity in survival, as indicated by a p-value of 0.4 (Supplementary Figure 4). This finding aligns with observations in Pan S et al. A plausible explanation for this outcome may be attributed to a lack of signal in the data for clustering, possibly stemming from a low overall average partition agreement score, which falls below 50% based on spectral clustering.

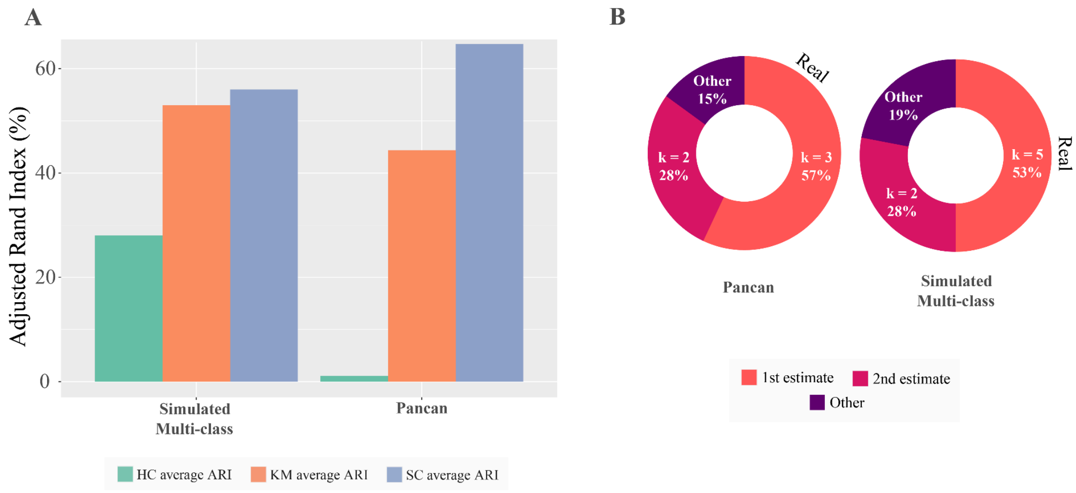

C

Multi-class simulated RNA-seq data

	Class 1	Class 2	Class 3	Class 4	Class 5
**Cluster 1**	72	0	0	0	0
**Cluster 2**	0	0	0	72	0
**Cluster 3**	0	0	72	0	0
**Cluster 4**	0	0	0	0	72
**Cluster 5**	0	71	0	0	0

Pancan multi-tissue RNA-seq data

	BRCA	COAD	LUAD
**Cluster 1**	0	0	503
**Cluster 2**	1084	3	63
**Cluster 3**	0	591	

Figure 5: Performance criteria for two heterogeneous datasets, simulated multi-class and PANCAN dataset. The multi-class dataset contains artificial samples from five distinct clusters and the PANCAN dataset is composed of three different cancer types presenting biological heterogeneity. A) The agreement between the predicted and true clusters (Adjusted Rand Index) from three different clustering algorithms (HC: hierarchical, KM: K-means, SC: spectral clustering) applied to the two datasets. B) Shows the real number of clusters for the dataset (black text) and the two most likely number of clusters k, with estimates of their percent probability. C) The contingency tables of the combinations between generated clusters (1st estimates) and real classes in the datasets. Darker red colour intensity denotes higher frequency.

Not all algorithms can detect the true number of clusters

We tested the ability of four algorithms to cluster samples that belong to three cancer types. As seen on Table 4, hierarchical clustering was not able to identify the three cancer types (ARI = 0.55) as it selected two clusters as most probable. On the contrary, spectral clustering separated the samples most accurately (ARI = 0.83) with a clear preference for three clusters (8/14 votes) closely followed by k-means (ARI = 0.80 and 5/14 votes). Consensus clustering followed with an ARI of 0.78 but a widest spread of estimated k as it selected 5 and 6 clusters as second and third choices respectively. On the simulated dataset (Table 5), k-means estimated the correct number of clusters and managed

to assign the memberships perfectly (ARI = 1). All other algorithms selected a different k and therefore presented a much lower ARI (as comparing a set of two clusters with a set of three clusters cannot result in a high ARI). Both Omada and consensus clustering M3C suggest k = 3 as the second estimate. However, Omada already supplies the memberships for k = 3 as part of the k estimation achieving a perfect ARI of 1 if that partitioning is selected for all three algorithms.

Table 4: The ARI of the top k estimate of various datasets with different numbers of cancer types

Algorithm	Real clusters		
	3	5	7
Hierarchical	0.55 (k=2)	0.13 (k=2)	0.19 (k=2)
k-means	0.80 (k=3)	0.54 (k=4)	0.45 (k=)
Spectral	0.83 (k=3)	0.84 (k=5)	0.58 (k=)
M3C	0.78 (k=3)	0.85 (k=5)	0.63 (k=9)

Table 5: The ARI of the top k estimate of various datasets with different numbers of simulated classes

Algorithm	Real clusters		
	3	5	7
Hierarchical	0.57 (k=2)	0.9 (k=7)	0.9 (k=9)
k-means	1.00 (k=3)	1.00 (k=5)	0.79 (k=8)
Spectral	0.57 (k=2)	1.00 (k=5)	1.00 (k=7)
M3C	0.57 (k=2)	0.78 (k=5)	0.54 (k=4)

RNAseq data from diseased tissue with unknown heterogeneity

It is important for Omada to be able to robustly identify patient subgroups when heterogeneity for the cohort has not been previously characterised. We applied our tools on such a dataset (PAH dataset as described in *Test datasets* in *Methods*) to assess whether they can still produce stable clusters that differ in terms of expression profiles and other phenotypic measures. The feasibility for this dataset's simulation showed an average stability of 61% and a maximum stability of 74% both acceptable to proceed with the clustering analysis (Supplementary Table 6). A notable 13% difference between average and maximum stability provides a positive indication that a specific *k*

might prove significantly more stable downstream. The spectral clustering technique recorded the highest partitional consistency (partition $agreement_{avg}$ = 86% and partition $agreement_{max}$ = 96%) when we examined each algorithm's partition agreement for up to ten clusters. The bootstrapping subset selection tool estimated the 300 most variable genes as the most stable clustering parameter with a maximum stability of 73% (Figure 6A) showing an impressive reduction from the initial gene set (25,955) and ensuring the removal of a lot of data noise. According to the ensemble voting tool two clusters were voted by 71% of the internal metrics followed by k = 3 (14%) and k = 5 (7%). Despite the strong indication of two clusters, k = 5 was selected to prevent loss of information occuring when smaller embedded clusters are disregarded. As shown by the downstream analysis, fully presented in [18], selecting the higher k, even as a second estimate, allowed us to detect strong expression profiles. After considering cluster sizes the three predominant subgroups showed significant differences in expression, immunity and survival profiles as well as risk category distributions (Figure 6B, C).

RNAseq data from healthy whole blood tissue

Next we tested how Omada would discriminate samples from healthy individuals from a single tissue type. Generally, in studies based on a dataset with no discernible heterogeneity to be explored - i.e a dataset without patients of dissimilar outcomes or controls - clustering algorithms may not be robust and may generate variable results. Useful partitioning might still be formed, such as unforeseen disease subgroups, but these observations must be validated. Towards that end, we used the GUSTO RNA whole blood dataset of 238 mothers obtained during mid-gestation, as seen in *Test datasets* in *Methods*. During determining clustering potential our simulated dataset showed $stability_{avg}$ = 56% and $stability_{max}$ = 59% (Supplementary Table 7), a similar low-stability score as in the simulated single-class (45% and 55%). We examined a k-range of [2, 5] where spectral and k-means clustering showed very similar internal average partitional agreements of 61% and 60% and very high maximum agreements of 93% and 88%, respectively. The extremely high agreement scores should be interpreted with caution as they might not reflect a very strong signal but an underlying bias that partitions samples in similar groups repeatedly, over-powering the parameter changes. The 50 most variable genes were estimated to produce the most stable clustering with maximum stability = 71% (Figure 6A). Similarly to the agreement scores, a small number of genes driving the most stable clusters (starting from 24,070 genes) might indicate either a strong expression signal or a pre-existing bias. When estimating the number of clusters, two (46%) and three (40%) clusters were voted by the majority showing a general consensus. Considering all the

above strong indications, we need to assess the dataset and the resulting subgroups for potential biases before relying on the cluster memberships. Towards that end and utilising clinical data, the association results show the dataset might be biased based on technical batches with sequencer machine (Figure 6E) and flowID (Figure 6F) presenting significant differences between clusters (1.39e-03 and 2.55e-06, respectively) with hospital location coming close to significance with p-value = 0.072 (Supplementary Table 8). Additional statistical tests and regression analysis with maternal and foetal physiological and clinical phenotypes did not show any association with the clusters. The expression profiles of the two clusters show visible differences (Figure 6D) as do the t-SNE and PCA analyses (Figure 6H) with the first principal component of the latter explaining 79% of the variance in the GUSTO dataset.

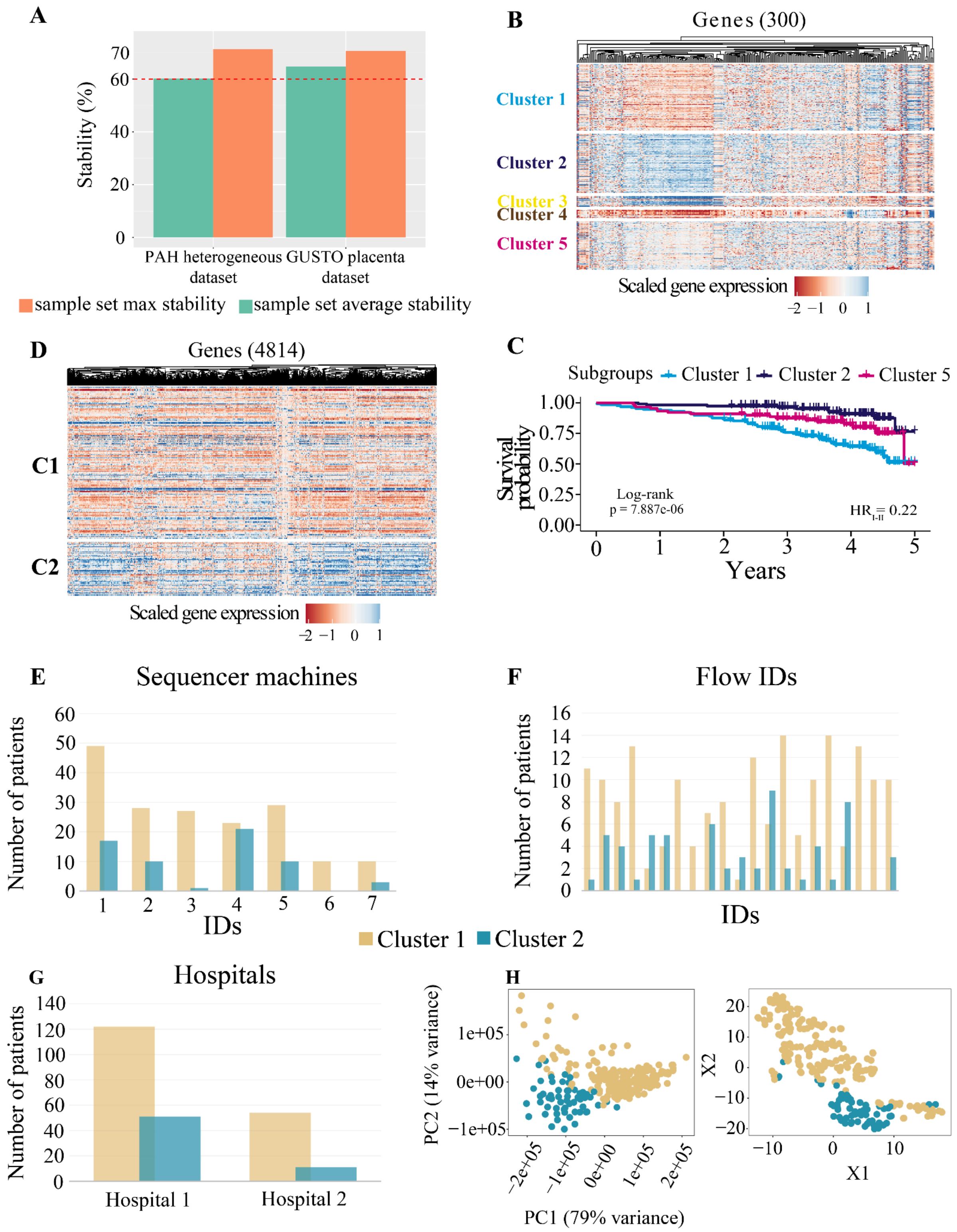

Figure 6: Performance criteria for PAH and GUSTO datasets which have no known subgroups. Panel A) depicts the average and max sample set stabilities (percentage) for both datasets. The red dashed line represents the threshold of a stable clustering (60%). The PAH RNAseq dataset contains expression of IPAH patients with panel B) showing the gene expression heatmap and C) survival profiles for discovered subgroups (subgroups 3 and 4 are omitted as they were not used in downstream analysis due to their size). The GUSTO dataset contains expression from healthy maternal whole blood with panel D) showing the gene expression heatmap. The following panels show the distribution of cluster members across E) sequencer machines(chi-square p-value 1.39e-03), F) flow IDs (chi-square p-value 2.55e-06) and G) hospitals where the data were collected (chi-square p-value 0.072). H) t-SNE and PCA plots of the expression profiles with labelling of the two discovered subgroups.

Scalability Performance

To evaluate and benchmark Omada's scalability, we utilised two distinct groups of datasets: simulated datasets (generated by Omada’s feasibilityAnalysis() function) and subsets of a high-dimensional single-cell PBMC dataset. For each dataset, we ran Omada pipeline on a varying number of samples and features with each combination repeated 10 times. Average run time and memory usage were recorded and presented in Supplementary Figures 2-3, as well as Supplementary Tables 9-12).

Both types of datasets unveiled an increase in run time and memory requirement as number of cells (ie. samples) analyse increased while maintaining kmax and the number of features. A much smaller increase in time was observed when augmenting the number of features (i.e., genes).

When examining the simulated datasets (3 generated classes) setting 500 samples, we noticed a small runtime difference when using 100 and 1000 features (4.2 and 4,8 minutes, respectively, Supplementary Table 9). Similarly, the difference in memory used was not substantial with 201 megabytes and 214 megabytes respectively (Supplementary Table 10). As demonstrated, the increase in number of samples was the only factor that affected Omada’s runtime (Supplementary Figure 2A) but not memory usage (Supplementary Figure 2B). Neither of the above was greatly affected by the increase of features (Supplementary Figure 2C,D).

In the single-cell PBMC data the average time taken and memory consumption for processing 1000 samples with 1000 features amounted to approximately 11.0 minutes (Supplementary Table 11) and 915 megabytes (Supplementary Table 12), respectively. Likewise, the average time taken and memory consumption to analyse 1000 samples and 1000 features on the simulated dataset was 20.3 minutes and 220.8 megabytes. Across 10 iterations, memory usage remained consistent, with only slight variations in run time (Supplementary Figure 3). These variations can be attributed to the selection of fewer or more genes during the feature selection step. It is important to also note that depending on the sparsity/complexity of the input matrix, there may be variations in memory usage and computational time.

Benchmarking with other clustering algorithms

All tested algorithms except affinity propagation (AP) provided nearly identical subgroups as shown in Supplementary Table 13. The generated subgroups were compared to the real cancer classes (Supplementary Table 14) with Omada, hierarchical, k-means and SOM demonstrating high accuracy in identifying the cancer types (~85%) while AP showing considerably lower performance (~37%). When applying Omada to a subset of the single-cell data containing 1000 samples, wherein 250 cells were randomly selected from each of the four major cell types (B cells, monocytes, CD4 T cells, and CD8 T cells), along with 10000 random features, spectral clustering revealed the highest agreement in partitioning when k was set to 2, resulting in a maximum partition agreement of 1. Subsequent cluster voting indicated that 57% of the votes supported a 2-cluster solution, while 28% supported a 3-cluster solution based on internal metrics. However, to avoid information loss, a k value of 3 was selected. Notably, the analysis revealed distinct cluster compositions: Cluster 1 comprised cells of B cell origin, Cluster 2 composed of monocytes, and Cluster 3 contained cells originating from T cells, including both CD4 and CD8 T cells. To better distinguish between CD4 and CD8 T cells, a larger dataset is required.

Discussion

Omada is designed to enable the assessment of multiple clustering solutions arising from transcriptomic studies of human samples. With the plurality of unsupervised methods available and their specialised nature, the selection of the most appropriate approach is a multi-factor problem and suggesting a general system of recommendation is impossible without considering each dataset in advance[21]. Many decisions on technical parameters are required from the user of clustering approaches in order to get a meaningful set of subgroups [71]. To assist with this problem, our toolkit initially assesses the potential of a target dataset and provides recommendation for the most appropriate clustering algorithm, gene set and number of possible subgroups. Omada can also be run as a pipeline, therefore, does not require deep prior knowledge of the algorithms, parameters and metrics by the user. All outputs of the pipeline, intermediate and final, are observable and each step is justified by multiple measures and indices representing widely used clustering techniques. We showed that a user of Omada can conduct analysis to assess the feasibility of applying different

clustering algorithms and parameters to different RNAseq datasets from healthy and diseased tissue samples.

Conducting unsupervised learning of expression datasets is often not a straightforward task as the true heterogeneity of the health condition or tissue of origin is unknown. No methods or metrics can give a definitive answer without validation from external biological or clinical insights, therefore, the output of each algorithm has to be used with caution. Determining the dataset clustering potential is an indication rather than a clear sign that partitioning the dataset will yield informational subgroups[71]. Additionally, clustering can often contain non-deterministic steps (i.e. k-means initial cluster centers selection[40]) allowing for each function to behave slightly differently between similar runs. In order to evaluate the uncertainty and provide a robust set of tools, Omada has been applied to gene expression datasets where we have prior confidence in the number of clusters (simulated and cancer type data) and where we have little prior knowledge (GUSTO whole blood, PAH). Even in data from healthy tissue, such as from GUSTO whole blood, small technical biases can result in prominent clusters detected by Omada. It is also possible for Omada to hint towards the existence of a single homogeneous cluster by consistently revealing low partition agreement and stability scores across multiple functions, as demonstrated in our single-class datasets. Furthermore, Omada can help in selecting a small group of genes with potential partitioning capabilities as the feature selection step is expected to greatly reduce the number of genes which in most cases count to thousands.

This toolkit currently encompasses common clustering techniques, metrics and test datasets, but its modular nature allows its extension to more specific clustering problems, such as for single cell and spatial data. Furthermore, the structure of this toolkit allows for additional algorithms and metrics to be added to the pool of clustering methods to be evaluated during an analysis run. Systematic empirical testing of algorithms using Omada's approach may result in more robust and better justified clustering outputs for a variety of biomedical studies.

Code Availability

Code is available on github (https://github.com/BioSok/omada) and as a bioconductor software package (Omada) at 10.18129/B9.bioc.omada.

Data Availability

The expression datasets used in this work can be accessed through the following sources: The two simulated, by Omada, datasets (single and multi-class) can be accessed and downloaded at https://github.com/BioSok/OmadaSimulatedDatasets. The Pan cancer tissue expression data can be accessed through (https://www.cbioportal.org/study/summary?id=brca_tcga_pan_can_atlas_2018, https://www.cbioportal.org/study/summary?id=coadread_tcga_pan_can_atlas_2018, https://www.cbioportal.org/study/summary?id=luad_tcga_pan_can_atlas_2018). A second TCGA LUAD dataset can be accessed through the Genomic Data Commons (GDC) Data Portal (https://portal.gdc.cancer.gov/projects/TCGA-LUAD) under dbGAP study accession phs000178. The transcriptomic data used in this study can be accessed through the EGA (the European Genome-phenome Archive) database under accession code EGAS00001005532. In compliance with the Ethics under which these data and samples have been collected, the transcriptomic data are available through restricted access for approved researchers who agree to the conditions of use, i.e. keeping it secure and only using it for approved purposes. To apply for access please contact cohortcoordination@medschl.cam.ac.uk. You will receive an application form within 30 days. The 'UK National PAH Cohort Study Data Access Committee' will review requests within 3 months of receipt of the completed application form and if approved, provide details for access to the RNAseq data stored at the EGA. All requesters must agree to the data access conditions found in EGA. The data used to generate statistics, plots and figures are accessible through our interactive portal found in https://sheffield-university.shinyapps.io/ipah-rnaseq-app/. The GUSTO expression dataset is available in NCBI Gene Expression Omnibus (GEO; https://www.ncbi.nlm.nih.gov/geo/) under the accession numbers GSE182409 (Corresponding Reviewer token number: qjolmmeudnofnsv). The Single-cell PBMC 8k and 4k data is publicly available through (https://www.10xgenomics.com/).

List of datasets used:

- Single-class simulated dataset (100x100): Simulated by using specific mean and standard deviation. (https://github.com/BioSok/OmadaSimulatedDatasets)
- Multi-class simulated dataset (359x300): Simulated by using specific means and standard deviations. (https://github.com/BioSok/OmadaSimulatedDatasets)
- Pan-cancer dataset (2244x253): Sourced from 3 cancer type datasets: Breast cancer (https://www.cbioportal.org/study/summary?id=brca_tcga_pan_can_atlas_2018), Colorectal cancer (https://www.cbioportal.org/study/summary?id=coadread_tcga_pan_can_atlas_2018) and Lung cancer (https://www.cbioportal.org/study/summary?id=luad_tcga_pan_can_atlas_2018).

- Second TCGA-LUAD dataset (240x60661): (https://portal.gdc.cancer.gov/projects/TCGA-LUAD) , Primary tumour: Lung & Bronchus, Diagnosis: Adenocarcinoma, NOS.
- PAH dataset (359x300): Can be found in the EGA (the European Genome-phenome Archive) database https://ega-archive.org/studies/EGAS00001005532, needs permission from its Data Access Committee.
- GUSTO dataset (238x24,070): Can be found in the GEO database with accession number GSE182409.
- Single-cell PBMC dataset (8K and 4K from healthy donor): (https://www.10xgenomics.com/), Cell Ranger output, pipeline version 2.1.0.

Acknowledgements

The UK National Cohort of Idiopathic and Heritable PAH is supported by grants from the British Heart Foundation (SP/12/12/29836 & SP/18/10/33975) and the UK Medical Research Council (MR/K020919/1). Additional samples from the Sheffield Teaching Hospitals Observational Study of Pulmonary Hypertension, Cardiovascular and other Respiratory Diseases were supported by British Heart Foundation (PG/11/116/29288). DW is supported by the Academy of Medical Sciences Professorship (APR7_1002) and the Engineering and Physical Sciences Research Council (EP/V029045/1). S.K. is supported by a Donald Heath Ph.D. Studentship award and A*STAR Research Attachment Programme (ARAP) award.

The GUSTO study group includes Allan Sheppard, Amutha Chinnadurai, Anne Eng Neo Goh, Anne Rifkin-Graboi, Anqi Qiu, Arijit Biswas, Bee Wah Lee, Birit Froukje Philipp Broekman, Boon Long Quah, Chai Kiat Chng, Cheryl Shufen Ngo, Choon Looi Bong, Christiani Jeyakumar Henry, Daniel Yam Thiam Goh, Doris Ngiuk Lan Loh, Fabian Kok Peng Yap, George Seow Heong Yeo, Helen Yu Chen, Hugo P. S. van Bever, Iliana Magiati, Inez Bik Yun Wong, Ivy Yee-Man Lau, Jeevesh Kapur, Jenny L. Richmond, Jerry Kok Yen Chan, Joanna Dawn Holbrook, Johan G. Eriksson, Joshua J. Gooley, Keith M. Godfrey, Kenneth Yung Chiang Kwek, Kok Hian Tan, Krishnamoorthy Naiduvaje, Leher Singh, Lin Lin Su, Lourdes Mary Daniel, Lynette Pei-Chi Shek, Marielle V. Fortier, Mark Hanson, Mary Foong-Fong Chong, Mary Rauff, Mei Chien Chua, Michael J. Meaney, Mya Thway Tint, Neerja Karnani, Ngee Lek, Oon Hoe Teoh, P. C. Wong, Peter David Gluckman, Pratibha Keshav Agarwal, Rob Martinus van Dam, Salome A. Rebello, Seang

Mei Saw, Shang Chee Chong, Shirong Cai, Shu-E Soh, Sok Bee Lim, Stephen Chin-Ying Hsu, Victor Samuel Rajadurai, Walter Stunkel, Wee Meng Han, Wei Wei Pang, Yap Seng Chong, Yin Bun Cheung, Yiong Huak Chan and Yung Seng Lee.

Author Contributions

SK and DW conceived the tools. SK undertook computational work and drafted the work with DW. AL, CR, TPF and MW participated in the data acquisition of the work. All authors revised it critically for important intellectual content; and gave final approval of the version submitted for publication. The author(s) declare(s) that they have no competing interests.

References

1. Yu, N. Y.-L. *et al.* Complementing tissue characterization by integrating transcriptome profiling from the Human Protein Atlas and from the FANTOM5 consortium. *Nucleic Acids Res.* **43**, 6787–6798 (2015).
2. Keen, J. C. & Moore, H. M. The Genotype-Tissue Expression (GTEx) Project: Linking Clinical Data with Molecular Analysis to Advance Personalized Medicine. *J Pers Med* **5**, 22–29 (2015).
3. Uhlén, M. *et al.* Proteomics. Tissue-based map of the human proteome. *Science* **347**, 1260419 (2015).
4. Wang, L. *et al.* RNA sequencing-based longitudinal transcriptomic profiling gives novel insights into the disease mechanism of generalized pustular psoriasis. *BMC Med. Genomics* **11**, 52 (2018).
5. Neff, R. A. *et al.* Molecular subtyping of Alzheimer's disease using RNA sequencing data reveals novel mechanisms and targets. *Sci Adv* **7**, (2021).
6. Saeidian, A. H., Youssefian, L., Vahidnezhad, H. & Uitto, J. Research Techniques Made Simple: Whole-Transcriptome Sequencing by RNA-Seq for Diagnosis of Monogenic Disorders. *J. Invest. Dermatol.* **140**, 1117–1126.e1 (2020).
7. Tran, H. T. N. *et al.* A benchmark of batch-effect correction methods for single-cell RNA sequencing data. *Genome Biol.* **21**, 12 (2020).
8. Xing, Q. R. *et al.* Unraveling Heterogeneity in Transcriptome and Its Regulation Through Single-Cell

Multi-Omics Technologies. *Front. Genet.* **11**, 662 (2020).

9. Firth, A. L., Mandel, J. & Yuan, J. X.-J. Idiopathic pulmonary arterial hypertension. *Dis. Model. Mech.* **3**, 268–273 (2010).
10. Koirala, B. *et al.* Heterogeneity of Cardiovascular Disease Risk Factors Among Asian Immigrants: Insights From the 2010 to 2018 National Health Interview Survey. *J. Am. Heart Assoc.* **10**, e020408 (2021).
11. Rivera-Andrade, A. & Luna, M. A. Trends and heterogeneity of cardiovascular disease and risk factors across Latin American and Caribbean countries. *Prog. Cardiovasc. Dis.* **57**, 276–285 (2014).
12. Manchia, M., Cullis, J., Turecki, G., Rouleau, G. A. & Uher, R. The impact of phenotypic and genetic heterogeneity on results of genome wide association studies of complex diseases. *PLoS One* (2013).
13. Vidman, L., Källberg, D. & Rydén, P. Cluster analysis on high dimensional RNA-seq data with applications to cancer research - An evaluation study. *PLoS One* **14**, e0219102 (2019).
14. Ren, Z., Wang, W. & Li, J. Identifying molecular subtypes in human colon cancer using gene expression and DNA methylation microarray data. *Int. J. Oncol.* **48**, 690–702 (2016).
15. Sotiriou, C., Neo, S. Y. & McShane, L. M. Breast cancer classification and prognosis based on gene expression profiles from a population-based study. *Proceedings of the* (2003).
16. Lapointe, J. *et al.* Gene expression profiling identifies clinically relevant subtypes of prostate cancer. *Proc. Natl. Acad. Sci. U. S. A.* **101**, 811–816 (2004).
17. Wu, F. *et al.* Single-cell profiling of tumor heterogeneity and the microenvironment in advanced non-small cell lung cancer. *Nat. Commun.* **12**, 2540 (2021).
18. Kariotis, S. *et al.* Biological heterogeneity in idiopathic pulmonary arterial hypertension identified through unsupervised transcriptomic profiling of whole blood. *Nat. Commun.* **12**, 7104 (2021).
19. Xu, D. & Tian, Y. A Comprehensive Survey of Clustering Algorithms. *Annals of Data Science* vol. 2 165–193 Preprint at https://doi.org/10.1007/s40745-015-0040-1 (2015).
20. Reddy, C. K. & Vinzamuri, B. A Survey of Partitional and Hierarchical Clustering Algorithms. *Data Clustering* 87–110 Preprint at https://doi.org/10.1201/9781315373515-4 (2018).
21. Jamail, I. & Moussa, A. Current State-of-the-Art of Clustering Methods for Gene Expression Data with RNA-Seq. in *Applications of Pattern Recognition* (IntechOpen, 2020).
22. Ezugwu, A. E. *et al.* A comprehensive survey of clustering algorithms: State-of-the-art machine

learning applications, taxonomy, challenges, and future research prospects. *Eng. Appl. Artif. Intell.* **110**, 104743 (2022).

23. Suzuki, R. & Shimodaira, H. Pvclust: an R package for assessing the uncertainty in hierarchical clustering. *Bioinformatics* **22**, 1540–1542 (2006).
24. Gagolewski, M. genieclust: Fast and robust hierarchical clustering. *SoftwareX* **15**, 100722 (2021).
25. Mouselimis, L., Sanderson, C., Curtin, R. & Agrawal, S. ClusterR: Gaussian mixture models, k-means, mini-batch-kmeans, k-medoids and affinity propagation clustering. *R package version*.
26. Azzalini, A. & Menardi, G. Clustering Via Nonparametric Density Estimation: the R Package pdfCluster. *arXiv [stat.CO]* (2013).
27. Walesiak, M., Dudek, A. & Dudek, M. A. clusterSim package. Preprint at http://keii.ue.wroc.pl/clusterSim/clusterSim.pdf (2011).
28. Scrucca, L. & Raftery, A. E. clustvarsel: A Package Implementing Variable Selection for Gaussian Model-Based Clustering in R. *J. Stat. Softw.* **84**, (2018).
29. Marbac, M. & Sedki, M. VarSelLCM: an R/C++ package for variable selection in model-based clustering of mixed-data with missing values. *Bioinformatics* **35**, 1255–1257 (2019).
30. Thrun, M. C. & Ultsch, A. Clustering benchmark datasets exploiting the fundamental clustering problems. *Data Brief* **30**, 105501 (2020).
31. Wang, C., Gao, X. & Liu, J. Impact of data preprocessing on cell-type clustering based on single-cell RNA-seq data. *BMC Bioinformatics* **21**, 440 (2020).
32. Eijssen, L. M. T. *et al.* User-friendly solutions for microarray quality control and pre-processing on ArrayAnalysis.org. *Nucleic Acids Res.* **41**, W71–6 (2013).
33. Andrews, S. & Others. FastQC: a quality control tool for high throughput sequence data. Preprint at (2010).
34. Bolger, A. M., Lohse, M. & Usadel, B. Trimmomatic: a flexible trimmer for Illumina sequence data. *Bioinformatics* vol. 30 2114–2120 Preprint at https://doi.org/10.1093/bioinformatics/btu170 (2014).
35. D'haene & Hellemans. The importance of quality control during qPCR data analysis. *Int. Drug Discov.*
36. Baccarella, A., Williams, C. R., Parrish, J. Z. & Kim, C. C. Empirical assessment of the impact of sample number and read depth on RNA-Seq analysis workflow performance. *BMC Bioinformatics* **19**, 423 (2018).

37. Wang, J., Yue, S., Yu, X. & Wang, Y. An efficient data reduction method and its application to cluster analysis. *Neurocomputing* **238**, 234–244 (2017).

38. Hennig, C. Cluster-wise assessment of cluster stability. *Comput. Stat. Data Anal.* **52**, 258–271 (2007).

39. Hartigan, J. A. *Clustering Algorithms*. (John Wiley & Sons, Inc., USA, 1975).

40. Dhillon, I. S. *A Unified View of Kernel K-Means, Spectral Clustering and Graph Cuts*. (Computer Science Department, University of Texas at Austin, 2004).

41. Ng, A. Y., Jordan, M. I. & Weiss, Y. On Spectral Clustering: Analysis and an algorithm. in *Advances in Neural Information Processing Systems 14* (eds. Dietterich, T. G., Becker, S. & Ghahramani, Z.) 849–856 (MIT Press, 2002).

42. Rodriguez, M. Z. *et al.* Clustering algorithms: A comparative approach. *PLoS One* **14**, e0210236 (2019).

43. Rand, W. M. Objective Criteria for the Evaluation of Clustering Methods. *J. Am. Stat. Assoc.* **66**, 846–850 (1971).

44. Rousseeuw, P. J. Silhouettes: A graphical aid to the interpretation and validation of cluster analysis. *Journal of Computational and Applied Mathematics* vol. 20 53–65 Preprint at https://doi.org/10.1016/0377-0427(87)90125-7 (1987).

45. Polikar, R. Ensemble based systems in decision making. *IEEE Circuits and Systems Magazine* **6**, 21–45 (2006).

46. Calinski, T. & Harabasz, J. A dendrite method for cluster analysis. *Communications in Statistics - Theory and Methods* vol. 3 1–27 Preprint at https://doi.org/10.1080/03610927408827101 (1974).

47. Dunn†, J. C. Well-Separated Clusters and Optimal Fuzzy Partitions. *Journal of Cybernetics* vol. 4 95–104 Preprint at https://doi.org/10.1080/01969727408546059 (1974).

48. Pakhira, M. K., Bandyopadhyay, S. & Maulik, U. Validity index for crisp and fuzzy clusters. *Pattern Recognition* vol. 37 487–501 Preprint at https://doi.org/10.1016/j.patcog.2003.06.005 (2004).

49. Kendall, M. G. A New Measure of Rank Correlation. *Biometrika* vol. 30 81 Preprint at https://doi.org/10.2307/2332226 (1938).

50. Baker, F. B. & Hubert, L. J. Measuring the Power of Hierarchical Cluster Analysis. *Journal of the American Statistical Association* vol. 70 31–38 Preprint at https://doi.org/10.1080/01621459.1975.10480256 (1975).

51. Hubert, L. J. & Levin, J. R. A general statistical framework for assessing categorical clustering in free recall. *Psychological Bulletin* vol. 83 1072–1080 Preprint at https://doi.org/10.1037//0033-2909.83.6.1072 (1976).
52. Davies, D. L. & Bouldin, D. W. A Cluster Separation Measure. *IEEE Transactions on Pattern Analysis and Machine Intelligence* vol. PAMI-1 224–227 Preprint at https://doi.org/10.1109/tpami.1979.4766909 (1979).
53. McClain, J. O. & Rao, V. R. CLUSTISZ: A program to test for the quality of clustering of a set of objects. *J. Mark. Res.* **12**, 456–460 (1975).
54. Halkidi, M., Batistakis, Y. & Vazirgiannis, M. On Clustering Validation Techniques. *J. Intell. Inf. Syst.* **17**, 107–145 (2001).
55. Ray, S. & Turi, R. H. Determination of number of clusters in k-means clustering and application in colour image segmentation. in *Proceedings of the 4th international conference on advances in pattern recognition and digital techniques* 137–143 (Citeseer, 1999).
56. Rohlf, F. J. Methods of Comparing Classifications. *Annual Review of Ecology and Systematics* vol. 5 101–113 Preprint at https://doi.org/10.1146/annurev.es.05.110174.000533 (1974).
57. Halkidi, M. & Vazirgiannis, M. Clustering validity assessment: finding the optimal partitioning of a data set. *Proceedings 2001 IEEE International Conference on Data Mining* Preprint at https://doi.org/10.1109/icdm.2001.989517.
58. Song, Y. Class compactness for data clustering. in *2010 IEEE International Conference on Information Reuse & Integration* 86–91 (IEEE, 2010). doi:10.1109/IRI.2010.5558958.
59. cBioPortal for Cancer Genomics. https://www.cbioportal.org/datasets.
60. Weinstein, J. N. *et al.* The Cancer Genome Atlas Pan-Cancer analysis project. *Nature Genetics* vol. 45 1113–1120 Preprint at https://doi.org/10.1038/ng.2764 (2013).
61. Kim, P. *et al.* TissGDB: tissue-specific gene database in cancer. *Nucleic Acids Res.* **46**, D1031–D1038 (2018).
62. Pan, S. *et al.* Comprehensive Analysis of Programmed Cell Death Signature in the Prognosis, Tumor Microenvironment and Drug Sensitivity in Lung Adenocarcinoma. *Front. Genet.* **13**, 900159 (2022).
63. Kariotis, S. & Jammeh, E. *BioSok/spectral_clustering_of_IPAH: v1.0.1*. (2021). doi:10.5281/zenodo.5549872.

64. Pan, H. *et al.* Integrative Multi-Omics database (iMOMdb) of Asian Pregnant Women. *Hum. Mol. Genet.* (2022) doi:10.1093/hmg/ddac079.

65. Hao, Y. *et al.* Integrated analysis of multimodal single-cell data. *Cell* **184**, 3573–3587.e29 (2021).

66. Pliner, H. A., Shendure, J. & Trapnell, C. Supervised classification enables rapid annotation of cell atlases. *Nat. Methods* **16**, 983–986 (2019).

67. Li, H. *et al.* Reference component analysis of single-cell transcriptomes elucidates cellular heterogeneity in human colorectal tumors. *Nat. Genet.* **49**, 708–718 (2017).

68. Schmidt, F. *et al.* RCA2: a scalable supervised clustering algorithm that reduces batch effects in scRNA-seq data. *Nucleic Acids Res.* **49**, 8505–8519 (2021).

69. Li, M. *et al.* DISCO: a database of Deeply Integrated human Single-Cell Omics data. *Nucleic Acids Res.* **50**, D596–D602 (2022).

70. Tibshirani, R. & Walther, G. Cluster Validation by Prediction Strength. *J. Comput. Graph. Stat.* **14**, 511–528 (2005).

71. Yin, H. *et al.* A Rapid Review of Clustering Algorithms. *arXiv [cs.LG]* (2024).

Figure 1

Click here to access/download;Figure;Figure 1.pdf

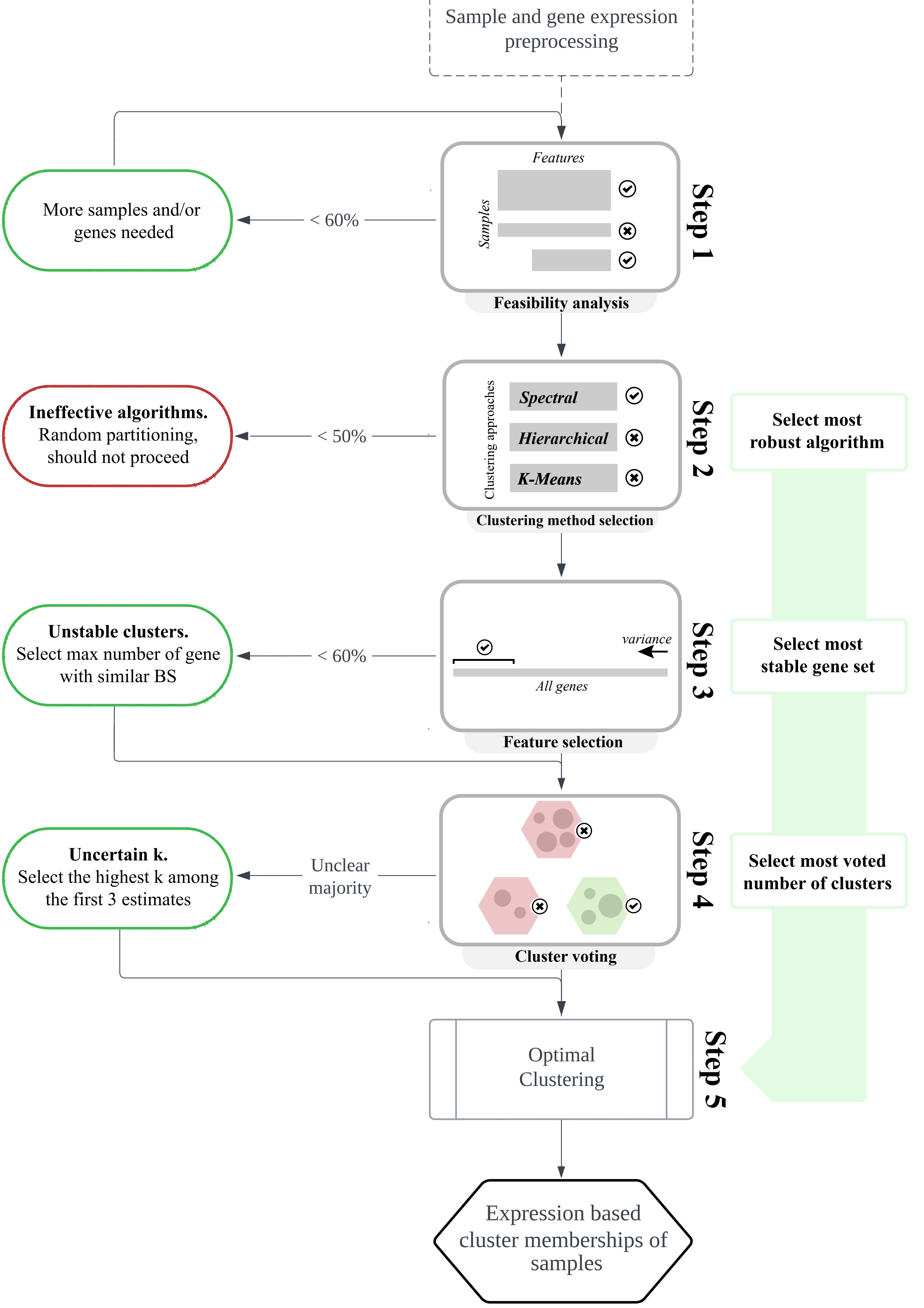

Figure 2

Click here to access/download;Figure;Figure 2.pdf

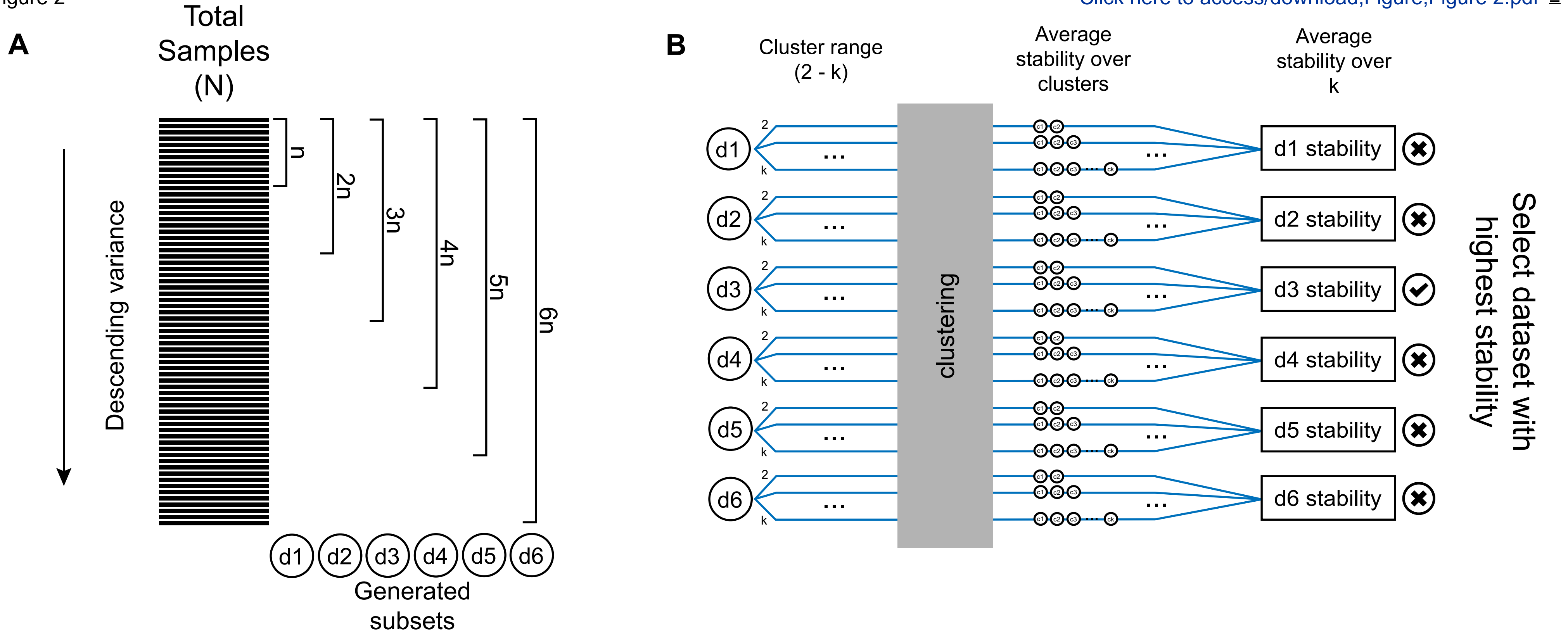

Figure 3

Click here to access/download;Figure;Figure 3.pdf

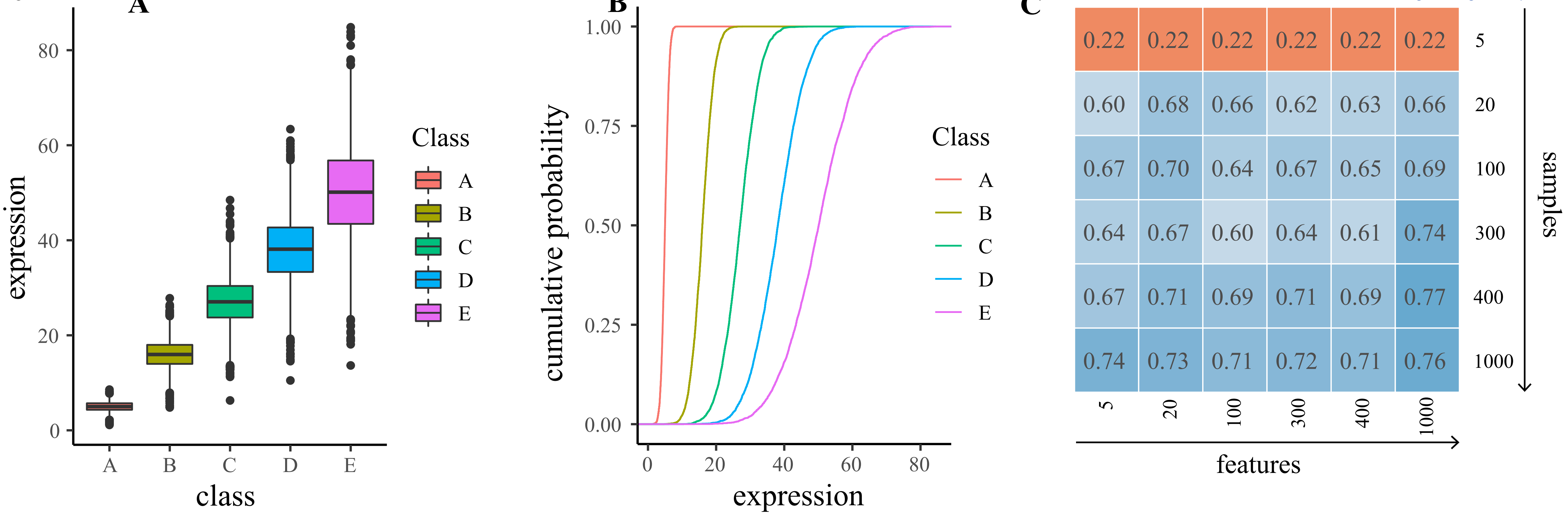

Figure 4

Click here to access/download;Figure;Figure 4.pdf

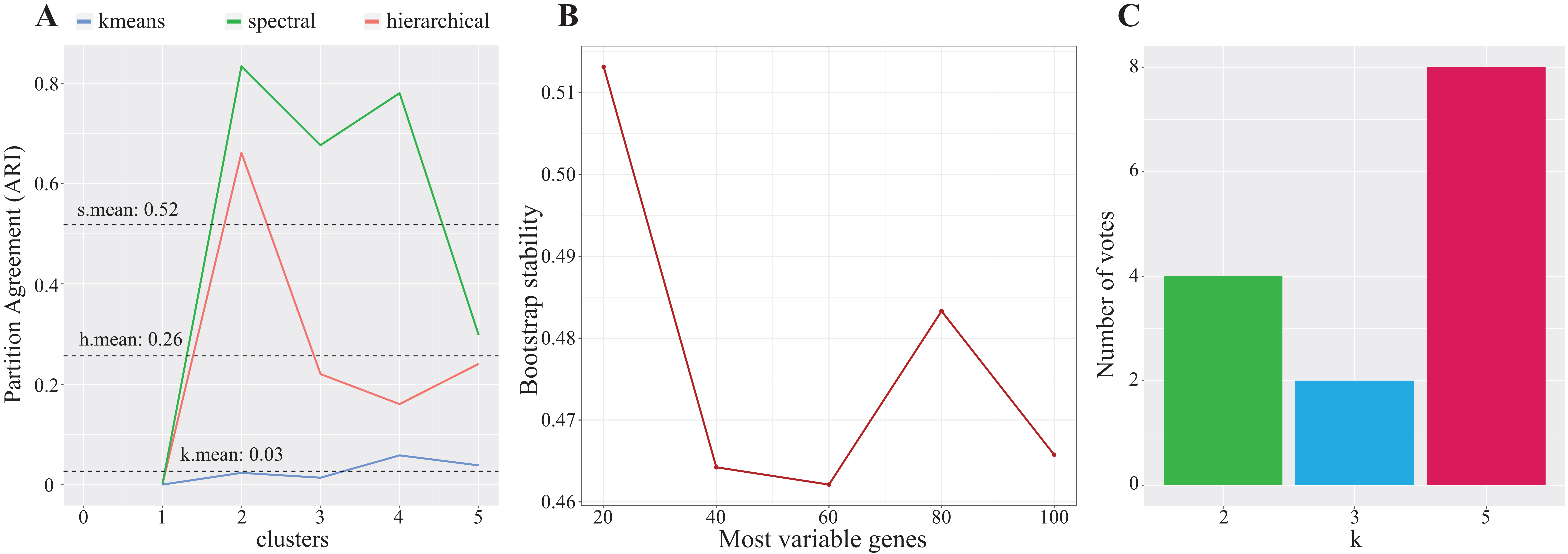

Figure 5
Click here to access/download;Figure;Figure 5.pdf

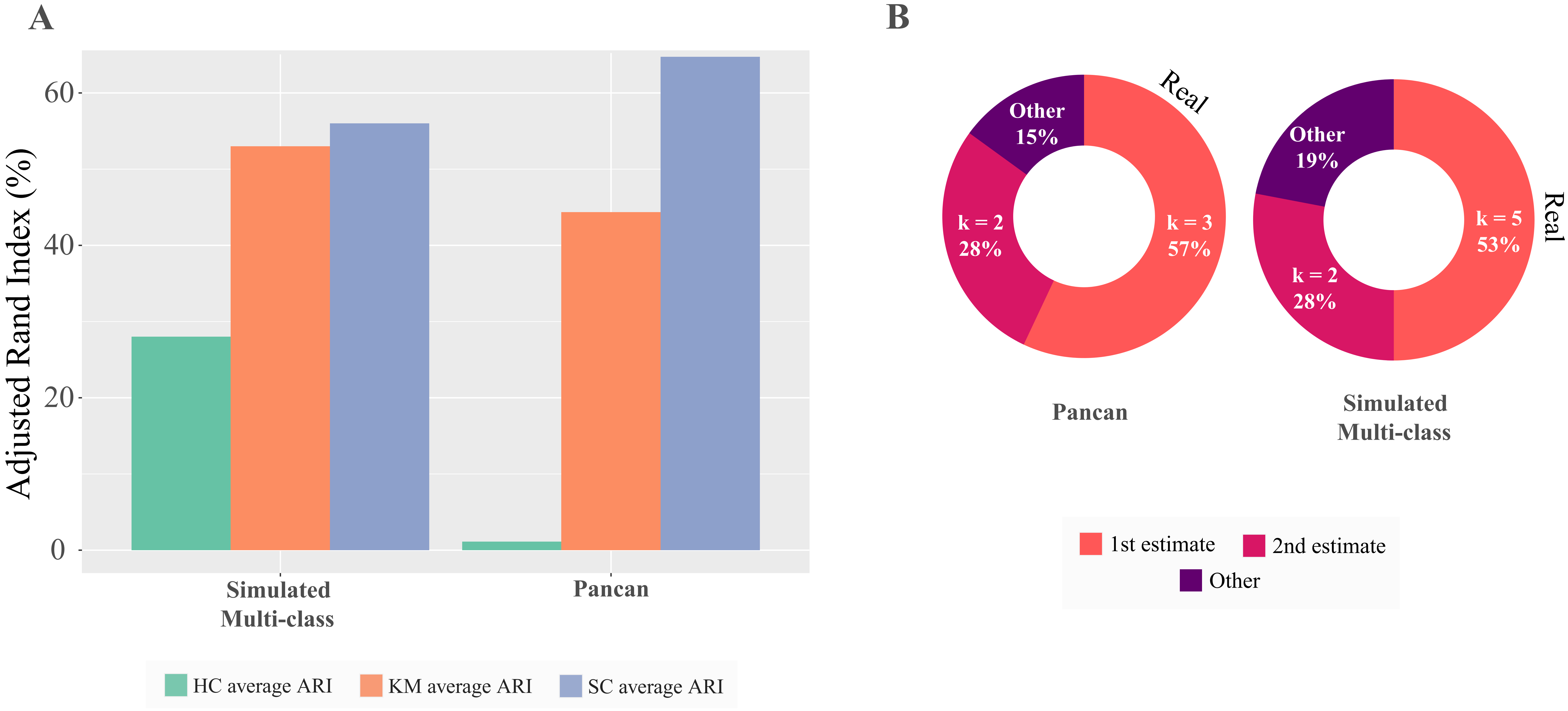

C

Multi-class simulated RNA-seq data

	Class 1	Class 2	Class 3	Class 4	Class 5
Cluster 1	72	0	0	0	0
Cluster 2	0	0	0	72	0
Cluster 3	0	0	72	0	0
Cluster 4	0	0	0	0	72
Cluster 5	0	71	0	0	0

Pancan multi-tissue RNA-seq data

	BRCA	COAD	LUAD
Cluster 1	0	0	503
Cluster 2	1084	3	63
Cluster 3	0	591	

Click here to access/download;Figure;Figure 6.pdf

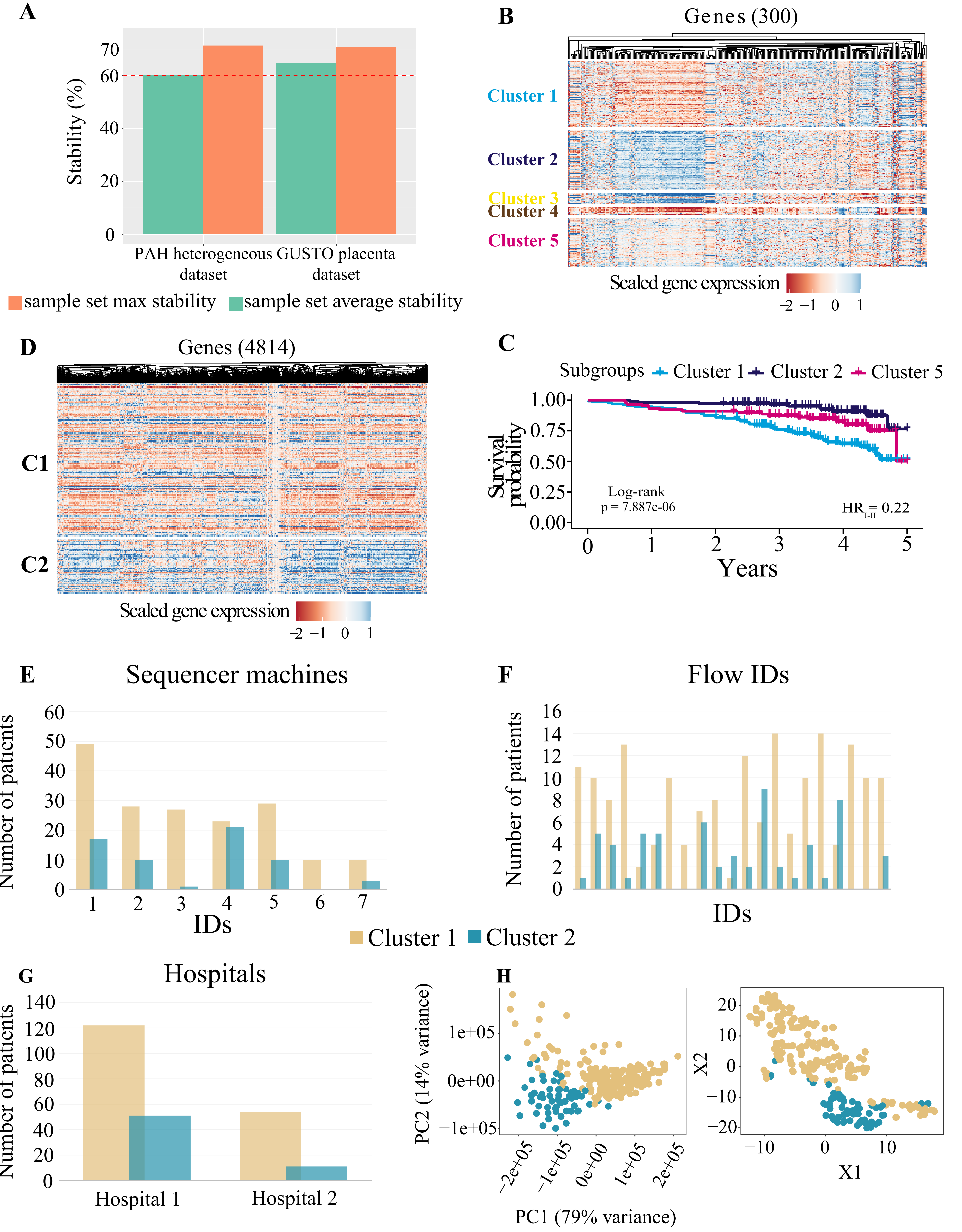

Click here to access/download
Supplementary Material
Omada Supplementary V3.pdf

Personal Cover

Click here to access/download;Personal Cover;Response to reviewer.pdf

Dear Editor,

Thank you for the opportunity to respond to the single reviewer's comments. We have addressed all their comments and made extensive edits to the text to satisfy the reviewer's preferences for writing style. These include:

- Substantial re-writing of the Introduction and Discussion to present the paper more as an article describing a software package.
- More details in all sections about the purpose, rationale behind parameter choices, benchmarking across multiple different applications, and discussion about context.
- Additional functions for consensus clustering and metric for measuring agreement between multiple clustering outputs
- Additional analysis of cancer data showing agreement between clustering algorithms and parameters.

We hope these extensive amendments will satisfy your criteria for publication in Gigascience.

Best regards,

Dennis Wang

(on behalf of all co-authors)

Reviewer Comment: I found the manuscript difficult to read. It reads somewhat like a how-to guide and somewhat like a software package. I recommend approaching this as a software package, which would require adding evidence to support the choices made. Describe the purpose for the package, evidence for the choices made, benchmarking (compute and performance), describe application to one or more case studies, and discuss how the work fits into the context.

Response: Thank you for the honest feedback on the readability of our paper. We have now made substantial revisions to the *Introduction* and *Discussion* sections to describe our aims and conclusions for Omada as a software package.

We want to stress that Omada's objective is not to provide superior clustering algorithms compared to other methods. Instead, we aim to highlight that Omada is a toolkit equipped with various functions, metrics, and algorithms to assess datasets suitability for partitioning to the formation of clusters. It is not just about offering metrics and algorithms for selection; rather, our approach is designed to help both experienced and inexperienced users achieve justifiable clustering outputs. We also provide a number of test datasets (some previously unpublished) for users to explore different parameters and how they perform in datasets from different health conditions and tissues.

Introduction

The rapid development of next-generation sequencing boosted the quantitative analysis of gene expression in a variety of human tissues and organs[1,2] generating valuable resources[3] for downstream investigative analysis. In recent years, such analyses aim to elucidate disease mechanisms[4] and construct genomic profiles[5] to explain diagnosis[6], prognosis and treatment patterns. However, transcriptomic profiles can be heterogeneous due to several causes pertaining to technical biases that produce batch effects[7], cellular diversity[8], disease heterogeneity[9] as well as differences between individuals and populations[10,11]. In turn, this heterogeneity hinders research efforts aiming to identify subpopulations within complex diseases by applying unsupervised machine learning techniques[12]. More specifically, clustering algorithms have been applied to high-dimensional data from transcriptomic profiling[13–15], and revealed novel molecular classes associated with different symptoms of disease[16–18]. Despite the intrinsic capabilities of these algorithms, a recurrent challenge is the insufficiency of default configurations to tailor these models precisely to unique datasets, thereby impeding the extraction of critical insights into the heterogeneity of the samples. This limitation underscores the complexity and heterogeneity inherent in biological data, which often eludes a one-size-fits-all approach to clustering. Empirical evidence from numerous comparisons and tests reveals that the algorithms exhibit variable efficacy across different datasets and disease contexts, accentuating the necessity for model tuning or optimization specific to the dataset in question, particularly with advanced methodologies[19,20,21,22]. The challenge is further compounded by the possibility that the samples could be from a spectrum of states and there are no optimal metrics to group them.

Most clustering packages offer implementations of one or more algorithms without considering the previous and subsequent steps of the clustering process, such as detecting useful features or estimating the number of subgroups in the data. Since clustering consists of multiple steps, it is important to carefully approach each step so that intermediate decisions are not arbitrary but based on appropriate clustering theory. Due to this diversity in methodologies, there is a need for a toolkit that guides non-expert users through the different parameters of common clustering algorithms for their unsupervised analysis. One of the most important aspects of sample partitioning is the stability of the generated groups as unstable clusters, usually imply the lack of signal which should be

present and drive the clustering of samples. Signals from genes across the transcriptome can vary across different tissues and diseases. Cluster instability can be caused inherently by the data points or by the type and application quality of a clustering technique. There lacks a robust approach that incorporates comprehensive assessment of multiple clustering methods and parameters for different sources of transcriptomic data.

Here we introduce Omada, a toolkit with multiple functions based on cluster stability and quality metrics that supports both experienced and inexperienced users from dataset exploration to the formation of the sample clusters. In contrast to other methodologies, we provided a flexible toolkit offering multiple metrics and algorithms for testing, as well as an end-to-end pipeline. Synthetic and real-world datasets from bulk tumour, single cell sequencing and whole blood have been used to test the general usability of Omada. While we cannot guarantee finding the optimal parameters, especially for healthy test samples where true clusters are unknown, we aim to provide users with guidelines based on quality metrics and empirical best practices, in order to ensure that their clustering results are robust and well justified.

Discussion

Omada is designed to enable the assessment of multiple clustering solutions arising from transcriptomic studies of human samples. With the plurality of unsupervised methods available and their specialised nature, the selection of the most appropriate approach is a multi-factor problem and suggesting a general system of recommendation is impossible without considering each dataset in advance21. Many decisions on technical parameters are required from the user of clustering approaches in order to get a meaningful set of subgroups 71. To assist with this problem, our toolkit initially assesses the potential of a target dataset and provides recommendation for the most appropriate clustering algorithm, gene set and number of possible subgroups. Omada can also be run as a pipeline, therefore, does not require deep prior knowledge of the algorithms, parameters and metrics by the user. All outputs of the pipeline, intermediate and final, are observable and each step is justified by multiple measures and indices representing widely used clustering techniques. We showed that a user of Omada can conduct analysis to assess the feasibility of applying different clustering algorithms and parameters to different RNAseq datasets from healthy and diseased tissue samples.

Conducting unsupervised learning of expression datasets is often not a straightforward task as the true heterogeneity of the health condition or tissue of origin is unknown. No methods or metrics can give a definitive answer without validation from external biological or clinical insights, therefore, the output of each algorithm has to be used with caution. Determining the dataset clustering potential is an indication rather than a clear sign that partitioning the dataset will yield informational subgroups71. Additionally, clustering can often contain non-deterministic steps (i.e. k-means initial cluster centers selection40) allowing for each function to behave slightly differently between similar runs. In order to evaluate the uncertainty and provide a robust set of tools, Omada has been applied to gene expression datasets where we have prior confidence in the number of clusters (simulated and cancer type data) and where we have little prior knowledge (GUSTO whole blood, PAH). Even in data from healthy tissue, such as from GUSTO whole blood, small technical biases can result in prominent clusters detected by Omada. It is also possible for Omada to hint towards the existence of a single homogeneous cluster by consistently revealing low partition agreement and stability scores across multiple functions, as demonstrated in our single-class datasets. Furthermore, Omada can help in selecting a small group of genes with potential partitioning capabilities as the feature selection step is expected to greatly reduce the number of genes which in most cases count to thousands.

This toolkit currently encompasses common clustering techniques, metrics and test datasets, but its modular nature allows its extension to more specific clustering problems, such as for single cell and spatial data. Furthermore, the structure of this toolkit allows for additional algorithms and metrics to be added to the pool of clustering methods to be evaluated during an analysis run. Systematic empirical testing of algorithms using Omada's approach may result in more robust and better justified clustering outputs for a variety of biomedical studies.

We would also like to highlight benchmarking of compute time and scalability performance which was added in the last revision. In the “Test datasets” section, we added the data processing steps of PBMC single-cell use case.

In the methods section, we added a new section “Evaluation of run time and memory usage”. A new section “Scalability Performances” in the result section was also added to report how Omada scales in terms of runtime and memory for simulated datasets of different sizes for both bulk RNA-seq and single-cell data use cases.

In addition to scalability performance, we also performed the benchmarking of Omada against other clustering methods and added the section “Benchmarking Omada against other clustering approaches”. Furthermore, benchmarking was also performed on a high-dimensional single-cell use case where a random subset of 4 major celltype including monocytes, B cells and CD4 and CD8 T cells consisting of 1000 cells and 10000 features/genes were analysed.

To compare Omada’s results to previous clustering results from cancer studies, we utilise an additional TCGA LUAD dataset from PMID 35664309. With this, we added additional guidelines on the data processing step. We added the results section under “Discriminating cancer types from pan cancer tissue expression data”.

Reviewer comment: The evaluation includes two simulation studies and then application to a few real datasets; however, for all real datasets the problem is either very easy or the answer is unknown. The largest challenges I have with the manuscript are the large number of arbitrarily selected parameters the limited evidence available to support those as strong choices.

We understand that real datasets can vary in complexity, ranging from being straightforward to having unknown answers. However, when the cluster memberships are known, such as in the case of two simulated datasets (single-class or multi-class with 5 classes), the Omada pipeline can be automated efficiently, and validation is trivial.

Conversely, in most datasets from human tissue, as seen in our data from pulmonary hypertension and maternal blood, descriptive statistics and parameters from the Omada pipeline need to be outputted and evaluated by the user in order to estimate what might be the true clusters. The user would need prior knowledge about the health conditions and other phenotypic information from the donors.

To provide more evidence on the choices made in our example analyses, we have made the following amendments.

Under “Sample and gene expression preprocessing”, we adjusted the following text:

Various normalisations can be utilised, each catering for specific data types (i.e. arcsine transformation fit RNA-seq data, as described in section “Test datasets”). It's worth noting that Omada applies the arcsine transformation on the input data during the first step and the transformed data are carried over to subsequent steps.

In addition, we added data processing guidelines for the PBMC single-cell dataset use case:

Additionally, high-dimensional data such as the single-cell transcriptomic data should be properly quality-controlled where dying (or non-viable) cells and cells with empty droplets or multiplets should be removed. Together with normalisation, scaling, clustering and cell type specific quality control, such data pre-processing steps ensured high quality data to be considered for further analysis.

For stability based analysis, we added the following:

As for the number of genes, it is advised for larger genesets (>1000 genes) to filter down to the most variable ones (few hundreds) before the application of any function as genes that do not vary across samples do not contribute towards identifying heterogeneity. In this step the filtering should not be very strict but rather discard genes with zero or near zero variance (i.e. housekeeping genes). In Omada, the main filtering is achieved in a later stage by identifying the set of genes that outputs the highest stability of generated clusters, as described in section "Stability-based assessment of feature sets"

On "Determining clustering potential", we added the following text:

At the start of each study, we assess the suitability of the input dataset for clustering, using the Omada package pre-pipeline analysis, to ensure general dataset attributes do not influence the process (Figure 1). The number of samples and features (e.g. genes), as well as the balance of the two dimensions, directly affects the capabilities of clustering methods to handle the dataset. An inadequate number of samples does not provide enough training power[44], while an overabundance of samples might clutter the provided information and confuse most methodologies[45]. Similarly, too few features can lead to weak clustering criteria and too many features might lead a methodology away from the features that can really differentiate between clusters of samples. Therefore, to estimate the feasibility of a clustering procedure on a specifically sized dataset we rely on measurable metrics of cluster quality, such as stability. Clusters of high stability denote both a partitionable dataset as well as a dataset-suitable methodology[46]. The feasibility score of any dataset is a function of both dimensions as well as the number of classes requested. As such, if too many or a single class is requested of a relatively small dataset the calculation will reflect low feasibility due to insufficient samples and/or features to form the desired classes. Omada's functions feasibilityAnalysis() and feasibility_analysis_based_on_data.R, can simulate datasets with dimensions similar to any dataset and calculate their stability across different numbers of clusters to determine whether such a dataset can be stable enough to be considered for clustering.

Under "Intra-method Clustering Agreement" section, we included

We implemented a tool (Figure 1) to calculate an average agreement score per clustering approach by comparing a number of runs within each of the three clustering approaches (hierarchical[47], k-means[48], spectral clustering[49]) using multiple parameters (kernels, measures, algorithms) specifically based on the data set provided which is already arcsine transformed from the first pipeline step. The aforementioned clustering methods were selected to represent fundamentally different approaches of partitioning data so as to cover several types of datasets. Hierarchical clustering is well suited for hierarchical data or data that can be represented as dendrograms, such as taxonomies

(Rodriguez et al. 2019). K-means allows a different approach of partitioning samples, namely Centroid-based clustering, which is based on non-hierarchical clusters, vector quantization and representing clusters with their centroids (Rodriguez et al. 2019). Spectral clustering adds a spatial component to k-means as it utilises a similarity matrix to create a graph where clusters with irregular shapes are investigated (Rodriguez et al. 2019). Although the above approaches are not exhaustive, the modular nature of Omada allows for additional approaches to be added in subsequent versions.

We also adjusted the following text to address comparison between runs.

The number of comparisons (c), between runs of the same approach, is an additional overarching parameter of this tool and contributes to the agreement score. Each comparison requires two variations of the same algorithm. These variations are expressed as clustering parameters such as distance measures or in the case of hierarchical clustering linkage type (Table 2). A difference in such parameters produces clustering models that despite using the same algorithm have different formulas to calculate clusters. If these different formulas agree on how to partition the data then the algorithm using them demonstrates that it can capture an underlying signal in the data rather than randomly distributing data points. For each comparison, the parameters of the two runs are drawn randomly from a predefined set (Table 2) selected randomly with replacement while not allowing the same parameters to be used within one comparison. As we have to decide on a dataset without prior training on similar data, the random choice of multiple parameters is more reliable than selecting any single parameter. Additionally, in the interest of performance and computational time we suggest three comparisons to be used instead of exhaustively using every parameter.

Under “spectral clustering algorithm”, we added details to the stepwise method.

Given a set of points $S = \{s_1 \ldots ,s_n\}$ in R^l that we want to cluster into **k** subsets:

1. Form the affinity matrix A∈ Rn✖n defined by Aij = exp(-||si - sj||2 /2σ2) if i ≠ j , and Aii = 0
2. Define D to be the diagonal matrix (where A(i,j) = 0) whose (i, i)-element is the sum of A’s i-th row, and construct the matrix $L = D^{-l/2}AD^{-l/2}$
3. Find x_1 , x_2 , ... , x_k , the k largest eigenvectors of L (chosen to be orthogonal to each other in the case of repeated eigenvalues), and form the matrix X = $[x_1\, x_2 \ldots x_k] \in {}^{n✖k}$ by stacking the eigenvectors in columns
4. Form the matrix Y from X by renormalizing each of X's rows to have unit length (i.e. $Y_{ij} = X_{ij} / (\Sigma_j X^2_{ij})^{1/2}$)
5. Treating each row of Y as a point in R^k , cluster them into k clusters via K-means or any other algorithm (that attempts to minimize distortion)
6. Finally, assign the original point s_i to cluster j if and only if row i of the matrix Y was assigned to cluster j

---

Under “hierarchical clustering algorithm” we added the following:

Hierarchical clustering algorithm (average linkage)

Given a set of points S = {s_1 ... ,s_n} that we want to cluster into **k** subsets:

1. Initialize with n clusters, each containing one data point (s_i)
2. Compute the between-cluster distance D(r, s) as the between-object distance of the two data points in clusters r and s respectively, r, s =1, 2, ..., n. Let the square matrix D = (D(r, s)). Various distances can be used (euclidean, manhattan, canberra, minkowski, maximum).
3. Find the most similar pair of clusters r and s, such that D(r, s) is minimum among all pairwise distances
4. Merge r and s to a new cluster t and compute the between-cluster distance D(t, k) for any existing cluster k ≠ r, s. Once the distances are obtained, delete the rows and columns corresponding to the old cluster r and s in the D matrix, as r and s do not exist anymore. Then add a new row and column in D corresponding to cluster t.
5. Repeat Step 3 a total of n − 1 times until there is only one cluster left (effectively minimising the number of redundant clusters).
6. Decide on a point to cut the cluster tree created above so as to obtain the desirable number of clusters (k)

In the “Stability-based assessment of feature sets” section, we added

To assess the suitability of each resampled feature set for our clustering, we measure the average stability of the clusters they generate per run when a clustering method is applied over a range of k’s (Figure 2B). First, the clustering range, where the stability of each dataset will be calculated, is selected. The lower end of this range is 2, the minimum possible number of clusters and the upper case is selected by the user. For each dataset we generate the bootstrap stability for every k within range (clusterboot function in R package fpc v2.2-3 using nonparametric bootstrap, spectral clustering and 25 resampling runs).

In the “Choosing k number of clusters”, we added the following text.

Most clustering methods require the number of k clusters to be defined as a parameter before the application of the algorithm on the data. The lack of a concrete way to determine the real number of clusters in a dataset led many studies to base their estimation on field/prior knowledge or various estimation methods such as the Silhouette score[52]. However, each method favours different aspects of the generated clusters (i.e.

how compact clusters are and how far apart cluster centres are) and therefore suits specific datasets and may introduce bias towards the selection of k. To encompass these different angles in one methodology, avoid the risk of selecting an ineffective index and present a more general solution, this tool uses an ensemble learning approach (Figure 1) where multiple internal cluster indexes contribute to the decision making[53]. This approach prevents any bias from specific metrics and frees the user from making decisions on any specific metric and assumptions on the optimal number of clusters.

Initially, the value of the 15 indexes is calculated for each k within a cluster range of $[2, x]$, where x is a logical upper limit of the number of clusters realistic for our dataset (i.e. the present conditions within a dataset). The means over k are calculated per index and the optimal k is estimated by majority voting of the 14 means that evaluate the compactness and/or the distance between different subgroups. The selection of indexes can be found in Table 3. It is important to note that the most important aspect of determining k is minimum loss of information which directs us to overestimate and not underestimate k[51] while interpreting the voting results. Furthermore, cases that present only a single k as the optimal number of clusters should be treated with caution in case they are a result of a biased dataset. Omada scores each k within the set range [2, x] enabling the user to observe the most probable number of clusters according to the above criteria (function clusterVoting()). This prediction is agnostic to any other type of biological information except gene expression. However, after the clustering groups are formed it is advised for the user to investigate whether they represent relevant to the specific case biological subgroups."

Reviewer Comment: Conceptually, an alternative strategy is to consider real clusters to be those that are robust over many clustering methods. In this case, the best clusters are those that are maximally detectable with a single method. While there exists software for the former strategy, this package implements the latter strategy. It is not intuitively clear to me that this framework is superior to the other for biological discovery. It seems like general clusters (i.e., those that persist across multiple parameterizations) may be the most fruitful to pursue. It would be helpful to provide evidence that the selected strategy has superior utility in at least some settings and a description of how those settings might be identified. It is possible this is not necessary, but I simply note it as I continue to have these challenges with the revised manuscript.

We agree that examining multiple clustering outputs and finding their agreement could be an effective strategy towards finding true cluster memberships. Since there is no standard way of aggregating multiple clustering memberships and reaching a consensus that guarantees the most robust clusters, we left it to the users to subjectively decide. Users of Omada are able to stop at each step of the pipeline and compare parameters, but we have now added functions for them to get the partition agreement between different clustering outputs and parameters tested.

```
# Selecting algorithms, measures and number of clusters
agreement.results <- partitionAgreement(toy_genes, algorithm.1 = "spectral",
                                        measure.1 = "rbfdot",
                                        algorithm.2 = "kmeans",
                                        measure.2 = "Lloyd",
                                        number.of.clusters = 3)

# Extract results
pa.scores <- get_agreement_scores(agreement.results)
```

We have also added the option for users to conduct consensus clustering using M3C to find the clusters that persist across multiple parameterizations, as you have suggested. We caution them to still use human judgement by showing an example of clustering cancer types where there may not be a single clustering run that generates the most likely number of clusters.

Methods

Exploring the number of clusters (k) when using single algorithms versus consensus clustering

In this section we test whether, in RNA-seq clustering tasks, a single clustering algorithm can be a superior choice than other algorithms which represent different approaches. Towards this goal we extract gene expression datasets from the Cancer Genomics cBioPortal (https://www.cbioportal.org/) and test the extent to which different algorithms can recognise different cancer types. The first dataset consists of three cancer types: Esophageal Adenocarcinoma (esca, n = 181) , Thymoma (thym, n = 119) and Testicular Germ Cell Tumors (tgct, n = 149). The 20,531 genes were first filtered to exclude duplicates as well as those without expression values for the majority of samples. They were subsequently ranked, in a descending manner, for variance across samples and then 500 most variant genes were included in the clustering models. The dataset was normalised using the inverse hyperbolic sine (function asinh in base R package). The second dataset was simulated based on exact dimensions of the aforementioned cancer dataset using Omada's simulation function feasibilityAnalysisDataBased() with 3 distinct classes. Spectral, kmeans and hierarchical clustering algorithms were tested alongside a consensus clustering method M3C from package https://www.bioconductor.org/packages/release/bioc/html/M3C.html. Default parameters were used for each algorithm. The adjusted Rand index was calculated between the set of generated memberships of the top k estimate and the true cancer type labels of the samples. Additionally, we registered the k estimation of each methodology against the real number of different cancer types for each dataset. To estimate k for hierarchical, spectral and k-means the Omada ensemble method was used, while for M3C the provided Relative Cluster Stability Index (RCSI).

Results

Not all algorithms can detect the true number of clusters

We tested the ability of four algorithms to cluster samples that belong to three cancer types. As seen on Table X1, hierarchical clustering was not able to identify the three cancer types (ARI = 0.55) as it selected two clusters as most probable. On the contrary, spectral clustering separated the samples most accurately (ARI = 0.83) with a clear preference for three clusters (8/14 votes) closely followed by k-means (ARI = 0.80 and 5/14 votes). Consensus clustering followed with an ARI of 0.78 but a widest spread of estimated k as it selected 5 and 6 clusters as second and third choices respectively. On the simulated dataset (Table X2), k-means estimated the correct number of clusters and managed to assign the memberships perfectly (ARI = 1). All other algorithms selected a different k and therefore presented a much lower ARI (as comparing a set of two clusters with a set of three clusters cannot result in a high ARI). Both Omada and consensus clustering M3C suggest k = 3 as the second estimate. However, Omada already supplies the memberships for k = 3 as part of the k estimation achieving a perfect ARI of 1 if that partitioning is selected for all three algorithms.

Table 4: The ARI of the top k estimate of various datasets with different numbers of cancer types

**Algorithm**	**Real clusters**		
	**3**	**5**	**7**
**Hierarchical**	0.55 (k=2)	0.13 (k=2)	0.19 (k=2)
**k-means**	0.80 (k=3)	0.54 (k=4)	0.45 (k=)
**Spectral**	0.83 (k=3)	0.84 (k=5)	0.58 (k=)
**M3C**	0.78 (k=3)	0.85 (k=5)	0.63 (k=9)

Table 5: The ARI of the top k estimate of various datasets with different numbers of simulated classes

**Algorithm**	**Real clusters**		
	**3**	**5**	**7**
**Hierarchical**	0.57 (k=2)	0.9 (k=7)	0.9 (k=9)
**k-means**	1.00 (k=3)	1.00 (k=5)	0.79 (k=8)
**Spectral**	0.57 (k=2)	1.00 (k=5)	1.00 (k=7)
**M3C**	0.57 (k=2)	0.78 (k=5)	0.54 (k=4)
