# Supplementary material for: Omada: robust clustering of transcriptomes through multiple testing
Source: Gigascience. 2024 Jul 11;13:giae039. doi: 10.1093/gigascience/giae039 (PMC11238428; doi:10.1093/gigascience/giae039)
Supplement: giae039_Supplemental_File [file giae039_supplemental_file.pdf]

Omada: An unsupervised machine learning toolkit for automated sample clustering of gene expression profiles

Kariotis *et al*

Supplementary results

To determine the significance of the difference between simulated distributions, used during the dataset simulation, the Kolmogorov's D statistics are shown in Supplementary Table 1.

Supplementary results

Datasets

All results generated from our tools application on the available datasets can be found in Supplementary Table 2 (simulated single-class dataset), Supplementary Table 4 (simulated multi-class dataset), Supplementary Table 5 (pancan dataset), Supplementary Table 7 (I/PAH dataset) and Supplementary Table 8 (GUSTO dataset). Additionally, the confusion matrix for the simulated multi-class dataset, for which we know the actual labels, are presented in Supplementary Table 6.

Supplementary Tables

Supplementary Table 1 | Simulated single-class dataset results, composed of 100 samples and 100 genes drawn from a single distribution

Tool	Parameters	Results
Dataset clustering feasibility	100 samples, 100 genes	max stability = 0.55<br>average stability = 0.45
Clustering method selection	max clusters = 6<br>comparisons = 3	Spectral PA = **0.52**<br>Kmeans PA = 0.03<br>Hierarchical PA = 0.26
Sample set selection	min(k) = 2<br>max(k) = 6<br>feature step = 20	Optimal features = 20<br>max stability = 0.51<br>average stability = 0.47
K estimation	min(k) = 2<br>max(k) = 6	Optimal k = 5

	method = spectral	

**max clusters: the maximum number of clusters to be tested starting from 2, range [2, max clusters]*
**min(k): minimum number of clusters to be tested*
**max(k): maximum number of clusters to be tested*
**feature step: the number of features by which the generated datasets grow. Also, the smallest dataset to be tested*

Supplementary Table 2 | Kolmogorov's D statistic for a simulated dataset containing 5 clusters (A, B, C, D, E). Each distance D has a p-value of less than 2.2e-16

A vs B	A vs C	A vs D	A vs E	B vs C	B vs D	B vs E	C vs D	C vs E	D vs E
0.99	0.99	1	1	0.84	0.97	0.97	0.65	0.88	0.54

Supplementary Table 3 | The scores of all internal indexes used to decide on the ensemble voting of the number of clusters for the multi and single class simulated datasets. The scores for the most voted k are presented for each dataset along with the ideal score (min/max) for each index

	Multi Class (k=5)	One Class (k=6)	Ideal
**Calinski-Harabasz**	608.5824	5.325228	max
**Dunn**	0.672101	0.373477	max
**Pbm**	740.0956	0.709396	max
**Tau**	0.50501	0.192052	max
**Gamma**	0.896494	0.335348	max
**C index**	0.01731	0.324603	min
**Davies–Bouldin**	0.993194	2.897971	min
**Mcclain rao**	0.303852	0.907469	min
**sd_dis**	0.233813	0.479863	min
**Ray–Turi**	0.301727	2.20407	min
**g_plus**	0.016422	0.108995	min
**Silhouette**	0.479414	0.050476	max
**s_dbw**	0	0	min
**Compact ness**	0	145.1075	max
**Connecti vity**	7.512624	6.277666	max

Supplementary Table 4 | Simulated multi-class dataset results, where distribution is represented by around 120 samples and 3 clusters are used as a default parameter

Tool	Parameters	Results

Dataset clustering feasibility	359 samples, 300 genes	max stability = 0.78<br>average stability = 0.72
Clustering method selection	max clusters = 6<br>comparisons = 3	Spectral PA = **0.56**<br>Kmeans PA = 0.53<br>Hierarchical PA = 0.28
Sample set selection	min(k) = 2<br>max(k) = 6<br>feature step = 25	Optimal features = 300<br>max stability = 0.84<br>average stability = 0.78
K estimation	min(k) = 2<br>max(k) = 6<br>method = spectral	Optimal k = 5

**max clusters: the maximum number of clusters to be tested starting from 2, range [2, max clusters]*
**min(k): minimum number of clusters to be tested*
**max(k): maximum number of clusters to be tested*
**feature step: the number of features by which the generated datasets grow. Also, the smallest dataset to be tested*

Supplementary Table 5 | Pancan multi-tissue RNA-seq dataset results using 3 cancer classes

Tool	Parameters	Results
Dataset clustering feasibility	2244 samples, 243 genes	max stability = 1<br>average stability = 0.88
Clustering method selection	max clusters = 5<br>comparisons = 3	Spectral PA = **0.63**<br>Kmeans PA = 0.44<br>Hierarchical PA = 0.06
Sample set selection	min(k) = 2<br>max(k) = 5<br>feature step = 50	Optimal features = 243<br>max stability = 0.96<br>average stability = 0.85
K estimation	min(k) = 2<br>max(k) = 6<br>method = spectral	Optimal k = 3

**max clusters: the maximum number of clusters to be tested starting from 2, range [2, max clusters]*
**min(k): minimum number of clusters to be tested*
**max(k): maximum number of clusters to be tested*
**feature step: the number of features by which the generated datasets grow. Also, the smallest dataset to be tested*

Supplementary Table 6 | RNA-seq iPAH/HPAH dataset results as shown in [7]

Tool	Parameters	Results
Dataset clustering feasibility	359 samples, 25955 features	max stability = 0.74<br>average stability = 0.61
Clustering method selection	max clusters = 10<br>comparisons = 3	Spectral PA = **0.86 (max 0.96)**<br>Kmeans PA = 0.17 (max 0.19)<br>Hierarchical PA = 0.57 (max 0.66)
Sample set selection	min(k) = 2<br>max(k) = 10<br>feature step = 50	Optimal features = 300<br>max stability = 0.73<br>Average stability = 0.61
K estimation	min(k) = 2<br>max(k) = 10<br>method = spectral	Optimal k = 5

**max clusters: the maximum number of clusters to be tested starting from 2, range [2, max clusters]*

**min(k): minimum number of clusters to be tested*
**max(k): maximum number of clusters to be tested*
**feature step: the number of features by which the generated datasets grow. Also, the smallest dataset to be tested*

Supplementary Table 7 | Whole blood RNA-seqs datase from pregnant motherst (GUSTO)

Step	Parameters	Results
Dataset clustering feasibility	238 samples, 24,070 features	max stability = 0.59<br>average stability = 0.56
Clustering method selection	max clusters = 5<br>comparisons = 3	Spectral PA = **0.61 (max 0.93)**<br>Kmeans PA = 0.60 (max 0.91)<br>Hierarchical PA = 0.12 (max 0.27)
Sample set selection	min(k) = 2<br>max(k) = 6<br>feature step = 50	Optimal features = 50<br>max stability = 0.71<br>average stability = 0.65
K estimation	min(k) = 2<br>max(k) = 6<br>method = spectral	Optimal k = 2

**max clusters: the maximum number of clusters to be tested starting from 2, range [2, max clusters]*
**min(k): minimum number of clusters to be tested*
**max(k): maximum number of clusters to be tested*
**feature step: the number of features by which the generated datasets grow. Also, the smallest dataset to be tested*

Supplementary Table 8 | P-values of chi-square statistical and generalised linear regression analysis results for clinical variables and maternal phenotypes from the GUSTO dataset

Variables	Method	p-value
Sequenced Flow (22 flows)	chi-square	2.55E-06
Sequenced machine (7 machines)	chi-square	1.39E-03
Total GWG IOM (bookBMI) (Inadequate/Normal/Excessive)	chi-square	0.063
Hospital (2 locations)	chi-square	0.072
Infant sex	chi-square	0.119
Ethnicity(Chinese/Malay/Indian)	chi-square	0.15
Pre-eclampsia	chi-square	0.153
Average STAI trait	GLM	0.195
ppBMI (under/normal/overweight)	chi-square	0.235
bookingBMI, WHO class(under/normal/overweight)	chi-square	0.235
Average STAI state	GLM	0.25
Average total GWG (bookingweight)	GLM	0.258
Average rate of GWG	GLM	0.294
Average maternal age	GLM	0.299
Rate of GWG IOM (ppBMI) (Inadequate/Normal/Excessive)	chi-square	0.311
Time	chi-square	0.351

before/after 11am		
Average ppBMI	GLM	0.379
Rate of GWG IOM (bookBMI) (Inadequate/Normal/Excessive)	chi-square	0.425
Sequenced location (2 locations)	chi-square	0.468
Full/pre-term	chi-square	0.53
Average bookingpBMI	GLM	0.539
Total GWG IOM (ppBMI) (Inadequate/Normal/Excessive)	chi-square	0.577
Average EPDS	GLM	0.602
Average gestational weeks	GLM	0.604
Average total GWG (ppweight)	GLM	0.655
Average fasting glucose	GLM	0.696
Average 2hr post glucose	GLM	0.762
Mother GDM	chi-square	1

Supplementary Table 9 | Runtimes of Omada applied on simulated datasets with increasing numbers of samples and features

		Features							
	Minutes	20	100	300	500	1000	3000	5000	Average runtime
samples	100	0.3	0.3	0.3	0.3	0.4	0.4	0.6	0.4
	300	1.7	1.6	1.7	1.7	1.6	2.2	2.6	1.9
	500	4.6	4.2	4.5	4.4	4.8	5.3	5.3	4.7
	1000	18.4	18.9	19.5	20.0	20.3	21.7	37.2	22.3
	3000	293.6	281.5	282.5	358.2	280.9	366.8	377.6	320.2
	Average run time	63.7	61.3	61.7	76.9	61.6	79.3	84.7	

Supplementary Table 10 | Memory usage of Omada applied on simulated datasets with increasing numbers of samples and features

		Features							
	Mega bytes	20	100	300	500	1000	3000	5000	Average memory

samples	100	199.0	199.0	199.0	200.0	201.0	205.0	211.0	202.0
	300	199.1	200.0	202.0	202.1	206.4	220.0	228.0	208.2
	500	199.0	201.0	203.0	209.0	214.0	227.0	227.0	211.4
	1000	200.8	202.8	209.4	214.4	220.8	252.6	397.4	242.6
	3000	203.6	205.0	242.8	248.0	264.8	433.2	478.0	296.5
	Average memory	199.5	200.7	203.4	206.4	210.6	226.2	265.9	

Supplementary Table 11 | Runtime of Omada applied on single-cell RNA-seq datasets

		Features								
Samples	Minutes	500	1000	2000	4000	6000	8000	10000	12000	Average run time
	100	0.2	0.2	0.3	0.5	0.9	1.3	1.6	2.0	0.9
	200	0.5	0.5	0.7	1.1	1.6	2.4	3.1	4.1	1.7
	400	1.6	1.6	1.7	3.2	4.7	6.1	7.9	10.2	4.6
	600	3.8	3.5	3.9	6.0	7.1	10.7	17.5	19.2	9.0
	800	7.1	6.4	7.1	10.2	16.9	25.3	30.3	42.9	18.3
	1000	12.1	11.0	12.0	13.9	19.2	25.8	32.1	41.3	20.9
	1200	19.8	17.1	17.1	22.7	28.6	48.7	60.8	81.5	37.0
	1400	30.0	25.3	25.1	30.1	40.4	52.4	60.4	94.5	44.8
	1600	42.4	35.1	35.1	48.4	73.7	88.2	90.6	119.7	66.6
	1800	59.7	48.8	45.7	55.1	62.1	75.0	100.1	123.6	71.3
	2000	80.1	63.5	60.9	69.2	94.3	111.1	141.9	156.9	97.2
	Average run time	23.4	19.4	19.1	23.7	31.8	40.6	49.7	63.3	

Supplementary Table 12 | Memory usage of Omada applied on single-cell RNA-seq datasets

		Features								
	Mega-bytes	500	1000	2000	4000	6000	8000	10000	12000	Average Memory
Samples	100	881	882	883	887	894	894	892	899	889.0
	200	885	885	892	893	897	902	896	899	893.6
	400	891	896	895	923	928	925	914	919	911.4
	600	898	903	904	934	918	948	1020	974	937.4
	800	908	912	912	937	1000	1060	1070	1140	992.4
	1000	915	915	960	932	1000	994	967	979	957.8
	1200	919	940	930	1010	957	1160	1160	1280	1044.5
	1400	926	937	992	981	1090	1150	1000	1260	1042.0
	1600	945	953	978	996	1170	1190	1020	1220	1059.0
	1800	940	945	990	1050	997	1020	1040	1060	1005.3
	2000	947	962	983	986.1	1130	1110	1150	1080	1043.5
	Average Memory	914.1	920.9	938.1	957.2	998.3	1032.1	1011.7	1064.5	

Supplementary Table 13 | Partitioning agreement (adjusted Rand Index) between Omada and three clustering algorithms

**k**	**Hierarchical**	**k-means**	**SOM**	**AP**
3	0.994	0.997	0.994	0.4804306

**All algorithm parameters were tuned as described in PMID 31805048*

Supplementary Table 14 | Partitioning agreement (adjusted Rand Index) between each clustering method and the real cancer types

**Omada**	**Hierarchical**	**k-means**	**SOM**	**AP**
0.857	0.862	0.854	0.852	0.373

Supplementary Figures

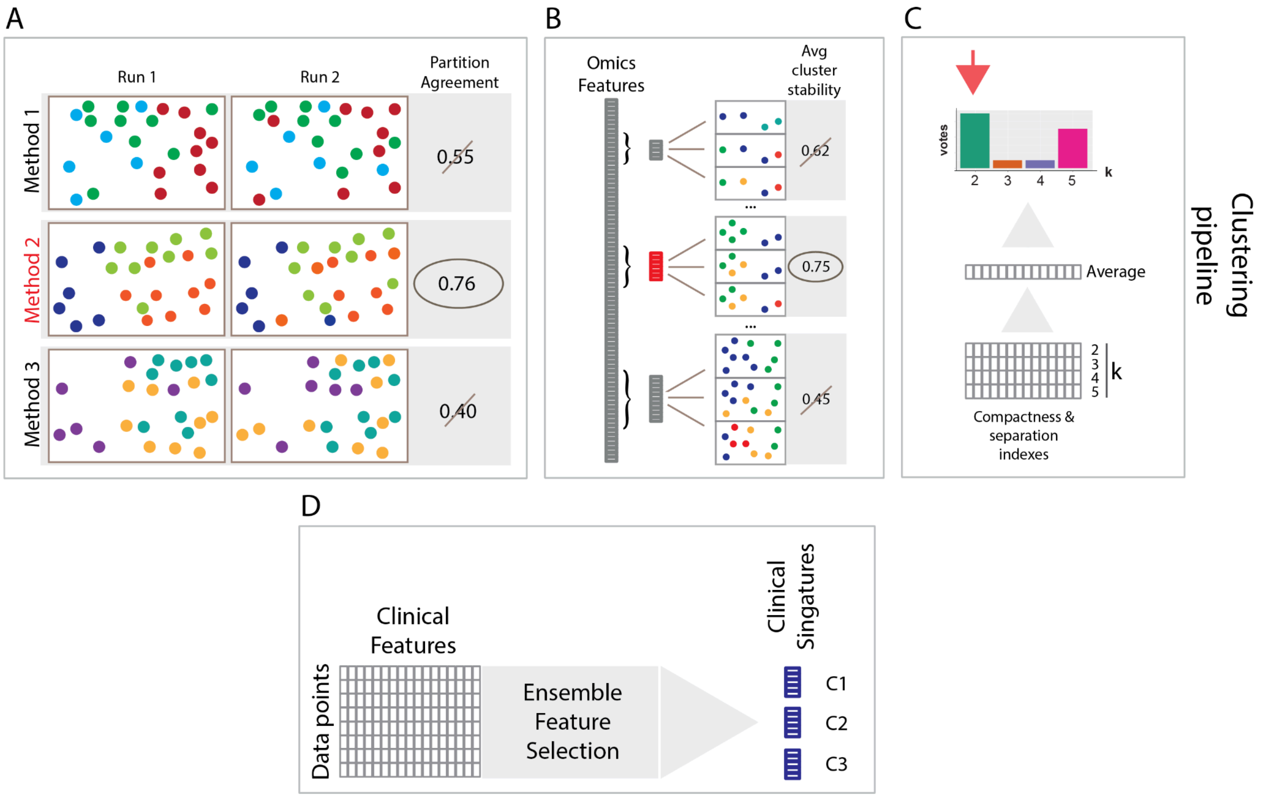

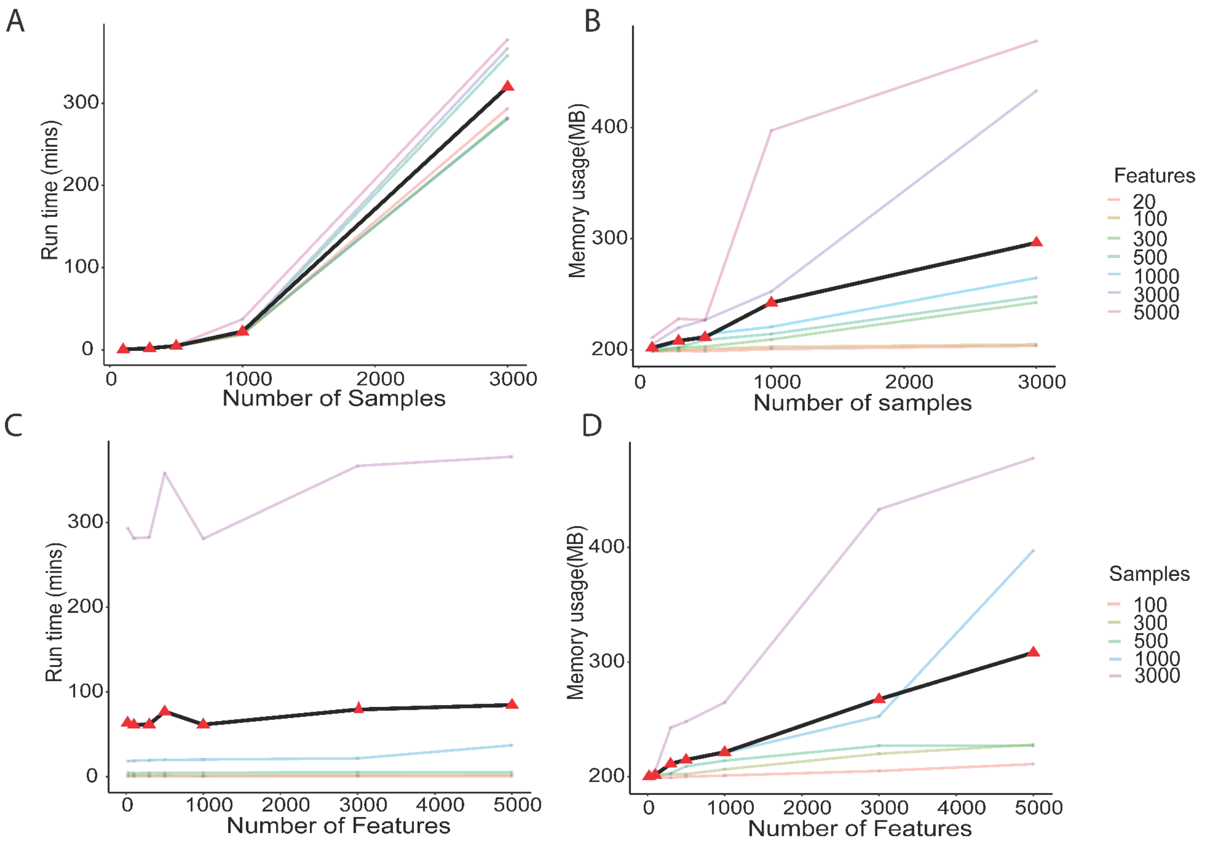

Supplementary Figure 2: Evaluating scalability performance across different scenarios on multiple simulated datasets: (A) measuring run time and (B) memory usage as the number of samples per features vary, and (C) measuring run time and (D) memory usage as the number of features per samples vary. Black line represents average run time and memory usage across samples or across features.

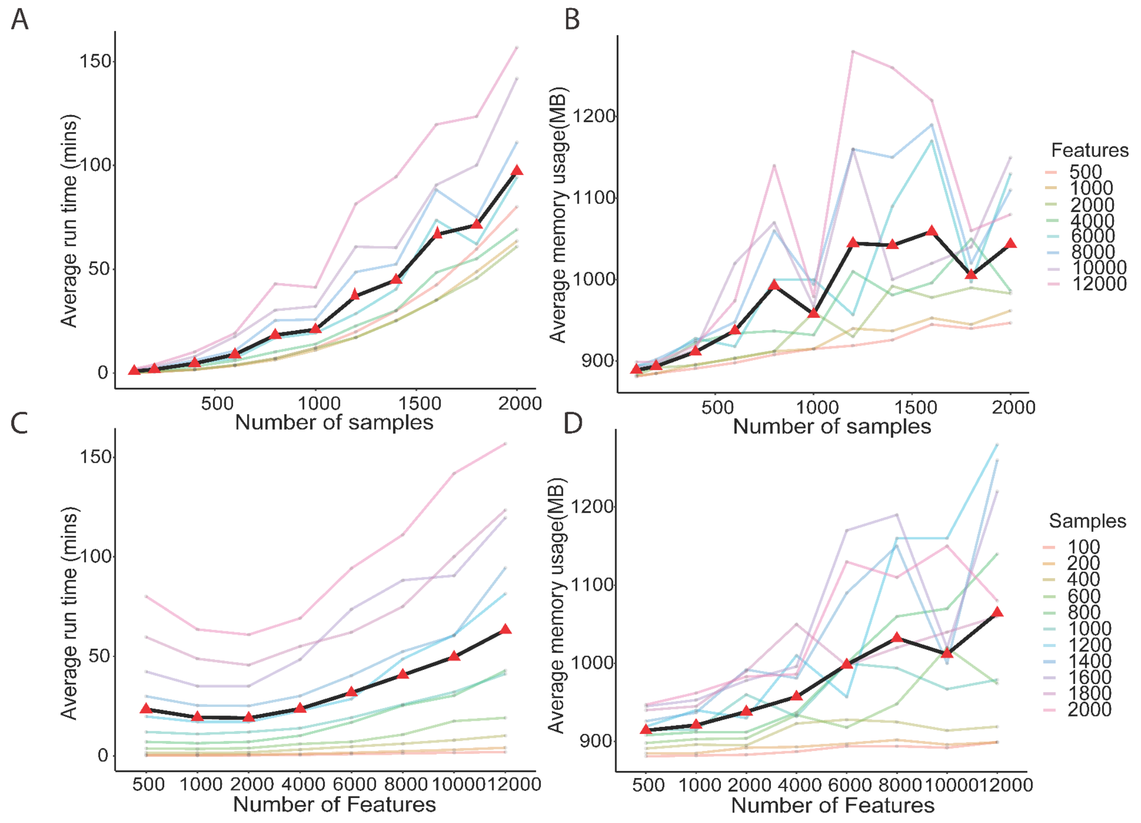

Supplementary Figure 3: Evaluating scalability performance across different scenarios on a high-dimensional single-cell PBMC dataset: (A) measuring run time and (B) memory usage as the number of samples per features vary, and (C) measuring run time and (D) memory usage as the number of features per samples vary. Black line represents average run time and memory usage across samples or across features.

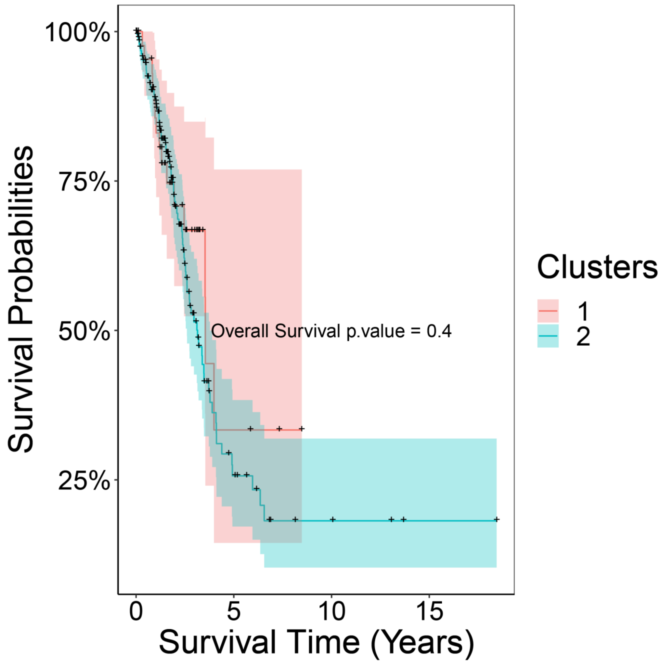

Supplementary Figure 4: Kaplan-Meier survival analysis on the 2 clusters generated by using Omada on TCGA LUAD whole transcriptome data from PMID35664309.
