# Supplementary material for: Omada: robust clustering of transcriptomes through multiple testing
Source: Gigascience. 2024 Jul 11;13:giae039. doi: 10.1093/gigascience/giae039 (PMC11238428; doi:10.1093/gigascience/giae039)
Supplement: giae039_Response_to_Reviewer_Comments_Original_Submission [file giae039_response_to_reviewer_comments_original_submission.pdf]

Author's Response To Reviewer Comments

Dear Editor,
We have now added substantial detail on methods and guidelines for reproducibility. We have also provided evidence to support the choices made, with a clear rationale behind this work, with benchmarking (compute and performance), and description of the application to one or more case studies, and discuss how the work fits into the context.

The code has been cleaned to remove any dead code and we made an update to the code available on bioconductor.

Please find a point-by-point response to all reviewers' comments in the cover letter.

Best regards,

Prof Dennis Wang
(on behalf of all co-authors)
