# Supplementary material for: Omada: robust clustering of transcriptomes through multiple testing
Source: Gigascience. 2024 Jul 11;13:giae039. doi: 10.1093/gigascience/giae039 (PMC11238428; doi:10.1093/gigascience/giae039)
Supplement: giae039_Response_to_Reviewer_Comments_Revision_1 [file giae039_response_to_reviewer_comments_revision_1.pdf]

Author's Response To Reviewer Comments

Dear Editor,

Thank you for the opportunity to respond to the single reviewer's comments. We have addressed all their comments and made extensive edits to the text to satisfy the reviewer's preferences for writing style. These include:
- Substantial re-writing of the Introduction and Discussion to present the paper more as an article describing a software package.
- More details in all sections about the purpose, rationale behind parameter choices, benchmarking across multiple different applications, and discussion about context.
- Additional functions for consensus clustering and metric for measuring agreement between multiple clustering outputs
- Additional analysis of cancer data showing agreement between clustering algorithms and parameters.

A clear point-by-point response with highlighting of the changes made can be viewed in the Personal Cover letter at the end of this submission. We hope these extensive amendments will satisfy your criteria for publication in Gigascience.

Best regards,

Dennis Wang
(on behalf of all co-authors)

Reviewer Comment: I found the manuscript difficult to read. It reads somewhat like a how-to guide and somewhat like a software package. I recommend approaching this as a software package, which would require adding evidence to support the choices made. Describe the purpose for the package, evidence for the choices made, benchmarking (compute and performance), describe application to one or more case studies, and discuss how the work fits into the context.

Response: Thank you for the honest feedback on the readability of our paper. We have now made substantial revisions to the Introduction and Discussion sections to describe our aims and conclusions for Omada as a software package.
We want to stress that Omada's objective is not to provide superior clustering algorithms compared to other methods. Instead, we aim to highlight that Omada is a toolkit equipped with various functions, metrics, and algorithms to assess datasets suitability for partitioning to the formation of clusters. It is not just about offering metrics and algorithms for selection; rather, our approach is designed to help both experienced and inexperienced users achieve justifiable clustering outputs. We also provide a number of test datasets (some previously unpublished) for users to explore different parameters and how they perform in datasets from different health conditions and tissues.

Introduction
The rapid development of next-generation sequencing boosted the quantitative analysis of gene expression in a variety of human tissues and organs1,2 generating valuable resources3 for downstream investigative analysis. In recent years, such analyses aim to elucidate disease mechanisms4 and construct genomic profiles5 to explain diagnosis6, prognosis and treatment patterns. However, transcriptomic profiles can be heterogeneous due to several causes pertaining to technical biases that produce batch effects7, cellular diversity8, disease heterogeneity9 as well as differences between individuals and populations10,11. In turn, this heterogeneity hinders research efforts aiming to identify subpopulations within complex diseases by applying unsupervised machine learning techniques12. More specifically, clustering algorithms have been applied to high-dimensional data from transcriptomic profiling13–15, and revealed novel molecular classes associated with different symptoms of disease16–18. Despite the intrinsic capabilities of these algorithms, a recurrent challenge is the insufficiency of default configurations to tailor these models precisely to unique datasets, thereby impeding the extraction of critical insights into the heterogeneity of the samples. This limitation underscores the complexity and heterogeneity inherent in biological data,

which often eludes a one-size-fits-all approach to clustering. Empirical evidence from numerous comparisons and tests reveals that the algorithms exhibit variable efficacy across different datasets and disease contexts, accentuating the necessity for model tuning or optimization specific to the dataset in question, particularly with advanced methodologies19,20,21,22. The challenge is further compounded by the possibility that the samples could be from a spectrum of states and there are no optimal metrics to group them.

Most clustering packages offer implementations of one or more algorithms without considering the previous and subsequent steps of the clustering process, such as detecting useful features or estimating the number of subgroups in the data. Since clustering consists of multiple steps, it is important to carefully approach each step so that intermediate decisions are not arbitrary but based on appropriate clustering theory. Due to this diversity in methodologies, there is a need for a toolkit that guides non-expert users through the different parameters of common clustering algorithms for their unsupervised analysis. One of the most important aspects of sample partitioning is the stability of the generated groups as unstable clusters, usually imply the lack of signal which should be present and drive the clustering of samples. Signals from genes across the transcriptome can vary across different tissues and diseases. Cluster instability can be caused inherently by the data points or by the type and application quality of a clustering technique. There lacks a robust approach that incorporates comprehensive assessment of multiple clustering methods and parameters for different sources of transcriptomic data.

Here we introduce Omada, a toolkit with multiple functions based on cluster stability and quality metrics that supports both experienced and inexperienced users from dataset exploration to the formation of the sample clusters. In contrast to other methodologies, we provided a flexible toolkit offering multiple metrics and algorithms for testing, as well as an end-to-end pipeline. Synthetic and real-world datasets from bulk tumour, single cell sequencing and whole blood have been used to test the general usability of Omada. While we cannot guarantee finding the optimal parameters, especially for healthy test samples where true clusters are unknown, we aim to provide users with guidelines based on quality metrics and empirical best practices, in order to ensure that their clustering results are robust and well justified.

Discussion

Omada is designed to enable the assessment of multiple clustering solutions arising from transcriptomic studies of human samples. With the plurality of unsupervised methods available and their specialised nature, the selection of the most appropriate approach is a multi-factor problem and suggesting a general system of recommendation is impossible without considering each dataset in advance21. Many decisions on technical parameters are required from the user of clustering approaches in order to get a meaningful set of subgroups 71. To assist with this problem, our toolkit initially assesses the potential of a target dataset and provides recommendation for the most appropriate clustering algorithm, gene set and number of possible subgroups. Omada can also be run as a pipeline, therefore, does not require deep prior knowledge of the algorithms, parameters and metrics by the user. All outputs of the pipeline, intermediate and final, are observable and each step is justified by multiple measures and indices representing widely used clustering techniques. We showed that a user of Omada can conduct analysis to assess the feasibility of applying different clustering algorithms and parameters to different RNAseq datasets from healthy and diseased tissue samples.

Conducting unsupervised learning of expression datasets is often not a straightforward task as the true heterogeneity of the health condition or tissue of origin is unknown. No methods or metrics can give a definitive answer without validation from external biological or clinical insights, therefore, the output of each algorithm has to be used with caution. Determining the dataset clustering potential is an indication rather than a clear sign that partitioning the dataset will yield informational subgroups71. Additionally, clustering can often contain non-deterministic steps (i.e. k-means initial cluster centers selection40) allowing for each function to behave slightly differently between similar runs. In order to evaluate the uncertainty and provide a robust set of tools, Omada has been applied to gene expression datasets where we have prior confidence in the number of clusters (simulated and cancer type data) and where we have little prior knowledge (GUSTO whole blood, PAH). Even in data from healthy tissue, such as from GUSTO whole blood, small technical biases can result in prominent clusters detected by Omada. It is also possible for Omada to hint towards the existence of a single homogeneous cluster by consistently revealing low partition agreement and stability scores across multiple functions, as demonstrated in our single-class datasets. Furthermore, Omada can help in selecting a small group of genes with potential partitioning capabilities as the feature selection step is expected to greatly reduce the number of genes which in most

cases count to thousands.

This toolkit currently encompasses common clustering techniques, metrics and test datasets, but its modular nature allows its extension to more specific clustering problems, such as for single cell and spatial data. Furthermore, the structure of this toolkit allows for additional algorithms and metrics to be added to the pool of clustering methods to be evaluated during an analysis run. Systematic empirical testing of algorithms using Omada's approach may result in more robust and better justified clustering outputs for a variety of biomedical studies.
We would also like to highlight benchmarking of compute time and scalability performance which was added in the last revision. In the "Test datasets" section, we added the data processing steps of PBMC single-cell use case.
In the methods section, we added a new section "Evaluation of run time and memory usage". A new section "Scalability Performances" in the result section was also added to report how Omada scales in terms of runtime and memory for simulated datasets of different sizes for both bulk RNA-seq and single-cell data use cases.
In addition to scalability performance, we also performed the benchmarking of Omada against other clustering methods and added the section "Benchmarking Omada against other clustering approaches". Furthermore, benchmarking was also performed on a high-dimensional single-cell use case where a random subset of 4 major celltype including monocytes, B cells and CD4 and CD8 T cells consisting of 1000 cells and 10000 features/genes were analysed.

To compare Omada's results to previous clustering results from cancer studies, we utilise an additional TCGA LUAD dataset from PMID 35664309. With this, we added additional guidelines on the data processing step. We added the results section under "Discriminating cancer types from pan cancer tissue expression data".

Reviewer comment: The evaluation includes two simulation studies and then application to a few real datasets; however, for all real datasets the problem is either very easy or the answer is unknown. The largest challenges I have with the manuscript are the large number of arbitrarily selected parameters the limited evidence available to support those as strong choices.

Response: We understand that real datasets can vary in complexity, ranging from being straightforward to having unknown answers. However, when the cluster memberships are known, such as in the case of two simulated datasets (single-class or multi-class with 5 classes), the Omada pipeline can be automated efficiently, and validation is trivial.
Conversely, in most datasets from human tissue, as seen in our data from pulmonary hypertension and maternal blood, descriptive statistics and parameters from the Omada pipeline need to be outputted and evaluated by the user in order to estimate what might be the true clusters. The user would need prior knowledge about the health conditions and other phenotypic information from the donors.
To provide more evidence on the choices made in our example analyses, we have made the following amendments.
Under "Sample and gene expression preprocessing", we adjusted the following text:
Various normalisations can be utilised, each catering for specific data types (i.e. arcsine transformation fit RNA-seq data, as described in section "Test datasets"). It's worth noting that Omada applies the arcsine transformation on the input data during the first step and the transformed data are carried over to subsequent steps.
In addition, we added data processing guidelines for the PBMC single-cell dataset use case:
Additionally, high-dimensional data such as the single-cell transcriptomic data should be properly quality-controlled where dying (or non-viable) cells and cells with empty droplets or multiplets should be removed. Together with normalisation, scaling, clustering and cell type specific quality control, such data pre-processing steps ensured high quality data to be considered for further analysis.

For stability based analysis, we added the following:

As for the number of genes, it is advised for larger genesets (>1000 genes) to filter down to the most variable ones (few hundreds) before the application of any function as genes that do not vary across samples do not contribute towards identifying heterogeneity. In this step the filtering should not be very

strict but rather discard genes with zero or near zero variance (i.e. housekeeping genes). In Omada, the main filtering is achieved in a later stage by identifying the set of genes that outputs the highest stability of generated clusters, as described in section "Stability-based assessment of feature sets"
On "Determining clustering potential", we added the following text:
At the start of each study, we assess the suitability of the input dataset for clustering, using the Omada package pre-pipeline analysis, to ensure general dataset attributes do not influence the process (Figure 1). The number of samples and features (e.g. genes), as well as the balance of the two dimensions, directly affects the capabilities of clustering methods to handle the dataset. An inadequate number of samples does not provide enough training power[44], while an overabundance of samples might clutter the provided information and confuse most methodologies[45]. Similarly, too few features can lead to weak clustering criteria and too many features might lead a methodology away from the features that can really differentiate between clusters of samples. Therefore, to estimate the feasibility of a clustering procedure on a specifically sized dataset we rely on measurable metrics of cluster quality, such as stability. Clusters of high stability denote both a partitionable dataset as well as a dataset-suitable methodology[46]. The feasibility score of any dataset is a function of both dimensions as well as the number of classes requested. As such, if too many or a single class is requested of a relatively small dataset the calculation will reflect low feasibility due to insufficient samples and/or features to form the desired classes. Omada's functions feasibilityAnalysis() and feasibility_analysis_based_on_data.R, can simulate datasets with dimensions similar to any dataset and calculate their stability across different numbers of clusters to determine whether such a dataset can be stable enough to be considered for clustering.
Under "Intra-method Clustering Agreement" section, we included
We implemented a tool (Figure 1) to calculate an average agreement score per clustering approach by comparing a number of runs within each of the three clustering approaches (hierarchical[47], k-means[48], spectral clustering[49]) using multiple parameters (kernels, measures, algorithms) specifically based on the data set provided which is already arcsine transformed from the first pipeline step. The aforementioned clustering methods were selected to represent fundamentally different approaches of partitioning data so as to cover several types of datasets. Hierarchical clustering is well suited for hierarchical data or data that can be represented as dendrograms, such as taxonomies (Rodriguez et al. 2019). K-means allows a different approach of partitioning samples, namely Centroid-based clustering, which is based on non-hierarchical clusters, vector quantization and representing clusters with their centroids (Rodriguez et al. 2019). Spectral clustering adds a spatial component to k-means as it utilises a similarity matrix to create a graph where clusters with irregular shapes are investigated (Rodriguez et al. 2019). Although the above approaches are not exhaustive, the modular nature of Omada allows for additional approaches to be added in subsequent versions.

We also adjusted the following text to address comparison between runs.

The number of comparisons (c), between runs of the same approach, is an additional overarching parameter of this tool and contributes to the agreement score. Each comparison requires two variations of the same algorithm. These variations are expressed as clustering parameters such as distance measures or in the case of hierarchical clustering linkage type (Table 2). A difference in such parameters produces clustering models that despite using the same algorithm have different formulas to calculate clusters. If these different formulas agree on how to partition the data then the algorithm using them demonstrates that it can capture an underlying signal in the data rather than randomly distributing data points. For each comparison, the parameters of the two runs are drawn randomly from a predefined set (Table 2) selected randomly with replacement while not allowing the same parameters to be used within one comparison. As we have to decide on a dataset without prior training on similar data, the random choice of multiple parameters is more reliable than selecting any single parameter. Additionally, in the interest of performance and computational time we suggest three comparisons to be used instead of exhaustively using every parameter.
Under "spectral clustering algorithm", we added details to the stepwise method.
Given a set of points $S = \{s_1 \dots ,s_n\}$ in R^l that we want to cluster into k subsets:
Form the affinity matrix $A \in R^{n \times n}$ defined by $A_{ij} = \exp(-||s_i - s_j||^2 / 2\sigma^2)$ if $i \neq j$, and $A_{ii} = 0$
Define D to be the diagonal matrix (where A(i,j) = 0) whose (i, i)-element is the sum of A's i-th row, and construct the matrix $L = D^{-l/2} A D^{-l/2}$
Find $x_1 , x_2 , \dots , x_k$, the k largest eigenvectors of L (chosen to be orthogonal to each other in the case of repeated eigenvalues), and form the matrix $X = [x_1\ x_2 \dots x_k] \in n \times k$ by stacking the eigenvectors in

columns
Form the matrix Y from X by renormalizing each of X's rows to have unit length (i.e. Yij = Xij / (Σj X2ij)1/2)
Treating each row of Y as a point in Rk , cluster them into k clusters via K-means or any other algorithm (that attempts to minimize distortion)
Finally, assign the original point si to cluster j if and only if row i of the matrix Y was assigned to cluster j

Under “hierarchical clustering algorithm” we added the following:

Hierarchical clustering algorithm (average linkage)
Given a set of points S = {s1 ... ,sn} that we want to cluster into k subsets:
Initialize with n clusters, each containing one data point (si)
Compute the between-cluster distance D(r, s) as the between-object distance of the two data points in clusters r and s respectively, r, s =1, 2, ..., n. Let the square matrix D = (D(r, s)). Various distances can be used (euclidean, manhattan, canberra, minkowski, maximum).
Find the most similar pair of clusters r and s, such that D(r, s) is minimum among all pairwise distances
Merge r and s to a new cluster t and compute the between-cluster distance D(t, k) for any existing cluster k ≠ r, s. Once the distances are obtained, delete the rows and columns corresponding to the old cluster r and s in the D matrix, as r and s do not exist anymore. Then add a new row and column in D corresponding to cluster t.
Repeat Step 3 a total of n − 1 times until there is only one cluster left (effectively minimising the number of redundant clusters).
Decide on a point to cut the cluster tree created above so as to obtain the desirable number of clusters (k)

In the “Stability-based assessment of feature sets” section, we added
To assess the suitability of each resampled feature set for our clustering, we measure the average stability of the clusters they generate per run when a clustering method is applied over a range of k’s (Figure 2B). First, the clustering range, where the stability of each dataset will be calculated, is selected. The lower end of this range is 2, the minimum possible number of clusters and the upper case is selected by the user. For each dataset we generate the bootstrap stability for every k within range (clusterboot function in R package fpc v2.2-3 using nonparametric bootstrap, spectral clustering and 25 resampling runs).

In the “Choosing k number of clusters”, we added the following text.

Most clustering methods require the number of k clusters to be defined as a parameter before the application of the algorithm on the data. The lack of a concrete way to determine the real number of clusters in a dataset led many studies to base their estimation on field/prior knowledge or various estimation methods such as the Silhouette score52. However, each method favours different aspects of the generated clusters (i.e. how compact clusters are and how far apart cluster centres are) and therefore suits specific datasets and may introduce bias towards the selection of k. To encompass these different angles in one methodology, avoid the risk of selecting an ineffective index and present a more general solution, this tool uses an ensemble learning approach (Figure 1) where multiple internal cluster indexes contribute to the decision making53. This approach prevents any bias from specific metrics and frees the user from making decisions on any specific metric and assumptions on the optimal number of clusters.

Initially, the value of the 15 indexes is calculated for each k within a cluster range of [2, x], where x is a logical upper limit of the number of clusters realistic for our dataset (i.e. the present conditions within a dataset). The means over k are calculated per index and the optimal k is estimated by majority voting of the 14 means that evaluate the compactness and/or the distance between different subgroups. The selection of indexes can be found in Table 3. It is important to note that the most important aspect of determining k is minimum loss of information which directs us to overestimate and not underestimate k51 while interpreting the voting results. Furthermore, cases that present only a single k as the optimal number of clusters should be treated with caution in case they are a result of a biased dataset. Omada scores each k within the set range [2, x] enabling the user to observe the most probable number of clusters according to the above criteria (function clusterVoting()). This prediction is agnostic to any other type of biological information except gene expression. However, after the clustering groups are formed it is advised for the

user to investigate whether they represent relevant to the specific case biological subgroups.”

Reviewer Comment: Conceptually, an alternative strategy is to consider real clusters to be those that are robust over many clustering methods. In this case, the best clusters are those that are maximally detectable with a single method. While there exists software for the former strategy, this package implements the latter strategy. It is not intuitively clear to me that this framework is superior to the other for biological discovery. It seems like general clusters (i.e., those that persist across multiple parameterizations) may be the most fruitful to pursue. It would be helpful to provide evidence that the selected strategy has superior utility in at least some settings and a description of how those settings might be identified. It is possible this is not necessary, but I simply note it as I continue to have these challenges with the revised manuscript.

Response: We agree that examining multiple clustering outputs and finding their agreement could be an effective strategy towards finding true cluster memberships. Since there is no standard way of aggregating multiple clustering memberships and reaching a consensus that guarantees the most robust clusters, we left it to the users to subjectively decide. Users of Omada are able to stop at each step of the pipeline and compare parameters, but we have now added functions for them to get the partition agreement between different clustering outputs and parameters tested.

We have also added the option for users to conduct consensus clustering using M3C to find the clusters that persist across multiple parameterizations, as you have suggested. We caution them to still use human judgement by showing an example of clustering cancer types where there may not be a single clustering run that generates the most likely number of clusters.

Methods

Exploring the number of clusters (k) when using single algorithms versus consensus clustering

In this section we test whether, in RNA-seq clustering tasks, a single clustering algorithm can be a superior choice than other algorithms which represent different approaches. Towards this goal we extract gene expression datasets from the Cancer Genomics cBioPortal (https://www.cbioportal.org/) and test the extent to which different algorithms can recognise different cancer types. The first dataset consists of three cancer types: Esophageal Adenocarcinoma (esca, n = 181) , Thymoma (thym, n = 119) and Testicular Germ Cell Tumors (tgct, n = 149). The 20,531 genes were first filtered to exclude duplicates as well as those without expression values for the majority of samples. They were subsequently ranked, in a descending manner, for variance across samples and then 500 most variant genes were included in the clustering models. The dataset was normalised using the inverse hyperbolic sine (function asinh in base R package). The second dataset was simulated based on exact dimensions of the aforementioned cancer dataset using Omada’s simulation function feasibilityAnalysisDataBased() with 3 distinct classes. Spectral, kmeans and hierarchical clustering algorithms were tested alongside a consensus clustering method M3C from package https://www.bioconductor.org/packages/release/bioc/html/M3C.html. Default parameters were used for each algorithm. The adjusted Rand index was calculated between the set of generated memberships of the top k estimate and the true cancer type labels of the samples. Additionally, we registered the k estimation of each methodology against the real number of different cancer types for each dataset. To estimate k for hierarchical, spectral and k-means the Omada ensemble method was used, while for M3C the provided Relative Cluster Stability Index (RCSI).

Results

Not all algorithms can detect the true number of clusters

We tested the ability of four algorithms to cluster samples that belong to three cancer types. As seen on Table X1, hierarchical clustering was not able to identify the three cancer types (ARI = 0.55) as it selected two clusters as most probable. On the contrary, spectral clustering separated the samples most accurately

(ARI = 0.83) with a clear preference for three clusters (8/14 votes) closely followed by k-means (ARI = 0.80 and 5/14 votes). Consensus clustering followed with an ARI of 0.78 but a widest spread of estimated k as it selected 5 and 6 clusters as second and third choices respectively. On the simulated dataset (Table X2), k-means estimated the correct number of clusters and managed to assign the memberships perfectly (ARI = 1). All other algorithms selected a different k and therefore presented a much lower ARI (as comparing a set of two clusters with a set of three clusters cannot result in a high ARI). Both Omada and consensus clustering M3C suggest k = 3 as the second estimate. However, Omada already supplies the memberships for k = 3 as part of the k estimation achieving a perfect ARI of 1 if that partitioning is selected for all three algorithms.

Table 4: The ARI of the top k estimate of various datasets with different numbers of cancer types

Algorithm	Real clusters		
	3	5	7
Hierarchical	0.55 (k=2)	0.13 (k=2)	0.19 (k=2)
k-means	0.80 (k=3)	0.54 (k=4)	0.45 (k=)
Spectral	0.83 (k=3)	0.84 (k=5)	0.58 (k=)
M3C	0.78 (k=3)	0.85 (k=5)	0.63 (k=9)

Table 5: The ARI of the top k estimate of various datasets with different numbers of simulated classes

Algorithm	Real clusters		
	3	5	7
Hierarchical	0.57 (k=2)	0.9 (k=7)	0.9 (k=9)
k-means	1.00 (k=3)	1.00 (k=5)	0.79 (k=8)
Spectral	0.57 (k=2)	1.00 (k=5)	1.00 (k=7)
M3C			

0.57 (k=2)
0.78 (k=5)
0.54 (k=4)
