# Supplementary material for: Omada: robust clustering of transcriptomes through multiple testing
Source: Gigascience. 2024 Jul 11;13:giae039. doi: 10.1093/gigascience/giae039 (PMC11238428; doi:10.1093/gigascience/giae039)
Supplement: giae039_Response_to_Reviewer_Comments_Revision_2 [file giae039_response_to_reviewer_comments_revision_2.pdf]

Author's Response To Reviewer Comments

Dear Editor,

Thank you for the final suggestions for revisions to the manuscript. Below are our responses to all the comments.

Comment:
Please structure Abstract into 3 x sections: Background; Findings and Conclusions.

Response:
Abstract has been reformatted.

Comment:
Please add RRID here in brackets after first meniton of Omada in the Methods.

Response:
RRID has been added.

Comment:
Change header to
"Availability of supporting source code and requirements"

Structure the information we require as follows and add the RRID number. Register you tool at scicrunch.org.

Project name: e.g. My bioinformatics project
Project home page: e.g. https://github.com/ISA-tools
Operating system(s): e.g. Platform independent
Programming language: e.g. Java
Other requirements: e.g. Java 1.3.1 or higher, Tomcat 4.0 or higher
License: e.g. GNU GPL, FreeBSD etc.
RRID: if applicable, e.g. RRID: SCR_014986
bio.tools ID: if applicable, e.g. bio.tools ID :GERONIMO

Response:
The code availability section has been reformatted and registered on scicrunch.org.

Comment:
Move all URLs to the references list and only cite the Ref # here. Add as much accession numbers as possible.

Response:
Replaced all URLs with citations from the references list. Added accession numbers.

Comment:
Add a final sentence here to read as follows:

Snapshots of our code and other data further supporting this work are openly available in the GigaScience repository, GigaDB [xx Ref#].

Response:
Added sentence with GigaDB ref.

Comment:
Please carefully check our instructions for authors and add the missing sections:

See: https://academic.oup.com/gigascience/pages/technical_note

Missing sections to add in the exact order:

List of abbreviations

Declarations:
Ethics approval and consent to participate
Consent for publication
Competing interests
Funding (please use CAsRAI Credit format)
Authors' contributions
Acknowledgments (last section prior to References)

Response:
All sections added and in the suggested order.

Comment:
Please move URLs from the main text to the references and renumber. Only add the Ref # in the main text.

Please also add a final Reference to the GigaDB DOI citation in the exact format as follows - add the Ref # under "Data availability" as noted above.

Kariotis S; Tan PF; Lu H; Rhodes CJ; Wilkins MR; Lawrie A; Wang D: Supporting data for "Omada: Robust clustering of transcriptomes through multiple testing" GigaScience Database. 2024. https://doi.org/10.5524/102537

Response:
Reference list has been updated.

Reviewer #3 Comment:
The authors describe Omada, which is software to cluster transcriptomic data using multiple methods. The approach selects a heuristically best method from among those tested. The manuscript does describe a software package and there is evidence that the implementation works as described. The manuscript structure was substantially easier for me to follow with the revisions. The manuscript does not have evidence that the method outperforms other potential approaches in this space. It is not clear to me if this is or is not an important consideration for this journal. The form requires that I select from among the options offered. Given that this requires editorial assessment, I have marked "Minor Revision" but I do not feel a minor revision is necessary if, with the present content of the paper, the editor feels it is appropriate. If a revision is deemed necessary, I expect it would need to be a major one.

Response:
We are pleased that the manuscript is more readable. We have attempted to qualitatively compare our method with other similar toolkits and highlighted our advancements. Benchmarking performance would be difficult because the usability of a clustering toolkit on real-world datasets is quite subjective. We would

have to conduct a large trial and survey users to get further feedback. It is also difficult to choose gold-standard datasets to test the methods because it is usually possible to find a dataset that clusters well with each of the algorithms. With our toolkit, even if the automated algorithm does not suggest the most likely cluster memberships, the user is provided with important information to make an informed decision on choosing the outputs for downstream analysis.

Sincerely,

Dennis Wang
Corresponding author
