# Supplementary material for: Omada: robust clustering of transcriptomes through multiple testing
Source: Gigascience. 2024 Jul 11;13:giae039. doi: 10.1093/gigascience/giae039 (PMC11238428; doi:10.1093/gigascience/giae039)
Supplement: giae039_Reviewer_2_Report_Original_Submission — Pierre Cauchy -- 10/26/2023 Reviewed [file giae039_reviewer_2_report_original_submission.pdf]

Reviewer Report

Title: Omada: Robust clustering of transcriptomes through multiple testing

Version: Original Submission **Date:** 10/26/2023

Reviewer name: Pierre Cauchy

Reviewer Comments to Author:

Kariotis et al present Omada, a tool dedicated to automated partitioning of large-scale, cohort-based RNA-Sequencing data such as TCGA. A great strength for the manuscript is that it clearly shows that Omada is capable of performing partitioning from PanCan into BRCA, COAD and LUAD (Fig 5), and datasets with no known groups (PAH and GUSTO), which is impressive and novel. I would like to praise the authors for coming up with such a tool, as the lack of a systematic tool dedicated to partitioning TCGA-like expression data is indeed a shortcoming in the field of medical genomics Overall, I believe the tool will be very valuable to the scientific community and could potentially contribute to meta-analysis of cohort RNA-Seq data. I only have a few comments regarding the methodology and manuscript. I also think that it should be more clearly stated that Omada is dedicated to large datasets (e.g. TCGA) and not differential expression analysis. I would also suggest benchmarking Omada to comparable tools via ROC curves if possible (see below).

Methods:

This section should be a bit more homogeneous between text descriptive and mathematical descriptive. It should specify what parts are automated and what part needs user input and refer to the vignette documentation. I also could not find the Omada github repository.

Sample and gene expression preprocessing: To me, this section lacks methods/guidelines and only loosely describes the steps involved.

"numerical data may need to be normalised in order to account for potential misdirecting quantities" - which kind of normalisation?

"As for the number of genes, it is advised for larger genesets (>1000 genes) to filter down to the most variable ones before the application of any function as genes that do not vary across samples do not contribute towards identifying heterogeneity"

What filtering is recommended? Top 5% variance? 1%? Based on what metric?

Determining clustering potential: To me, it was not clear if this is automatically performed by Omada and how the feasibility score is determined.

Intra-method Clustering Agreement: Is this from normalised data? Because affinity matrix will be greatly affected whether it's normalised or non-normalised data as the matrix of exponential(-normalised gene distance)^2

Spectral clustering step 2: "Define D to be the diagonal matrix whose (i, i)-element is the sum of A's i-th row": please also specify that A(i,j) is 0 in this diagonal matrix.

Please also confirm which matrix multiplication method is used, product or Cartesian product? Also if there are 0 values, NAs will be obtained in this step.

Hierarchical clustering step 5: "Repeat Step 3 a total of n âˆ’ 1 times until there is only one cluster left."

This is a valuable addition as this merges identical clusters, the methods should emphasise that the benefits of this clustering reduction method to help partition data, i.e. that this minimises the number of redundant clusters.
Stability-based assessment of feature sets: "For each dataset we generate the bootstrap stability for every k within range". Here it should be mentioned that this is carried out by clusterboot, and the full arguments should be given for documentation
"The genes that comprise the dataset with the highest stability are the ones that compose the most appropriate set for the downstream analysis" - is this the single highest or a gene list in the top n datasets? Please specify.
Choosing k number of clusters: "This approach prevents any bias from specific metrics and frees the user from making decisions on any specific metric and assumptions on the optimal number of clusters.". Out of consistency with the cluster reduction method in the "intra-clustering agreement" section which I believe is a novelty introduced by Omada, and within the context of automated analysis, the package should also ideally have an optimized number of k-clusters. K-means clustering analysis is often hindered due to the output often resulting in redundant, practically identical clusters which often requires manual merging. While I do understand the rationale described there and in Table 3, in terms of biological information and especially for deregulated genes analysis (e.g. row z-score clustering), should maximum k also not be determined by the number of conditions, i.e 2n, e.g. when n=2, kmax=4; n=3, kmax=8?
Test datasets and Fig 6: Please expand on how the number of features 300 was determined. While this number of genes corresponds to a high stability index, is this number fixed or can it be dynamically estimated from a selection (e.g. from 100 to 1000)?
Results
Overall this section is well written and informative. I would just add the following if applicable:
Figure 3: I think this figure could additionally include benchmarking, ROC curves of. Omada vs e.g. previous TCGA clustering analyses (PMID 31805048)
Figure 4: I think it would be useful to compare Omada results to previous TCGA clustering analyses, e.g. PMID 35664309
Figure 6: swap C and D. Why is cluster 5 missing on D)?

Methods

Are the methods appropriate to the aims of the study, are they well described, and are necessary controls included? Choose an item.

Conclusions

Are the conclusions adequately supported by the data shown? Choose an item.

Reporting Standards

Does the manuscript adhere to the journal’s guidelines on minimum standards of reporting? Choose an item.

Choose an item.

Statistics

Are you able to assess all statistics in the manuscript, including the appropriateness of statistical tests used? Choose an item.

Quality of Written English

Please indicate the quality of language in the manuscript: Choose an item.

Declaration of Competing Interests

Please complete a declaration of competing interests, considering the following questions:

- Have you in the past five years received reimbursements, fees, funding, or salary from an organisation that may in any way gain or lose financially from the publication of this manuscript, either now or in the future?
- Do you hold any stocks or shares in an organisation that may in any way gain or lose financially from the publication of this manuscript, either now or in the future?
- Do you hold or are you currently applying for any patents relating to the content of the manuscript?
- Have you received reimbursements, fees, funding, or salary from an organization that holds or has applied for patents relating to the content of the manuscript?
- Do you have any other financial competing interests?
- Do you have any non-financial competing interests in relation to this paper?

If you can answer no to all of the above, write 'I declare that I have no competing interests' below. If your reply is yes to any, please give details below.

I declare that I have no competing interests

I agree to the open peer review policy of the journal. I understand that my name will be included on my report to the authors and, if the manuscript is accepted for publication, my named report including any attachments I upload will be posted on the website along with the authors' responses. I agree for my report to be made available under an Open Access Creative Commons CC-BY license (http://creativecommons.org/licenses/by/4.0/). I understand that any comments which I do not wish to be included in my named report can be included as confidential comments to the editors, which will not be published.

Choose an item.

To further support our reviewers, we have joined with Publons, where you can gain additional credit to further highlight your hard work (see: https://publons.com/journal/530/gigascience). On publication of this paper, your review will be automatically added to Publons, you can then choose whether or not to claim your Publons credit. I understand this statement.

Yes Choose an item.
