# Supplementary material for: Omada: robust clustering of transcriptomes through multiple testing
Source: Gigascience. 2024 Jul 11;13:giae039. doi: 10.1093/gigascience/giae039 (PMC11238428; doi:10.1093/gigascience/giae039)
Supplement: giae039_Reviewer_3_Report_Revision_1 — Casey S. Greene -- 2/12/2024 Reviewed [file giae039_reviewer_3_report_revision_1.pdf]

Reviewer Report

Title: Omada: Robust clustering of transcriptomes through multiple testing

Version: Revision 1 **Date:** 2/12/2024

Reviewer name: Casey S. Greene

Reviewer Comments to Author:

The authors have revised their manuscript. They added benchmarking for the method, which is important. The following overall comments still apply - there is not substantial evidence provided for the selections made:
"I found the manuscript difficult to read. It reads somewhat like a how-to guide and somewhat like a software package. I recommend approaching this as a software package, which would require adding evidence to support the choices made. Describe the purpose for the package, evidence for the choices made, benchmarking (compute and performance), describe application to one or more case studies, and discuss how the work fits into the context.
The evaluation includes two simulation studies and then application to a few real datasets; however, for all real datasets the problem is either very easy or the answer is unknown. The largest challenges I have with the manuscript are the large number of arbitrarily selected parameters the limited evidence available to support those as strong choices.
Conceptually, an alternative strategy is to consider real clusters to be those that are robust over many clustering methods. In this case, the best clusters are those that are maximally detectable with a single method. While there exists software for the former strategy, this package implements the latter strategy. It is not intuitively clear to me that this framework is superior to the other for biological discovery. It seems like general clusters (i.e., those that persist across multiple parameterizations) may be the most fruitful to pursue. It would be helpful to provide evidence that the selected strategy has superior utility in at least some settings and a description of how those settings might be identified."
It is possible this is not necessary, but I simply note it as I continue to have these challenges with the revised manuscript.

Methods

Are the methods appropriate to the aims of the study, are they well described, and are necessary controls included? Choose an item.

Conclusions

Are the conclusions adequately supported by the data shown? Choose an item.

Reporting Standards

Does the manuscript adhere to the journal's guidelines on minimum standards of reporting? Choose an item.

Choose an item.

Statistics

Are you able to assess all statistics in the manuscript, including the appropriateness of statistical tests used? Choose an item.

Quality of Written English

Please indicate the quality of language in the manuscript: Choose an item.

Declaration of Competing Interests

Please complete a declaration of competing interests, considering the following questions:

- Have you in the past five years received reimbursements, fees, funding, or salary from an organisation that may in any way gain or lose financially from the publication of this manuscript, either now or in the future?
- Do you hold any stocks or shares in an organisation that may in any way gain or lose financially from the publication of this manuscript, either now or in the future?
- Do you hold or are you currently applying for any patents relating to the content of the manuscript?
- Have you received reimbursements, fees, funding, or salary from an organization that holds or has applied for patents relating to the content of the manuscript?
- Do you have any other financial competing interests?
- Do you have any non-financial competing interests in relation to this paper?

If you can answer no to all of the above, write 'I declare that I have no competing interests' below. If your reply is yes to any, please give details below.

I declare that I have no competing interests

I agree to the open peer review policy of the journal. I understand that my name will be included on my report to the authors and, if the manuscript is accepted for publication, my named report including any attachments I upload will be posted on the website along with the authors' responses. I agree for my report to be made available under an Open Access Creative Commons CC-BY license (http://creativecommons.org/licenses/by/4.0/). I understand that any comments which I do not wish to be included in my named report can be included as confidential comments to the editors, which will not be published.

Choose an item.

To further support our reviewers, we have joined with Publons, where you can gain additional credit to further highlight your hard work (see: https://publons.com/journal/530/gigascience). On publication of this paper, your review will be automatically added to Publons, you can then choose whether or not to claim your Publons credit. I understand this statement.

Yes Choose an item.
